# Readiness Assessment for AI in Nursing Care Projects: Multimethods Study

**DOI:** 10.2196/84148

**Published:** 2026-06-02

**Authors:** Kathrin Seibert, Dominik Domhoff, Janissa Altona, Sebastian Jäger, Felix Bießmann, Alessia Nowak, Rahel Gubser, Matthias Schulte-Althoff, Daniel Fürstenau, Jörg Pohle, Lea Bergmann, David Walter, Kathi Beier, Dagmar Borchers, Karin Wolf-Ostermann

**Affiliations:** 1 Institute of Public Health and Nursing Research, University of Bremen, Universitätsallee 1B-C, Bremen, 28359, Germany, 49 42121868903 ext 21868903; 2 Faculty VI Information Science and Media, Berliner Hochschule für Technik (BHT), Berlin, Germany; 3 Institute of Medical Informatics, Charité Universitätsmedizin, Berlin, Germany; 4 Alexander von Humboldt Institute for Internet and Society, Berlin, Germany; 5 Association for Digitalisation in the Social Sector e.V., Halle (Saale), Germany; 6 Institute of Business Administration & Information Systems, University of Hildesheim, Hildesheim, Germany; 7 Institute for Philosophy, University of Bremen, Bremen, Germany

**Keywords:** artificial intelligence, nursing, care, readiness, maturity model

## Abstract

**Background:**

Integrating artificial intelligence (AI) systems into nursing care often encounters obstacles stemming from unmet requirements and insufficient engagement with well-documented sociotechnical pitfalls. Readiness models offer a systematic way to evaluate project preparedness and to build the capabilities needed for successful artificial intelligence in nursing care (AINC) research, development, and implementation. As of yet, an evidence-based AI readiness assessment prioritizing AINC projects and accounting for their diversity in care settings is missing.

**Objective:**

This study aimed to develop a comprehensive artificial intelligence nursing care readiness assessment (AINCRA) to support planning, execution, and evaluation of AINC projects.

**Methods:**

In a sequential exploratory multimethods bottom-up approach to maturity model development, key AI readiness dimensions and attributes were identified to develop a pilot readiness assessment. The pilot version was grounded on insights from an expert workshop (n=21) and expert interviews (n=14), an online survey (n=53), a rapid review (n=292), and a nominal group consensus process. A systematic literature review (n=7) further triangulated AI readiness attributes. Finally, a think-aloud interview study and focus group discussions involving experts (n=18) from nursing practice, nursing science, and AI research and development who had conducted AINC projects prior to data collection validated the attributes.

**Results:**

The resulting AINCRA encompasses 5 core dimensions: regulatory, processual, technical, social, ethical, and community building requirements and aspects. Including 69 attributes and capabilities of AI nursing care readiness, the core dimensions reflect key areas of action where AINC project stakeholders can influence project outcomes. Clinical partners can assess their organization’s maturity level in relation to the implementation of AI. An assessment of each dimension and its attributes across 5 maturity levels allows reflecting on and proactively shaping individual project approaches. Overall, experts regarded AINCRA as a useful instrument for the development, management, and evaluation of AINC projects while emphasizing that established principles of good practice in project and data management should not be neglected when using AINCRA as a project management tool.

**Conclusions:**

AINCRA enables practitioners from AI research and development, clinical partners, and nursing and health scientists to plan, evaluate, and enhance AI projects across their lifecycle, thereby supporting effective AI integration in nursing care. While AINCRA was developed within the European and German legal framework for AI in health care settings, respective attributes can be adapted to international requirements.

## Introduction

Artificial intelligence (AI) systems promise to contribute to process optimization, workload reduction, patient safety, and quality assurance in nursing care worldwide [1-3]. Following the European Commission’s guidelines on AI system definition as entailed in the European Union’s Artificial Intelligence (EU AI) Act, AI systems in nursing care refer to machine-based systems developed to function with different degrees of autonomy and potentially adapt over time after being deployed [4]. AI systems process input data to produce outputs such as predictions, content, recommendations, or decisions based on explicit or implicit goals, and these outputs can affect physical or digital environments [4]. Main AI techniques encompass machine learning (ML) approaches including methods such as supervised learning, unsupervised learning, self-supervised learning, and reinforcement learning, or deep learning [4]. Additionally, AI systems include logic- and knowledge-based approaches that infer from encoded human knowledge or symbolic representation of the task to be solved [4]. Logic- and knowledge-based methods encompass areas such as knowledge representation, inductive logic programming, inference engines, expert systems, symbolic reasoning, and search and optimization techniques [4], highlighting diverse methodological possibilities for the development of AI systems in nursing care. Currently, opportunities for AI system development arising from applying generative AI models specifically trained on electronic medical record (or nursing record) data [5] further expand the scope of methods and use cases.

While AI is already supporting clinical decision-making of nurses in wound assessment [6] or diabetes care [7], monitoring and detection of risks and clinical deterioration throughout the care process [8-10], or nursing documentation via hybrid speech assistants [11,12], many unexplored use cases in acute and long-term care persist [13]. Furthermore, studies aiming to assess the effectiveness of AI systems in nursing care under real-world conditions using designs capable of determining cause-and-effect relationships are still scarce [14,15]. The potential benefits and expectations of artificial intelligence in nursing care (AINC) are high and are emphasized by professional associations and the World Health Organization, in light of growing global challenges to ensure high-quality care despite a declining number of health care professionals and increasingly complex disease and care trajectories of patients [16,17]. Funding bodies are investing in AI research and development (R&D), and the number of AINC projects is growing. In Germany, for example, the Federal Ministry of Health and the Federal Ministry of Research, Technology and Space support AINC projects in the areas of new care models, health care research, assistance of nurses and family caregivers, and enhancing autonomy and quality of life of people in need of care.

AINC projects face not only technical and regulatory requirements (eg, aspects of interoperability, compliance with national or international data laws, such as privacy and data protection laws, medical device regulations, or the EU AI Act) but also procedural, ethical, and social challenges of AI development and deployment (eg, the micro-, meso-, and macro-level impact of AI on nursing care, ethical-normative values of nursing and care, and translational factors such as acceptance of AI or added practical value) [14,18,19]. This applies to the planning, implementation, and evaluation phases of AINC projects. Project leaders encounter different care settings with many possible use cases for AI (such as AI systems for care in hospitals, nursing homes, outpatient care, or in initial, further, and continuing education) [2], with each care setting influencing the project process with its own unique organizational logic and culture. AI systems may be more complex to implement and use than many other digital technologies [20]. Organizations that aim to develop, deploy, and use AI systems face technical and human-centered challenges that can be overcome by building AI maturity and ensuring they are well-prepared for AI within their organizational context [20-22]. We propose that the same holds true for AINC projects, for which a systematic, comprehensive reflection of AI readiness attributes across various dimensions can help align, justify, and document actions and strategies across all project stages, and may serve as a motivation to foster communication and collaboration within interprofessional project consortia.

Maturity or, often synonymously used, readiness models provide structured guidance to enhance capabilities for effective (AI) technology integration [20,23,24]. Transferred from insights on organizations, readiness models provide features and guidance that help AINC projects to consider context-specific factors while not overlooking humanistic objectives and aspects of sociotechnical interplay next to focusing on technical and regulatory requirements [20,21]. There is a growing body of both theoretical and empirical research on readiness and maturity models [24]. Among these, stage growth models are particularly prevalent in information systems research [24]. These models typically define a progression of maturity levels, applied to an organization or process, ranging from minimal capability to full maturity, outlining an expected or ideal development path, accompanied by a measurement instrument [23,24]. As an example, in hospitals, the maturity model for ML systems [25] is structured around 3 dimensions: organization, adopter system, and patient data. These are broken down into 12 attributes and assessed across 5 maturity levels [25]. The model addresses aspects such as ML strategy, technical infrastructure, ML expertise, user acceptance, and the quality and standardization of patient data [25]. However, so far, an evidence-based AI readiness assessment prioritizing AINC projects and accounting for their diversity beyond R&D on ML in diverse nursing care settings is missing. Hence, the objective for our research is to develop a comprehensive artificial intelligence nursing care readiness assessment (AINCRA) tool for planning, conducting, and evaluating AINC projects responsibly. Therefore, in a sequential exploratory multimethods study, we ask the following questions:

1. What are dimensions, attributes, and levels of a readiness assessment intended to support those responsible for AINC projects as a reflection tool during the planning, implementation, and evaluation phases of their projects?
2. How do experts with practical experience in planning, implementing, and evaluating AINC projects understand and use the AINCRA, and how do they assess it with regard to selected evaluation criteria?

We present AINCRA as a tool for decision-makers in R&D and clinical partners in AINC projects to reflect on their handling of prerequisites and aspects of these projects and for designing AINC projects in a promising and successful way. Further, we compare with recommendations for the development of maturity models and common criticisms of maturity models [24] to highlight methodological strengths and weaknesses of AINCRA.

## Methods

### Overview

**Figure 1.**
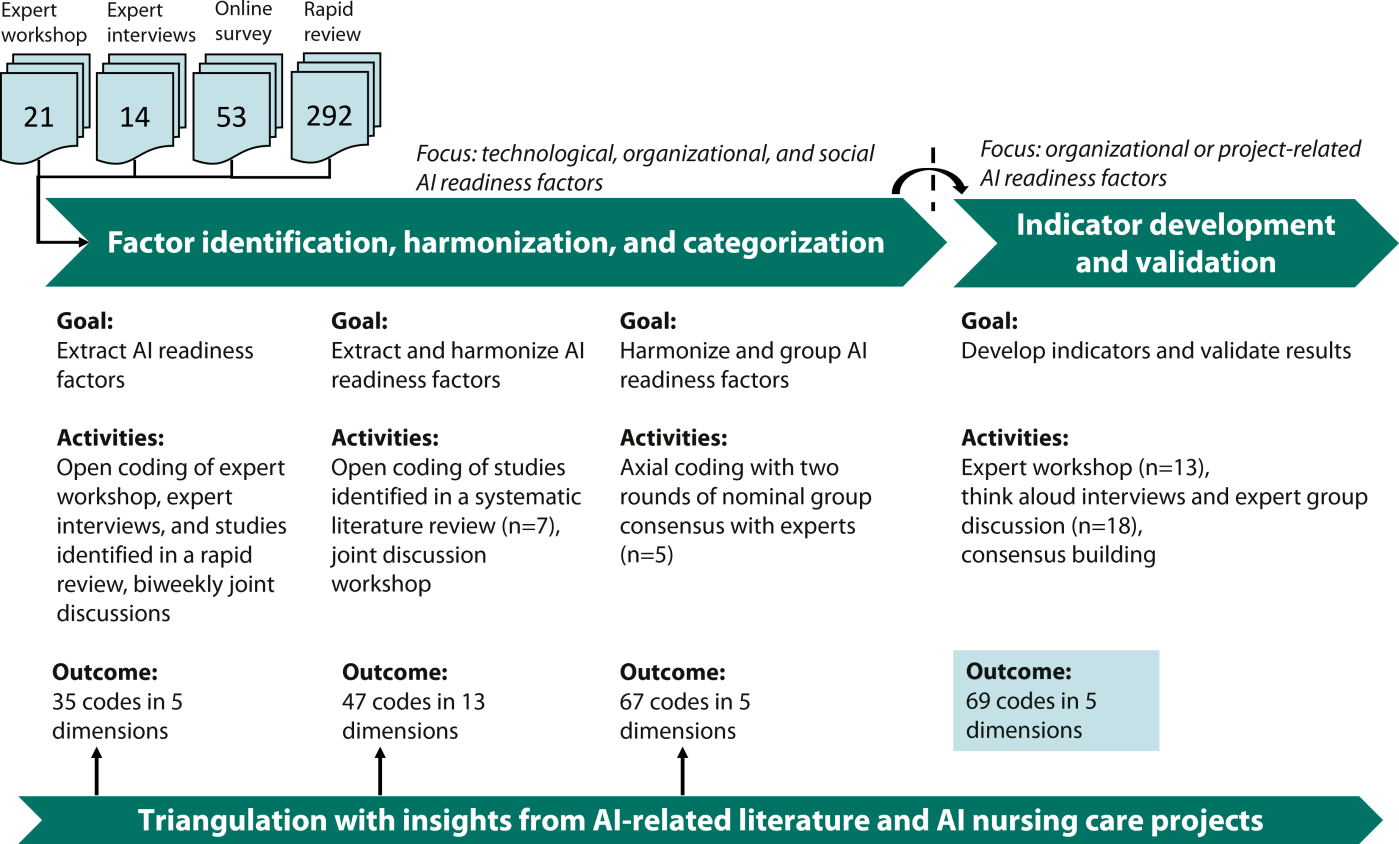
Overview of the research approach. AI: artificial intelligence.

We conducted a sequential exploratory multimethods study following established design recommendations and structures of maturity models [20,23,24] to develop AINCRA. Figure 1 presents the research approach, highlighting goals, activities of data collection, analysis, and outcomes for the study period lasting from December 2021 to March 2025. In a stepwise bottom-up approach, we categorized AI readiness factors into capabilities, applying an iterative validation process. Building on preliminary qualitative and quantitative findings from our research group [3,13], we extracted AI readiness factors and conducted a complementary systematic literature review to inform the development of an initial AINCRA version as well as guiding questions for subsequent think-aloud interviews and discussions with experts. Additionally, we triangulated results and findings from the different steps of data collection with insights from AI-related literature and from 8 AINC projects we had been involved in or which we had been accompanying during the last 5 years by repeatedly comparing preliminary drafts of AINCRA with documented project plans to assess completeness.

We follow the PRISMA (Preferred Reporting Items for Systematic Reviews and Meta-Analyses) 2020 statement for reporting systematic reviews (Checklist 1) [26]; furthermore, the COREQ (Consolidated Criteria for Reporting Qualitative Research; Checklist 2) [27] and criteria on mixed methods reporting [28] guide this research where applicable. A short description of the rationale, basic methods of development, and a short generalized overview of the AINCRA dimensions has been published elsewhere [29].

### Systematic Literature Review: Information Sources, Eligibility Criteria, and Review Protocol

Leading health and information sciences databases Scopus, PubMed, ACM Digital Library, AIS Electronic Library, and EconLit were searched in February 2023 for English- or German-language articles published from 2012 onward which either refer to a specific AI readiness assessment tool (defined by containing at least one specific question or evaluation criterion of AI readiness or maturity with a qualitative assessment option) or refer to frameworks or single studies that theoretically or empirically define AI readiness. Further, articles had to refer to AI readiness of institutions, organizations, projects, teams, or higher-level units (sectors and disciplines). Health care and nursing settings differ from other business sectors and societal areas, among other things, in that they often process highly sensitive personal data and aim to make it usable for AI systems. In addition, they are based on different sociocultural and organizational logics compared to, for example, the agricultural or logistics sector or the timelines and work culture of data science [30]. Therefore, following the assumption that AI readiness models established for other sectors cannot be transferred directly to the nursing care context, publications had to refer to nursing or health care settings. Eligibility criteria were established before conducting the initial searches, and a review protocol was developed but not registered.

### Search Strategy and Selection Process

The search strategy followed a block building approach [31] applied to title, abstract, and keyword searches using the Boolean operator OR within a single block and the Boolean operator AND across blocks to combine the search terms and synonyms for the blocks encompassing “artificial intelligence,” “readiness,” and “assessment” are depicted in Multimedia Appendix 1. Two independent reviewers (KS and DW) screened titles, abstracts, and full texts according to predefined inclusion and exclusion criteria using the online resource Rayyan [32]. Conflict between reviewers was resolved by including a third reviewer (DD).

### Data Collection Process and Data Items

Data extraction was carried out by one reviewer (KS) in a data extraction form created in Microsoft Excel for this purpose. Data items extracted from each included study entail the following: reference (author, year of publication, and title), country in which the study data were collected, objective of study, study design as stated, setting as stated; population: type and sample size (N); definition of AI: presented (yes or no), definition as stated; AI readiness: addressed (yes or no), definition as stated, explicit readiness assessment presented (yes or no), level of application (organization, project, team, and setting), health or nursing care addressed (yes or no), reporting of attribute origin (yes or no), origin of attributes as stated, number of attributes, description or labels of attributes, and labels of dimensions; readiness framework: presented (yes or no), name as stated, empirically tested (yes or no); success factors and challenges: reported (yes or no), success factors and challenges as stated; limitations as stated; ethics vote obtained: yes or no; funding; conflicts of interest. Missing data were coded as not reported.

### Study Risk of Bias Assessment, Effect Measures, and Synthesis Methods

As a conclusion on the effectiveness of interventions was not an intended result of the systematic literature review, we did not conduct a risk of bias assessment and other assessments associated with good practice of methodology for meta-analysis. Results were summarized narratively after inductively developing structuring categories for readiness descriptions and dimensions, as well as for readiness attributes. Key findings are described narratively in the results section and supplemented with tables and figures where appropriate.

### Think-Aloud Interviews and Group Discussions

Think-aloud interviews and group discussions were conducted online and digitally recorded with OBS Studio (version 29.1.3; OBS Project) in December 2024 and January 2025. Due to individual availability, 3 experts did not participate in a group discussion but answered group discussion guiding questions (Multimedia Appendix 1) in an interview following the think-aloud task. Details about the involved researcher’s background and relationship with participants are provided in Multimedia Appendix 1.

### Selection and Recruitment of Expert Participants

We applied a purposive sampling approach [33] by sending personalized, electronic invitations to 42 experts who had either been actively involved in AINC bar camps held by our study team in the past or had conducted and published AINC-related R&D in Germany and were known to the study team. A total of 19 of 42 experts replied (response rate of 45.24%), and 17 experts consented to participation. The reason for nonparticipation was unavailability during the data collection phase. Additionally, all project leads of AINC projects in the German funding program “Making Repositories and AI Systems Usable in Everyday Care” were contacted via email and invited to participate or invite members of their project teams to participate. The possibility for participation was also advertised in the newsletter for said funding program, resulting in recruitment of one participant. We also asked the funding body to propose additional experts but did not receive a response.

### Ethical Considerations

The ethics committee of the German Society of Nursing Science granted ethical clearance and approved this study (application 22‐030). Participation was voluntary, and participants received no monetary or immaterial incentives or compensation. After receiving written information including statements on data protection, privacy, and confidentiality as well as concordance with the European General Data Protection Regulation, all participants provided a written informed consent to participation as well as to the pseudonymized analysis of the data obtained and to the publication of anonymized results. The study was not of an interventional nature. Participants were considered a nonvulnerable population, and participation was not classified as of particular risk.

### Data Collection

An interview guideline structured and semistandardized the data collection process, which started with a 5-minute introduction of the interviewers, including their academic degree, field of research, and personal involvement in the research project, followed by an overview of the data collection process, an explanation of data protection, consent, and voluntary participation. After that, participants were asked to introduce themselves (name, work setting, and focus) and provide a short statement on their experiences with AINC projects and implementing AINC settings. A 10-minute introduction to the objectives of the study and AINCRA, during which participants were able to ask questions for clarification, made way for the 30-minute think-aloud interview. Participants were assigned to online breakout rooms with 1 or 2 members of the study team who also took field notes. The AINCRA dimension to be discussed was accessible on a Miro Board on which participants could navigate freely. Using the prompt outlined in the “Results” section, participants reflected on an AINC project they either had conducted, were conducting at the time of data collection, or were planning. They were asked to imagine that they are alone in the room and to talk to themselves. All thoughts were allowed to be expressed. If they were silent for a longer period, the interviewer asked them to continue to speak. Concurrent verbal probing [34] completed the think-aloud interview strategy. After the think-aloud interview, participants were guided into an online meeting room, where one researcher (KS) moderated the 35-minute group discussion structured by the guiding questions (Multimedia Appendix 1). Other researchers took field notes and asked follow-up questions at the end of the discussion but did not intervene. Guiding questions were based on prior national research on maturity model development [35], and refined in consultation with project members. A 5-minute exit phase concluded data collection, during which participants were informed about the availability of results and invited to the final study event. Afterward, researchers held a 15-minute debriefing to discuss key impressions and themes from their field notes.

### Qualitative Content Analysis

Applying a deductive qualitative content analysis approach [36], coding was done by 5 members of the study team (KS, JA, LB, AN, and JP) using a coding form developed in Microsoft Excel for this purpose. Deductive content analysis allows for retesting existing data in a new context, involving testing of models [36]. Units of analysis are the participants’ verbal contributions during the think-aloud interviews and focus group discussion. Audio files recorded during the think-aloud interview were randomly assigned to one coder, who listened to the recording, transcribed anchor statements, and paraphrased them. A second coder (KS) reviewed all transcripts and codes to validate the first coder’s analysis. The structured categorization matrix [36] consisted of all dimensions and attributes of the AINCRA pilot version. For each interviewed person and AINCRA dimension, attribute, and level description, statements were documented as anchor statements. The same was done for statements on the relevance of attributes and suggestions made for revision or changes to AINCRA. The coding framework for the focus group discussion followed the guiding questions, with the overall coding process being the same as for the think-aloud interviews, using an unconstrained categorization matrix, where various categories were formed within the deductive framework based on the principles of inductive content analysis [36].

### Mixing of Results and Consensus-Building on Final AINCRA Version, Translation Strategy

Overall, our integration strategy consisted of the following steps: deriving AI readiness attributes and dimensions from prior work and the results of the systematic review. Building on this, an initial AINCRA version was developed, including the definition of attributes for discussion with experts. The results of these discussions were taken into account in the development of the final AINCRA version: in a half-day consensus-building workshop, results from the think-aloud interviews and focus group discussions were reviewed by 6 members of the study team and discussed one last time against overlapping or contrasting themes derived from the systematic literature search. Further, consensus on which changes suggested by the experts should be incorporated into the final AINCRA version was established by discussion, and the final AINCRA version and a user manual were developed in German and English language. As the qualitative data collection was conducted in the German language, results were first compiled in German and then translated into English for publication. Primary translation was performed by the first author (KS) who was the main researcher involved in data collection and data analysis. All members of the study team had access to the German-language results and validated and revised the translated results, with multiple researchers having extensive experience in English-language knowledge transfer and dissemination of research results (DF, FB, KWO, MS, JP).

## Results

### Overview

Multimedia Appendix 2 presents the final AINCRA and Multimedia Appendix 3 contains the AINCRA user manual. An open-access online version of AINCRA is available [37]. In the following, we outline the iterative research process to contextualize the results contributing to the final AINCRA, before presenting an overview of AINCRA and its recommended application.

### Preliminary Database

First, we built on preliminary work from our working group which has been published elsewhere: data from a rapid review [3] including 292 publications on AI systems in nursing care, an expert workshop with 21 experts (including nurses, nursing directors, health care managers from hospitals, nursing homes, and home care services, digitalization officers and professionals with experience in digital health or routine nursing data analysis, informal caregivers, nursing scientists, and researchers in nursing education, computer science, AI, and ethics) as well as interviews with 14 experts and an online survey (n=53) were used to identify success factors and prerequisites of AINC projects [13]. Five initial key dimensions (namely, regulatory requirements, processual and translational requirements and aspects, technical requirements, social and ethical aspects, and community building) entailing 35 codes emerged as a result of qualitative structuring content analysis involving deductive as well as inductive category and code building.

### Systematic Literature Review on AI Readiness Models in Health Care and Nursing

**Figure 2.**
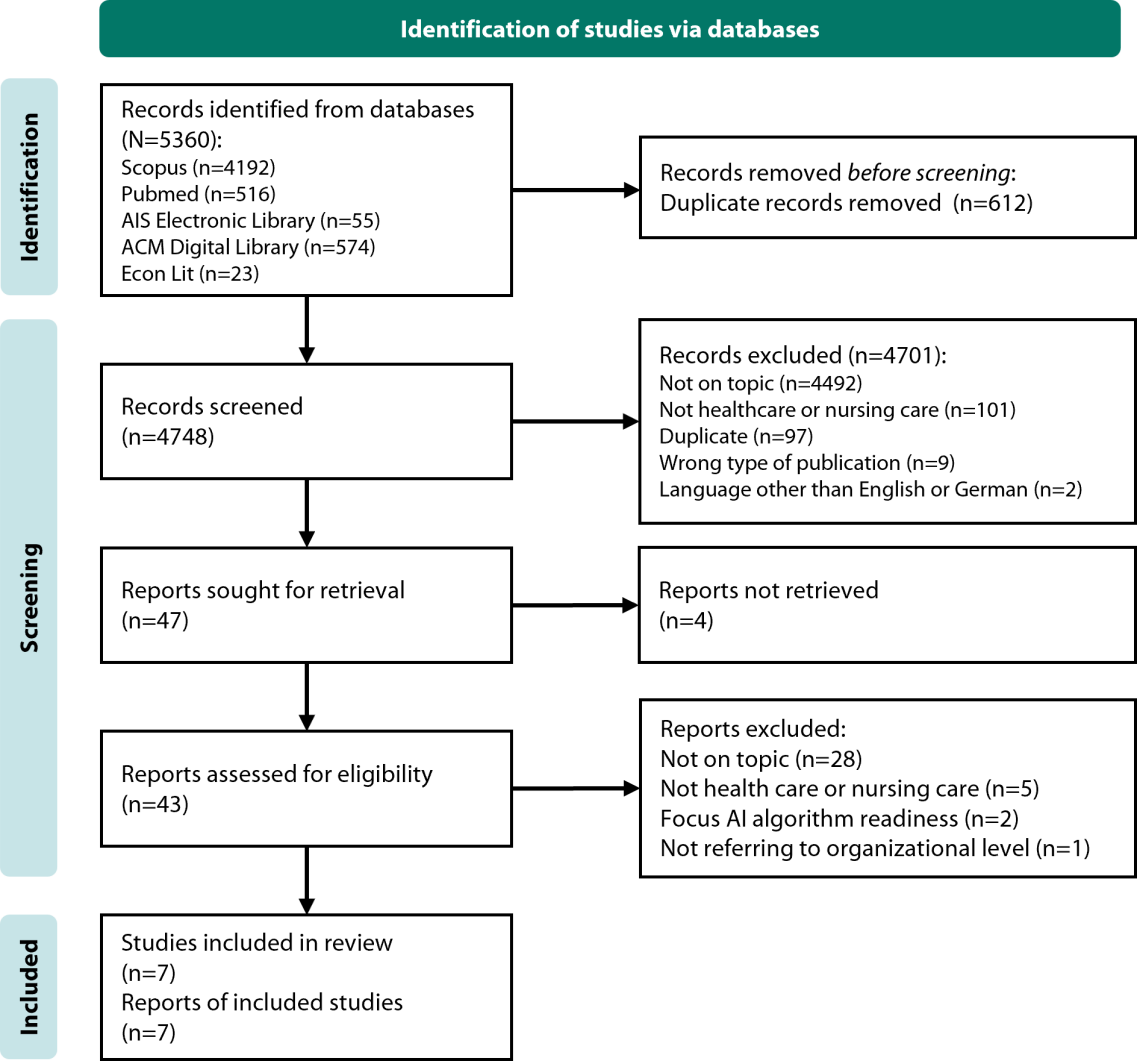
PRISMA (Preferred Reporting Items for Systematic Reviews and Meta-Analyses) flow diagram of identification of studies via databases. AI: artificial intelligence.

Second, we conducted a systematic literature review to identify existing AI readiness models focusing on nursing or health care settings and harmonize AI readiness capabilities, attributes, and dimensions with results from the first step. Screening of 4748 records identified from 5 leading health and information sciences databases resulted in the inclusion of 7 single publications (Figure 2) which reported either at least one question or evaluation criterion of AI readiness with a qualitative assessment option or a framework for theoretically or empirically defining AI readiness in health care or nursing care.

Table 1 provides an overview of the included studies and reports, 3 of which used observational designs with cross-sectional surveys among health care and IT professionals [38-40]. One study applied a qualitative design [25], 1 paper was classified as an expert reflection [41], and 2 papers did not report specific study designs to derive their results from [30,42]. Health care settings focused on either hospitals or selected professional groups or remained unspecified, without referencing specific nursing care settings in acute or long-term care. Overall, we extracted 74 single AI readiness attributes, which we inductively grouped into 47 codes in 13 AI readiness dimensions depicted in Table 2.

**Table 1.** Overview of included studies (N=7).

Reference; country of origin	Design	Aim	Population (N)	Reporting of				Number of readiness attributes
				Definition of AI^a^	Definition of AI readiness, explicit perspective	Readiness assessment description	Origin of readiness attributes	
Abuzaid et al 2022 [38]; United Arab Emirates	Observational, cross-sectional survey	Investigate knowledge, perception, readiness, and challenges regarding AI integration into radiology practice	Radiology professionals (n=153)	Y^b^	N^c^ Organizational readiness	N	N	3
Alami et al 2020 [41]; International	Expert reflection	Bring forward the importance of studying organizational readiness to integrate AI applications	Not applicable, experiences with AI systems to support clinical decision-making reported	Y	Y Organizational readiness	Y Adapted framework from Jennett et al [43]	Y	Not applicable, readiness dimensions reported
Andersson et al 2021 [39]; Sweden	Observational, cross-sectional survey	Establish a foundation for a Swedish perspective on the potential effect of AI on the medical physics profession	Medical physicists (n=163)	N	N Workplace preparedness	N	Y	2
Chang 2020 [30]; country not reported	Unclear	Not reported	Not applicable	N	N	Y	N	10
Pumplun et al 2021 [25]; Germany and Switzerland	Qualitative	Explore factors that influence the adoption process of ML^d^ systems for medical diagnostics in clinics; demonstrate how factors can be used to determine the ML maturity score of clinics	Medical experts and suppliers with profound knowledge in the field of ML (n=22)	Y	N	Y Maturity Model for ML Systems in Clinics	Y	12
Weinert et al 2022 [40]; Germany	Observational, cross-sectional survey	Investigate factors influencing AI readiness as well as possible barriers to AI adoption and implementation in hospitals; assessed the status quo regarding the dissemination of AI tools in hospitals	Hospital chief information officers (n=40)	Y	Y Organizational readiness	Y Model by Jöhnk et al [21]	Y	47
Wiljer and Hakim 2019 [42]; Canada	Unclear	Not reported	Not applicable	Y	N	Y AI-enabled organization	N	Not applicable, readiness dimensions reported

a AI: artificial intelligence.

b Y: yes.

c N: no.

d ML: machine learning.

**Table 2.** Dimensions of AI^a^ readiness derived from the literature (thematic, nonhierarchical grouping).

AI readiness dimension	Number of codes	Derived from
Personnel resources and competencies	12	[25,30,38-42]
Strategic planning	7	[25,30,38,40-42]
Data quality	8	[25,30,40,41]
Financial resources and investments	4	[25,30,40,41]
Acceptance and stakeholder participation	3	[30,39,41]
Technical infrastructure	3	[25,30,40]
Data protection and data safety	2	[30,41]
Intangible assets	2	[30,40]
Leadership culture	2	[25,30]
Needs and problems (in clinical practice and of patients)	1	[41]
Practical benefit and added value	1	[41]
Time resources	1	[40]
Organizational culture	1	[30]

a AI: artificial intelligence.

Axial coding and contrasting of these 74 readiness attributes with the 35 codes extracted in step 1 provided the database for 2 rounds of nominal group consensus involving 5 experts (2 nursing scientists, 1 ML expert, 1 information systems specialist, and 1 expert representing the German association for digitalization in the social economy with experience in consulting of clinical partners) to harmonize and group AI readiness factors. By merging semantically equivalent attributes and removing those not directly influenced by AINC stakeholders, the AI readiness attributes were reduced to 67 codes in 5 dimensions, serving as the baseline for the development of a pilot AINCRA version.

### Indicator Development and Expert Validation

The pilot AINCRA version included feedback from 13 AINC project experts to establish a common AI readiness vocabulary for level distinctions. Building on vocabularies of existing maturity or readiness models [23,44], expert feedback was obtained from researchers and practitioners working in ongoing AINC projects at the time of data collection during a 45-minute bar camp session in September 2024 which was open for participation for all people currently used in 1 of 9 AINC projects in the German Ministry for Research, Technology and Space’s funding program “Making Repositories and AI Systems Usable in Everyday Care.” Further, level labels, frequencies, and numbering reported in the literature [23,25] were discussed with these experts, resulting in the decision to adopt 5 levels (1: initial, 2: assessing, 3: determined, 4: managed, and 5: optimized) with the possibility to rate an attribute as not applicable depending on the type of AINC project, AI system, or organizational features of clinical partners. The complete AI readiness vocabulary for level distinction is included in Multimedia Appendix 3. The use of the vocabulary and the 67 attributes derived from the third step of the research process as input for GPT-4o (OpenAI)–generated 335 level descriptions, which, after expert review and revision by all members of the study team, were incorporated into the AINCRA pilot version.

Finally, to evaluate the AINCRA pilot version, we applied a think-aloud interview approach [45,46] including a concurrent think-aloud session and reflexive group discussion with 18 experts with a proven track record in carrying out AINC projects to refine AINCRA dimensions, attributes, level distinctions, and descriptions. The think-aloud approach can be useful to “better understand thought processes during assessments as a strategy to describe what [an] assessment is measuring” [46]. Further, the think-aloud approach provided information about the cognitive thought processes of AINC project stakeholders using AINCRA “pertaining to usability problems they would encounter” [47] to reflect on a specific AINC project. Considering possible ways to evaluate maturity models, we followed good-practice recommendations by involving experts “on the type of process that is intended to be improved by the maturity model, but who have not been involved in the actual development of the maturity model” [48].

### Characteristics of Think-Aloud Study Participants

At the time of data collection, 10 experts are involved in AINC projects outside of the German funding program “Making Repositories and AI Systems Usable in Everyday Care,” while 8 experts are working in ongoing AINC projects in said funding program (characteristics of participants are depicted in Multimedia Appendix 1). Eight experts report less than 5 years of experience with AINC projects, while 5 experts indicate experience of 5 years or longer. These 5, as well as 5 experts who did not report conclusively on the duration of their experience, had also been involved in projects focusing on developing and implementing other digital technologies in nursing care, digitalization, and advancement of IT infrastructures in hospitals and long-term care for several years. Considering the main AINC project stakeholder groups regarding the composition of project consortia, 9 experts represent a nursing or health science perspective, often accompanied by responsibilities and roles connected to project coordination, while in some cases also holding topic-related qualifications or work experience such as nursing informatics expertise. Five experts represent clinical partners in AINC projects, and 5 experts represent AI R&D, 2 of them working for tech companies compared to 3 experts working for institutes affiliated with universities or independent research institutions. In the think-aloud interview, 13 experts reflect on an ongoing AINC project, 4 experts reflect on a completed project, and 1 expert reflects on a project in planning.

### Think-Aloud Interview and Expert Group Discussion

Individual 30-minute think-aloud interviews and subsequent 35-minute group discussions were conducted online by 1 to 3 members of the study team in an overall 90-minute session. All participants received an overview of all AINCRA pilot version dimensions and attributes in advance. However, detailed level descriptions were only presented during the interview, along with the verbal prompt to evaluate an ongoing, in planning, or past AINC project they were familiar with. They were asked to do so by using the attributes and level descriptions of one or more selected AINCRA dimensions, referring to a recalled point in time (eg, planning, implementation, or evaluation phase), while verbalizing all their thoughts aloud. Since we anticipated the pilot version to be a complex and time-consuming assessment, participants were only presented with selected dimensions, never all of them. The selection was made with knowledge of the participants’ areas of expertise, with a slight emphasis on process-related requirements. This focus was chosen because these dimensions included attributes that are described in the literature as being often neglected in AI maturity models [20], and which, according to our preliminary study, are known to significantly influence project flow and success [13].

We obtained expert statements and comments on 64 of 67 attributes with a total of 70 suggestions for changes to the AINCRA pilot version (Multimedia Appendix 1). Changes suggested included adding definitions, or explanations, or examples for selected terms (eg, “data champion” and “ontological representation”), simplification of terms, reordering and grouping of attributes within 1 dimension or across 2 dimensions, splitting of attributes, and adding of examples in level descriptions (eg, sponsor role as a required responsibility when compliance with the EU Medical Device Regulation is mandatory for the AI system). Some comments highlighted the need for adding information to the AINCRA user manual without resulting in changes to dimensions, attributes, or level descriptions. Overall, 37 of 70 (53%) changes suggested were realized in the final AINCRA version.

In the group discussions, experts rated and discussed the AINCRA dimensions, attributes, and level descriptions they had applied to their own AINC projects in the think-aloud interview. An overview of the discussed evaluation criteria with their respective guiding questions for the discussion can be found in the “Methods” section. In summary, AINCRA was assessed as a valuable tool for AINC project development, management, and evaluation by the experts. However, some experts pointed out that not all influencing aspects of AINC projects can be foreseen using AINCRA, and that a project process can also be set up without applying AINCRA. Further, general aspects of good practice of project or data management that still need to be considered in each project should not be overlooked when focusing on AINCRA as a project management tool. Multimedia Appendix 1 displays expert ratings and feedback for each evaluation criteria. Results for each evaluation criteria are summarized below and highlighted with selected expert statements.

### Benefits and Consequences of Applying AINCRA

Considering benefits and consequences of applying the AINCRA to AINC projects, experts generally see added value. Advantages of applying the complex AINCRA are seen by some as being more suited to larger research projects rather than smaller initiatives or in-house projects, while others also recognize its value for those smaller endeavors. Added value was also noted for project consortia with little prior experience in conducting AINC projects. For clinical partners, AI R&D, and nursing or health science research partners in AINC projects, AINCRA is seen as useful for decision-making, planning, grant applications, coordination among partners, identifying resources and expertise, aligning goals, managing projects, and conducting evaluations. Experts also pointed out AINCRA’s usefulness for AINC project funding bodies, as AINCRA may support documentation, monitoring, evaluation, and comparison of AINC projects. Expert participants representing tech companies pointed out that AINCRA may help with their project management activities, client consulting, and long-term support of AI implementations.

*I think another advantage is that with [AINCRA], you can already identify project risks [...] during project planning, for example the area of social and ethical success factors [...] which is very helpful if such a dimension is already represented here.*

[IP2, 00:48:18]

Results of AINCRA may lead to various consequences for AINC projects: positive consequences entail advantages in grant applications for high-scoring applicants, identification of improvement areas and necessary changes, support for go or no-go AINC project decisions, and early reflection on and evaluation of an AI system’s practical value. However, an AINCRA result may evoke emotional effects in the individuals conducting the assessment, influencing their motivation to engage with AINC readiness attributes or to push an AINC project forward. Low AINCRA scores may cause frustration or disappointment but can also spark motivation and ambition to improve. However, low-level placement may demotivate clinical partners or discourage participation if initial AINC readiness appears too low and difficult to improve.

*Especially if the requirements are not met at the beginning, it can quickly give the impression that one doesn’t want to do it and doesn’t see the benefit for the effort that has to be put in, even though there actually is one.*

[IP14, 00:54:16]

### Rater Entity

When asked who should carry out the assessment, experts agree that AINCRA is unlikely to be effectively carried out by a single individual. Depending on the dimension and attribute, different individuals should be involved, or AINCRA should be conducted collaboratively, either in a group or in a tandem (eg, between clinical and AI R&D partner) within an interprofessional team. For assessing processual and translational requirements and aspects, suitable raters include experienced personnel from the clinical partner with nursing and digitalization expertise, and IT staff of the clinical partner in collaboration with nursing management with a focus on joint assessments by AI R&D and clinical partners. Conducting AINCRA as a guided, participatory assessment involving frontline nurses was stated as a suitable approach. However, some experts caution against including certain roles (eg, nursing assistants) and emphasize that nurses should be supported during their involvement. Experts also raise the question of who holds responsibility for the AINCRA result, especially if the result impacts, for example, project funding decisions.

*I think it would be good if the evaluation is done at the start by the project consortium, not by the individual specialists [...] it would be good if everyone in the consortium, with its different disciplines, is clear about [...] all five dimensions [...] so that together they have a clearer idea of what lies ahead.*

[IP3, 00:52:05]

### Attainability of Levels

Assessing the attainability of AINCRA levels, overall, experts view the levels as realistic and reflective of the diversity among clinical partners and AINC projects. However, not all levels appear to be achievable. They emphasize that this should not necessarily be the expectation for AINC projects, as attainability of levels depends on the dimension assessed and the project context. Level 5 is ambitious and currently hard to reach for most clinical partners. But experts emphasize that level 5 should be a long-term goal and is seen as achievable, especially for clinical partners with extensive AINC project experience, but is considered unrealistic for R&D projects. Level 4 is regarded as the upper realistic limit for R&D projects and a desirable target for clinical partners. Level 3 is also seen as a satisfactory goal for clinical partners. While level 2 was not discussed in detail, for level 1, experts point out that some clinical partners already start AINC projects while scoring above level 1.

*I would definitely say that we have developed. There were some levels that, from our perspective, were not achievable. But the question is also whether that’s the goal. Maybe it’s enough to say that you get through with a 3.*

[IP11, 00:51:18]

### Achievability of Levels

Reflecting on past AINC projects that, from the experts’ point of view, were successful, experts discussed the achievement of the AINCRA levels regarding the level at the start of a project and the evolution of levels throughout the project process until completion. Overall, the achievement of AINCRA levels is rated as depending on individual goals and structures of an AINC project. Progression is inherent to AINC projects, as no project meets all requirements from the start. Participation in an AINC project can lead to the development of new structures and processes at the clinical partner organization that go beyond AINCRA attributes. In contrast, some AINC projects may not show improvement despite great efforts to advance AINC readiness attributes. All types of development trajectories are possible, with advancement, remaining, or regression of levels. Entry levels and basic conditions may help industry (or R&D) partners to more easily identify suitable clinical partners, with level 1 being rated not as a general exclusion criterion, but a knockout criterion for certain attributes (eg, data availability).

*I do believe that there can be deal-breaker criteria in attributes. Off the top of my head, I thought of data quality and data availability in our project. Especially structural things – if those aren’t in place, we don’t need to start an AI project. If my data are just scattered on paper.*

[IP13, 00:53:24]

### Step Size of Levels and Influenceability of Attributes

Step size of AINCRA levels is rated as sufficient, appropriate, and distinct with little in-depth discussion. Influenceability of the AINCRA attributes was discussed in depth: most attributes are considered influenceable within AINC projects. However, some less influenceable attributes are still important, such as regulatory requirements or legal framework changes, which are typically beyond a project’s control. Experts highlight influenceability as a reflection criterion, especially when a project receives a low maturity level rating. Attributes considered particularly influenceable are related to attitudes, acceptance, knowledge, and willingness to change of stakeholders at the clinical partners’ organization. Limited influenceability is assigned to attributes requiring fundamental corporate decisions (eg, developing and committing to an internal AI strategy) as well as attributes dependent on external conditions of clinical partners (eg, staff shortages, turnover, and financial resources).

*[AINCRA attributes are] all topics that I can address within the project.*

[IP11, 00:55:39]

### Availability of Information, Comprehensibility, and Applicability

Discussion of the availability of the information or data needed to carry out the AINCRA revealed that the required information is rated as available or obtainable with reasonable effort for AINC project stakeholders by the experts. Involving additional stakeholders is emphasized, supporting the previous assessment that AINCRA should be conducted as an interprofessional assessment. However, the availability of additional stakeholders may be limited, for example, if IT staff of clinical partners have competing responsibilities and carrying out AINCRA is considered a lower priority compared to other tasks.

*All [attributes] I have seen so far [.] are definitely such that you can either answer them yourself or at least know who to turn to in order to get the information.*

[IP12, 01:14:26]

Regarding the comprehensibility of the AINCRA, some experts consider the instrument to be low-threshold and emphasize its comprehensibility for AINC project leaders. However, some wording is not familiar to all nursing professionals. There is also concern about whether nonnative speakers in nursing leadership or administrative roles will understand the expressions and terminology used in AINCRA. Referring to statements made in the think-aloud interview, experts provide suggestions for logically ordering, rephrasing, or explaining selected attributes, as details of the attributes and level descriptions were rated as not quickly to be grasped.

*So, I would assess it as very understandable [...] of course, during the [think aloud interview], I naturally picked out the things I understand in order to be able to answer them [...] the comprehensibility depends on who from the consortium is conducting the assessment [...] regarding nursing topics, the IT partners probably would have said, ‘I don’t understand anything about that at all’ [...] it depends on who is sitting in front of it and which professional domain it is.*

[IP2, 01:03:33]

For the applicability of AINCRA, experts assess the AINCRA pilot version as a complex, text-heavy instrument that, when applied thoroughly, can take several hours to complete depending on the dimension rated. This long application duration may reduce acceptance of AINCRA. Initial use in particular requires time to understand the instrument and is not necessarily intuitive. However, experts expect that the time required will decrease over time and that the application will become easier with continued use. Conducting AINCRA in an interprofessional team enhances its applicability. Some experts attest to the very good usability of AINCRA, though it should be noted that no expert applied the entire instrument. Application seems challenging for stakeholders in long-term care facilities due to lack of expertise in certain attributes. Experts discussed whether a shortened AINCRA version could be provided.

### Comparability and Completeness

Comparability of AINCRA results between AINC projects is assessed heterogeneously between the experts, with some pointing out that comparability is difficult, and others emphasizing that comparability of individual dimensions and attributes is possible. Further, multiple practice partners within one AINC project may be compared with AINCRA, as well as multiple AINC projects within the same funding program, and comparability can be ensured through external evaluation.

*Of course, it’s a self-assessment tool [...] the question is whether it’s answered honestly and whether that allows for comparability. If it’s evaluated in a review process by external parties, then I do believe that good comparability can be achieved.*

[IP8, 00:21:20]

Completeness of AINCRA overall is rated as given, with some experts highlighting suggestions for additions or change. These address the inclusion of staff representatives of clinical partners as important stakeholders, allowing for custom attributes to be added by users, and enabling mapping of multiple clinical partners within a single AINC project.

### Overview of AINCRA Dimensions, Attributes, and Levels

AINCRA consists of 69 AINC readiness attributes across five dimensions: (1) regulatory requirements and aspects (9 attributes), (2) processual and translational requirements and aspects (40 attributes), (3) technical requirements and aspects (6 attributes), (4) ethical and social requirements and aspects (11 attributes), and (5) community building requirements and aspects (3 attributes). Table 3 provides an overview of the 5 dimensions. For each attribute, 5 maturity levels are described, a recommendation for stakeholders considered as most suitable as assessors is given, along with a reference to the source from which the attribute or the justification for the relevance of the attribute is derived (Multimedia Appendix 2).

**Table 3.** Overview of AINCRA^a^ dimensions.

Dimension	Description
Regulatory requirements and aspects (9 attributes)	This dimension takes into account the necessary legal frameworks and data protection regulations that require an analysis of the data and an examination of data-sharing models to enable the use of AI^b^ systems in everyday nursing practice.
Processual and translational requirements and aspects (40 attributes)	This dimension captures the human, material, temporal, and intangible resources available for AI in nursing care projects in care facilities or hospitals, as well as their general and AI-specific level of digitalization. It also assesses the maturity of overarching strategies for AI, data governance, and IT governance, as well as plans for long-term external support and evaluation of AI implementation. Engagement with the implementation of needs-based research and development of AI systems—focused on practical benefits and added value—as well as the existing AI-related knowledge and competencies within care facilities and clinics, is intended to support the seamless integration of AI systems into existing care processes. This dimension also includes reflection on attitudes toward AI, acceptance of AI technologies, and trust in their use in daily nursing practice, to foster adoption and address concerns of those affected by the deployment of AI.
Technical requirements and aspects (6 attributes)	This dimension addresses the availability and functionality of the technical infrastructure, including aspects of data protection and data security, which are essential for reliable data use and analysis. To ensure smooth, secure, and efficient data integration and exchange between different systems and stakeholders in everyday nursing practice, this dimension also considers the integration of AI systems into data infrastructures or data platforms, as well as the use of technical interoperability standards and nomenclatures.
Social and ethical requirements and aspects (11 attributes)	This dimension encompasses ethical and methodological considerations related to voluntariness, privacy, fairness, and transparency, as well as the ethical and normative value orientations of the field and individual practice partners. Engaging with the impacts of AI use at the micro, meso, and macro levels, along with a responsible approach to data handling, is intended to support the use of AI systems in everyday nursing practice.
Community building requirements and aspects (3 attributes)	This dimension aims to promote a network that strengthens knowledge exchange and the collective advancement of AI systems in everyday nursing practice. Researchers, developers, and stakeholders from nursing practice and nursing management are involved in this network.

a AINCRA: artificial intelligence nursing care readiness assessment.

b AI: artificial intelligence.

AINCRA dimension 1, regulatory requirements and aspects, involves the assessment of how detailed and specific methodological and regulatory decisions, definitions, and limitations are being addressed by an AINC project. This includes, among other things, attributes reported by Pumplun et al [25] for analyzing the dataset, as well as the operationalization of data-sharing models [30,41,49]. In the latter case, for example, the degree of finalization and the progress of the implementation of the model serve as reflection criteria. This dimension also relates to the engagement with requirements arising from the EU AI Act. AINC project stakeholders recommended for assessing dimension 1 are mainly individuals representing AI R&D, supplemented by a clinical partners’ and nursing science perspective for some attributes. An example of the attributes 1.6 Data-sharing Models and 1.9 EU AI Act*,* corresponding level descriptions, recommended rater entity, and sources of attribute origin is shown in Table 4.

AINCRA dimension 2, processual and translational aspects and requirements, evaluates the resources, general as well as AI-specific digital readiness, and strategic planning in place for implementing AINC. It considers infrastructure, staff skills, governance, and external support, as well as how well AI aligns with care needs and existing workflows. It also examines staff attitudes, acceptance, and trust in AI to support successful integration into everyday practice. Most attributes are targeted at the clinical partner, whose expertise is crucial for assessing this dimension and which needs to be supplemented by nursing science and AI R&D expertise to arrive at a comprehensive assessment. This is reflected, for example, in attributes targeting the degree of digitization, which includes the use of data quality standards [25], the availability of data champions [30] as a personnel resource, or the systematic demonstration of practical benefit and added value of an AI system (Table 4).

AINCRA dimension 3, technical requirements and aspects, focuses on the technical infrastructure needed for reliable and secure data use in AINC projects. It includes data protection, system integration, and the use of interoperability standards to ensure smooth data exchange and effective AI implementation in clinical practice. The main AINC project stakeholder to assess dimension 3 is AI R&D. While the technical infrastructure at clinical partner sites is an essential and priorly described prerequisite for AINC project success [30,41,49], we recommend especially assessing IT infrastructure with a focus on AI compute capabilities and dedicated hardware, for which requirements are often underestimated or not clearly communicated between AI R&D and clinical partners during initial AINC project planning (Table 4).

AINCRA dimension 4, ethical and social requirements and aspects, covers ethical and methodological aspects such as voluntariness, privacy, fairness, and transparency. It also considers the values of the nursing field and its practitioners, as well as the broader impacts of AI use and responsible data handling to support ethical AI integration in daily nursing care. Dimension 4 should be assessed from a multifaceted perspective, including nursing science and clinical partners. In collaboration with clinical partners and AI R&D, nursing scientists should drive the assessment of whether and how structured engagement with ethical-normative values of nursing and care [49] is operationalized in an AINC project. The same holds true for reflecting on the impact of AI on the nursing profession [49], and how this impact informs AI system development, implementation, and evaluation (Table 4).

**Table 4.** Examples of attributes and level descriptions across the AINCRA^a^ dimensions.

Dimension and attribute number	Attribute	Level 1 (initial)	Level 2 (assessing)	Level 3 (determined)	Level 4 (managed)	Level 5 (optimized)	Not applicable	(Joint) Assessment by (rater entity)	Source
Regulatory requirements and aspects									
1.6	Data-sharing models	Data-sharing models relevant to the AINC^b^ project are unknown or unclear. No considerations regarding appropriate models and approaches exist.	Initial considerations and steps toward defining and implementing a data-sharing model are being taken. However, the model is not finalized, and necessary people and procedures are not yet fully clarified.	The data-sharing model and approach are defined, and most legal, organizational, and technical requirements are identified. Contact persons and responsibilities are known, but detailed negotiation and implementation have not started.	The data-sharing model and approach are defined and known to all project participants. All legal, organizational, and technical requirements are fully identified and documented in a structured manner. Responsible contacts are involved and took part in detailed negotiations. Implementation includes appropriate regulatory, organizational, and technical safeguards	The model and processes of data sharing have been implemented with all necessary regulatory, organizational, and technical safeguards. Fulfillment of requirements is proven (eg, through internal and external certification). Data sharing and usage are fully documented, and documentation is regularly reviewed. Processes and structures evolve based on audit results.	Not applicable	AI^c^ R&D^d^	[30,41,49]
1.9	EU^e^ AI Act	No consideration of the EU AI Act in the AINC project. No awareness of which parts of the project are affected.	Initial steps to comply with the EU AI Act are underway. A basic overview exists, but the requirements are inconsistently implemented. Responsibilities and external actors are not fully clarified.	The AINC project is committed to compliance with the EU AI Act. A broad overview exists, responsibilities are defined. Many requirements are implemented or planned, but coverage and documentation are not complete.	The AINC project is committed to AI Act compliance and has a structured overview of legal requirements. Responsibilities are clear and implementation follows a defined process standard. All requirements are planned or fulfilled with explicit deadlines and sufficient resources (time, personnel, and other). Implementation is well-documented, tested, and regularly monitored.	Full compliance with the EU AI Act is ensured. A complete and structured legal framework exists and is shared with all participants. Responsibilities are clear, and implementation follows international standards (eg, ISO/IEC 42001). All requirements are planned or fulfilled with explicit deadlines and sufficient resources (time, personnel, other), documented, tested, externally certified, and continuously improved.	Not applicable	AI R&D Nursing Science Clinical partner	Emerged from this research
Processual and translational aspects and requirements									
2.9	Clinical partner: personnel resources: available data champions^f^	There are no data champions (at the clinical partner) in the AINC project, and the use of data is not actively promoted or supported.	First data champions (at the clinical partner) are being identified, but their role is not yet formalized or recognized.	Some data champions (at the clinical partner) are active, their role is defined, and they promote a data culture in selected areas.	Data champions (at the clinical partner) are established and work across departments to promote and support data use.	Data champions are fully integrated into the organizational structure (of the clinical partner) and actively drive a data-driven culture forward.	Not applicable	Clinical partner with IT staff AI R&D	[30]
2.32	Practical benefit and added value of the AI system	The practical benefit or added value of the AI system is unknown, or no clear practical benefit or added value is evident. The AINC project is predominantly theoretical.	The potential benefit of the AI system is recognized, but concrete indicators or criteria for capturing the benefit and added value are unclear. Initial steps to evaluate the added value are being taken but are not consistently recorded and documented.	The AI system shows a recognizable practical benefit, which is represented by concrete indicators or criteria. However, end points are only partially captured, or the benefit is only partially realized.	The practical benefit of the AI system is clearly defined and is systematically implemented and demonstrated within the AINC project.	The AI system generates significant practical benefit and added value, which is continuously measured and optimized using clearly defined criteria and indicators.	Not applicable	AI R&D Nursing Science Clinical Partner	[30,41]
Technical requirements and aspects									
3.2	Use of technical interoperability standards and nomenclatures	No use of interoperability standards or nomenclatures.	Initial steps toward using and considering interoperability standards and nomenclatures in the development of the AI system within the AINC project, but not yet fully implemented.	Interoperability standards and nomenclatures are partially considered and used in the development of the AI system within the AINC project, but not consistently.	Extensive use and integration of interoperability standards and nomenclatures in the development of the AI system within the AINC project.	Complete and optimized use of interoperability standards and nomenclatures in the development of the AI system within the AINC project, regularly updated and applied across all relevant areas.	Not applicable	AI R&D Nursing Science	[30,41,49]
3.6	IT infrastructure: AI compute: hardware	No dedicated AI hardware available.	Dedicated AI hardware is available but not yet connected, eg, to data interfaces.	Dedicated AI hardware is available and connected (eg, to data interfaces). It is possible to transfer data into the runtime environment for model development.	Dedicated AI hardware is available and connected. Standardized interfaces are in place for data transfer and for offering model predictions. Continuous testing and maintenance of the AI system is possible.	Fully optimized and up-to-date dedicated (hardware and software) runtime environments are available. Model development and maintenance are implemented according to modern CI or CD components.	Not applicable	AI R&D	Emerged from this research
Social and ethical requirements and aspects									
4.4	Engagement with ethical-normative values of nursing and care, and individual clinical partners	No engagement with ethical values (has taken place) in the AINC project.	Initial ideas on how to engage with ethical-normative values in the AINC project exist but are not concrete or systematic.	Methods to engage with ethical-normative values are defined but are only partially implemented or exclude some stakeholders.	Regular, systematic, and documented engagement with ethical-normative values including all stakeholders is part of the AINC project.	Regular, systematic, and documented engagement with ethical-normative values including all stakeholders is part of the AINC project, and is adapted as needed.	Not applicable	AI R&D Nursing Science Clinical Partner	Emerged from our prior research [49]
4.6	Reflection on the impact of AI on the nursing profession	The general influence of AI on nursing is acknowledged, but there is no project-level reflection.	Initial reflections on the impact of AI on the nursing profession are present. However, reflections are unsystematic and without an effect on system development, implementation, or evaluation.	Systematic reflection on the impact of AI on the nursing profession is part of the AINC project but lacks influence on development, implementation, or evaluation.	Comprehensive reflection on the impact of AI on the nursing profession that informs system development, implementation, and evaluation is part of the AINC project.	Fully integrated reflection on the impact of AI on the nursing profession is included in the AINC project results and documentation.	Not applicable	AI R&D Nursing Science Clinical Partner	Emerged from our prior research [49]
Community building requirements and aspects									
5.3	Strategic partnerships	Existing strategic partnerships are unknown or there are no strategic partnerships in the AINC project or among individual project partners.	Initial strategic partnerships are being explored, but have not yet been fully concluded or formalized.	Strategic partnerships are partially established, but not all partners in the AINC project have strategic partnerships. Clinical partners in particular do not have strategic partnerships outside of the project consortium.	Comprehensive strategic partnerships are established. Clinical partners also have strategic partnerships outside of the project consortium. Existing strategic partnerships are regularly reviewed and expanded, especially on a national level.	Proven and effective strategic partnerships are established for all partners in the AINC project. Existing strategic partnerships are continuously and internationally expanded.	Not applicable	AI R&D Nursing Science Clinical partner	[42]

a AINCRA: artificial intelligence nursing care readiness assessment.

b AINC: artificial intelligence in nursing care.

c AI: artificial intelligence.

d R&D: research and development.

e EU: European Union.

f Data Champions understand the types of data generated at the clinical partner, advocate for proper data handling, and mediate between nursing staff and IT. They ensure data are correct, complete, and up to date, identify and resolve data collection issues, enhance staff data literacy through training, and raise awareness about the importance of data in nursing care and research.

AINCRA dimension 5, community building requirements and aspects, promotes a collaborative network to support knowledge exchange and the shared development of AI systems for nursing care, involving researchers, developers, and professionals from nursing practice and management, ideally reaching beyond single-project boundaries and striving for national and international networks and shared knowledge. All AINC project stakeholders can assess dimension 5 with AI R&D and nursing science, especially reflecting on their involvement in technical knowledge transfer [49], while clinical partners should also reflect on existing strategic partnerships and how to foster these [42] (Table 4).

### Applying AINCRA

AINCRA is primarily designed as a self-assessment tool for AINC project partners. It addresses topics such as the use and benefit of AINC, data representativeness and sharing, participatory design, and ethical and professional implications of AI implementation. AINCRA can be used at any project stage: a single assessment provides a snapshot of current AI readiness, while repeated assessments enable monitoring progress over time. It may also serve as an external evaluation tool. In all cases, AINCRA should be applied by an interprofessional team, ideally involving representatives from AI R&D, nursing science, and clinical partner organizations. The tool can be used for an entire AINC project or for selected dimensions or sub-areas.

### Interpreting AINCRA

In the absence of an internationally agreed consensus on which factors most strongly determine the success of AINC projects, AINCRA does not produce an overall score. Instead, it prompts users to reflect systematically on all dimensions and attributes, which are consistently considered important for project success. AINCRA results provide a snapshot of an AINC project’s current readiness with regard to known prerequisites for successful AI development and implementation in nursing care. The tool can be used in context analysis, project planning, and formative evaluation to identify areas for further development or prioritization, and in summative evaluation to reflect on factors that influenced project outcomes. Across all project phases, AINCRA supports alignment, justification, and documentation of strategies while fostering constructive, content-focused dialogue among project partners.

## Discussion

### Principal Findings

Despite AI’s high potential to support nursing care, many use cases in acute and long-term care are still underexplored [13], and R&D projects face multifaceted challenges when designing, implementing, or evaluating AI systems in everyday nursing care. In this study, we systematically developed dimensions, attributes, and levels of AI readiness based on published empirical evidence as well as insights from experts with practical experience in conducting AINC projects. Integrating findings from prior work, a systematic literature review and expert discussions directly shaped the development of AINCRA. Attributes and dimensions identified in earlier work provided an initial conceptual structure that was operationalized in a first AINCRA version. This version served as the basis for think-aloud interviews and discussions with experts during which the relevance, clarity, and completeness of individual attributes were critically examined. Feedback from these discussions led to the refinement and consolidation of attributes and informed adjustments. As a result, the final AINCRA reflects a synthesis of theoretical grounding, empirical evidence, and practical expert insight. The resulting AINCRA comprises 5 dimensions and 69 AI readiness attributes across 5 readiness levels that can guide AINC project planning, implementation, and evaluation. While experts with practical experience in conducting AINC projects rated the readiness level 5 (optimized) as ambitious and often unrealistic for AINC projects, they recognized AINCRA's value and overall benefit to support AINC projects, especially with respect to early identification and thus avoidance of common pitfalls of these projects.

A major strength of AINCRA is its comprehensive inclusion of evidence-based, previously established AI readiness factors, with a focus on processual and translational aspects. Prior research on a sociotechnical perspective for responsible AI maturity models has shown that organizations often focus on technical and business assessments when developing and deploying AI systems, while overlooking critical ethical, social, and human dimensions [20]. We argue that this holds true for AINC projects as well, so that they will benefit from recognizing and systematically addressing the importance of both technical and social aspects [20]. In addition, AINCRA sets itself apart from other freely available or commercial instruments, such as the analytics maturity assessment model promoted by the Healthcare Information and Management Systems Society through its consistent focus on the specific application context of nursing care. Many of the models existing before the AINCRA development have not been thoroughly reviewed by the scientific community. Therefore, they were not identified during the systematic literature review. AINCRA goes beyond preexisting models, offering a framework that is both more specialized and scientifically validated for use in AINC projects. As most attributes were derived from and may be transferred to a global context, we see overall generalizability of our results, even though AINCRA was developed within the European legal framework for AI in health care settings. While experiences of AINC experts stem from a German health care and research system perspective that may differ from other countries’ prerequisites and available resources, for example, for funding AINC project staff, technical infrastructure or knowledge transfer and dissemination activities, as well as structures, processes, and outcomes of nursing care, we believe that European-centric attributes can be easily adapted to other regional or national requirements.

### Implications for AI R&D, Nursing Science, and Clinical Partner Organizations

Given the widely reported issue that actors from health care and information science often do not share the same domain language in R&D projects and frequently work past one another [50], creating transparency and a common understanding of project aims and key concepts should be a shared goal among AINC project partners. AINCRA contributes to achieving this goal by raising awareness of relevant aspects. It also provides a foundation for decision-making at the project initiation phase, helping to determine which partners need to be involved, at what stages, and with what level of resource commitment. Additionally, it enables a better estimation of the risks and costs associated with AI development and deployment. By operationalizing and making the AINC project’s progress more visible across different domains and project partners, it fosters a shared basis for discussion and terminology, promoting more effective collaboration.

Leveraging the potential of AI in health and nursing care requires robust estimates of a variety of factors relevant for such an investment, including implementation time, associated costs, regulatory risks, and technology acceptance among potential users and patients [21,25,30]. These estimates call for a thorough understanding of the status quo of health care institutions with respect to a variety of dimensions as detailed in AINCRA. The structure provided with AINCRA can support strategic decisions on investments by providing reliable insights on the efforts required to implement AI technology in clinical practice. The attributes developed in this study can also help to identify gaps and future areas of improvement with regard to AI technology in nursing practice. In addition, the insights gained in expert interviews help to manage expectations in translating and disseminating AI research to nursing practice. It is evident that an assessment of AI readiness will be challenging or even impossible without transdisciplinary teams that combine expertise in technical, regulatory, and ethical questions with expertise in nursing care.

Although AINCRA is extensive and complex, by advocating for an interprofessional approach, it enables a comprehensive evaluation of AINC projects, due to its high level of detail, ensuring that no blind spots are overlooked. Furthermore, AINCRA is applicable to a wide range of projects, as the term AI is defined broadly, making it relevant across various areas within the field.

### Comparison With Design Recommendations and Common Criticism of Maturity Models

Eight requirements for designing maturity models [23] were applied throughout the AINCRA development process. Table 5 outlines how these requirements were addressed and implemented in this study. Above all, the comprehensive comparison with existing maturity models, alongside an iterative approach and the sequential expert evaluation of attribute and level descriptions, strengthens the presented research.

**Table 5.** Incorporation of design recommendations in the AINCRA^a^ development process.

Requirement (R) derived from [23]	Incorporation of requirement through...
R1: Comparison with existing maturity models	Systematic literature review Further development of own prior work Focus on AI^b^ readiness or maturity models in health or nursing care
R2: Iterative procedure	Research process with 3 iterations
R3: Evaluation	Application and rating of AINCRA pilot version through experts
R4: Multimethodological procedure	Sequential exploratory multimethods design
R5: Identification of problem relevance	Rating and confirmation of AINCRA’s relevance in expert group discussions
R6: Problem definition	Results of preliminary work [13]
R7: Targeted presentation of results	Feedback from the target group of intended users Use of discipline-specific terminology Development of a standard vocabulary for level distinctions together with the intended users
R8: Scientific documentation	Study documents Ethics application Mandatory reporting for funding body Open access publication of AINCRA

a AINCRA: artificial intelligence nursing care readiness assessment.

b AI: artificial intelligence.

Three main criticisms of maturity models [24] should be reflected on regarding AINCRA’s internal and external validity: (1) lack of a solid theoretical foundation without grounding elements such as maturity levels and dimensions in established academic literature [24], (2) lack of sufficient empirical support for the selection of dimensions or variables, and (3) lack of a clear definition of how maturity should be measured [24]. While we rigorously aimed to address the first 2 criticisms during development by review of the academic literature and iterative evaluation and consensus building with domain experts, a degree of ambiguity in the measurement of AINC readiness remains. This arises partly from the nature of AINCRA as a self-assessment instrument. On the other hand, it is worth noting that AINCRA provides qualitative but not quantitative criteria for assigning a maturity level. Hence, an answer to the question, for example, whether simply participating in one regional network is sufficient to meet the requirements for community building attributes, or whether involvement in at least 3 different networks is needed, remains open to interpretation. Users may assess individual attributes with varying degrees of realism and flexibility. Further operationalization is therefore strongly recommended.

### Limitations and Future Research

Our results have limitations that call for further research. First, experts commented on 66 of 69 readiness attributes only. Attributes 2.39 strategies for long-term external support: software and hardware: upgrades, 2.40 strategies for long-term external support: software and hardware: maintenance, and 2.29 financial resources and investments: exploring alternative financing models for including clinical partners remained without comments. Why these attributes were not addressed, even though they have been identified as relevant for AI [41], is not obvious. Most likely is the limited time span of the think-aloud task, which may have constrained responses. Toward the end of the task, experts focused particularly on attributes they found interesting or especially relevant. Besides, not all invited experts participated. The findings are based on the expertise of a subset of participants. Although key stakeholders were represented, it cannot be assumed that data saturation was fully achieved, as not all invited experts took part in the study, and data collection concluded after interviewing all experts who had agreed to participation.

Second, as research on organizational AI readiness has previously highlighted as a relevant limitation [21], while AINCRA was developed to systematize key attributes of AINC readiness, overlaps with readiness or frameworks for adopting and implementing other digital technologies can be recognized, such as stakeholder acceptance as a key attribute for technology adoption [51]. However, the project-specific perspective of AINCRA expands preexisting frameworks, which predominantly adopt an organizational perspective, thus providing AINC projects with a reflective tool and instrument to support project management.

Third, we decided against substantially shortening or prioritizing AINC readiness attributes, despite suggestions from some experts. This makes AINCRA more time-consuming and potentially less practical for everyday project work. However, this decision is informed by our experiences with multiple AINC projects over the past 5 years, which show that many projects underestimate their complexity and are affected by domain-specific blind spots that hinder collaboration. At the same time, our findings do not fully clarify how individual attributes interact or which should be addressed together when strengthening AINC readiness. While we applied an established method for maturity model evaluation that is being used in information science for decades [48], future research may strive for validating our results by applying AINCRA to real process improvement activities [48] in future AINC projects.

### Conclusion

In a field where many use cases remain underexplored and R&D projects often face substantial barriers, AINCRA offers structured guidance to enhance AI readiness for research, development, and implementation of AI projects in nursing care. AINCRA provides a balanced integration of technical, organizational, ethical, and social aspects of AI readiness, addressing a common shortcoming of existing maturity models that tend to prioritize technical or business considerations. Its nursing-specific focus further distinguishes it from other generic or commercially available assessment instruments, making it both context-sensitive and scientifically grounded. Overall, AINCRA offers both a practical tool for current projects and a foundation for future research and refinement in the evolving field of AI systems in nursing care.

## Supplementary material

10.2196/84148 Multimedia Appendix 1 Additional methods information and results.

10.2196/84148 Multimedia Appendix 2 Artificial intelligence nursing care readiness assessment full version.

10.2196/84148 Multimedia Appendix 3 Artificial intelligence nursing care readiness assessment user manual.

10.2196/84148 Checklist 1 PRISMA 2020 checklist.

10.2196/84148 Checklist 2 COREQ checklist.

## Abbreviations

AI: artificial intelligence
AINC: artificial intelligence in nursing care
AINCRA: artificial intelligence nursing care readiness assessment
COREQ: Consolidated Criteria for Reporting Qualitative Research
EU AI: European Union artificial intelligence
ML: machine learning
PRISMA: Preferred Reporting Items for Systematic Reviews and Meta-Analyses
R&D: research and development

## References

[1] Hassanein S, El Arab RA, Abdrbo A (2025). Artificial intelligence in nursing: an integrative review of clinical and operational impacts. Front Digit Health.

[2] O’Connor S, Yan Y, Thilo FJS, Felzmann H, Dowding D, Lee JJ (2023). Artificial intelligence in nursing and midwifery: a systematic review. J Clin Nurs.

[3] Seibert K, Domhoff D, Bruch D (2021). Application scenarios for artificial intelligence in nursing care: rapid review. J Med Internet Res.

[4] (2025). Approval of the content of the draft communication from the commission - commission guidelines on the definition of an artificial intelligence system established by regulation (EU) 2024/1689 (AI act). https://ec.europa.eu/newsroom/dae/redirection/document/118559.

[5] Raza MM, Venkatesh KP, Kvedar JC (2024). Generative AI and large language models in health care: pathways to implementation. NPJ Digit Med.

[6] Majjouti K, Priester V, Tapp-Herrenbrueck M (2025). Nursing-centered development of an AI-based decision support system in pressure ulcer and incontinence-associated dermatitis management - a mixed methods study. BMC Nurs.

[7] Kopanz J, Mader JK, Donsa K (2022). Digital algorithm-guided insulin therapy in home healthcare for elderly persons with type 2 diabetes: a proof-of-concept study. Front Clin Diabetes Healthc.

[8] Majjouti K, Tapp-Herrenbrueck M, Pinnekamp H, Yurish SY KIADEKU: identification of wound types with AI. https://sensorsportal.com/AIMH_2025/AIMH_2025_Proceedings.pdf.

[9] Alves J, Azevedo R, Marques A, Encarnação R, Alves P (2025). Pressure injury prediction in intensive care units using artificial intelligence: a scoping review. Nurs Rep.

[10] Gallo RJ, Shieh L, Smith M (2024). Effectiveness of an artificial intelligence-enabled intervention for detecting clinical deterioration. JAMA Intern Med.

[11] Schwabe K, Ferizaj D, Neumann S, Yurish SY (2025). Reducing nurses’ workload with an AI speech assistant for documentation.

[12] Ferizaj D, Neumann S (2024). Assessing perceptions and experiences of an AI-driven speech assistant for nursing documentation: a qualitative study in german nursing homes.

[13] Seibert K, Domhoff D, Fürstenau D, Biessmann F, Schulte-Althoff M, Wolf-Ostermann K (2023). Exploring needs and challenges for AI in nursing care – results of an explorative sequential mixed methods study. BMC Digit Health.

[14] von Gerich H, Moen H, Block LJ (2022). Artificial Intelligence -based technologies in nursing: a scoping literature review of the evidence. Int J Nurs Stud.

[15] Feuerriegel S, Frauen D, Melnychuk V (2024). Causal machine learning for predicting treatment outcomes. Nat Med.

[16] (2025). State of the world’s nursing 2025. https://www.who.int/publications/i/item/9789240110236.

[17] (2024). EFN policy statement on improving frontline nurses’ time for direct patient care with digitalisation and responsible AI. https://efn.eu/wp-content/uploads/2024/10/EFN-PS-improving-frontline-nurses-time-for-direct-patient-care-with-digitalisation-responsible-AI-Oct.-2024.pdf.

[18] Ball Dunlap PA, Michalowski M (2024). Advancing AI data ethics in nursing: future directions for nursing practice, research, and education. JMIR Nurs.

[19] Buchanan C, Howitt ML, Wilson R, Booth RG, Risling T, Bamford M (2020). Predicted influences of artificial intelligence on the domains of nursing: scoping review. JMIR Nurs.

[20] Akbarighatar P, Pappas I, Vassilakopoulou P (2023). A sociotechnical perspective for responsible AI maturity models: findings from a mixed-method literature review. Int J Inf Managment Data Insights.

[21] Jöhnk J, Weißert M, Wyrtki K, Not AC (2021). Ready or not, AI comes— an interview study of organizational AI readiness factors. Bus Inf Syst Eng.

[22] Fountaine T, McCarthy B, Saleh T (2019). Building the AI-Powered Organization. Technology isn’t the biggest challenge. Culture is. Harv Bus Rev.

[23] Becker J, Knackstedt R, Pöppelbuß J (2009). Developing maturity models for IT management. Bus Inf Syst Eng.

[24] Lasrado LA, Vatrapu R, Andersen KN (2015). Maturity models development in IS research: a literature review. https://aisel.aisnet.org/iris2015/6/.

[25] Pumplun L, Fecho M, Wahl N, Peters F, Buxmann P (2021). Adoption of machine learning systems for medical diagnostics in clinics: qualitative interview study. J Med Internet Res.

[26] Page MJ, McKenzie JE, Bossuyt PM (2021). The PRISMA 2020 statement: an updated guideline for reporting systematic reviews. BMJ.

[27] Tong A, Sainsbury P, Craig J (2007). Consolidated Criteria for Reporting Qualitative Research (COREQ): a 32-item checklist for interviews and focus groups. Int J Qual Health Care.

[28] O’Cathain A, Murphy E, Nicholl J (2008). The quality of mixed methods studies in health services research. J Health Serv Res Policy.

[29] Seibert K, Domhoff D, Altona J, Yurish SY (2025). ProKIP: rationale and development of the AI-nursing-care-readiness-assessment (AINCRA). https://sensorsportal.com/AIMH_2025/AIMH_2025_Proceedings.pdf.

[30] Chang A (2020). Artificial Intelligence and Human Cognition in Clinical Medicine and Healthcare.

[31] Boren SA, Moxley D (2015). Systematically reviewing the literature: building the evidence for health care quality. Mo Med.

[32] Ouzzani M, Hammady H, Fedorowicz Z, Elmagarmid A (2016). Rayyan-a web and mobile app for systematic reviews. Syst Rev.

[33] Palinkas LA, Horwitz SM, Green CA, Wisdom JP, Duan N, Hoagwood K (2015). Purposeful sampling for qualitative data collection and analysis in mixed method implementation research. Adm Policy Ment Health.

[34] Willis GB (2015). Analysis of the Cognitive Interview in Questionnaire Design.

[35] Doctor E, Eymann T, Fürstenau D (2023). A maturity model for assessing the digitalization of public health agencies. Bus Inf Syst Eng.

[36] Elo S, Kyngäs H (2008). The qualitative content analysis process. J Adv Nurs.

[37] (2026). AI nursing care readiness assessment (AINCRA): a tool for structured reflection and preparation of AI projects in nursing care. ProKIP.

[38] Abuzaid MM, Elshami W, Tekin H, Issa B (2022). Assessment of the willingness of radiologists and radiographers to accept the integration of artificial intelligence into radiology practice. Acad Radiol.

[39] Andersson J, Nyholm T, Ceberg C (2021). Artificial intelligence and the medical physics profession - a Swedish perspective. Phys Med.

[40] Weinert L, Müller J, Svensson L, Heinze O (2022). Perspective of information technology decision makers on factors influencing adoption and implementation of artificial intelligence technologies in 40 German hospitals: descriptive analysis. JMIR Med Inform.

[41] Alami H, Lehoux P, Denis JL (2020). Organizational readiness for artificial intelligence in health care: insights for decision-making and practice. J Health Organ Manag.

[42] Wiljer D, Hakim Z (2019). Developing an artificial intelligence-enabled health care practice: rewiring health care professions for better care. J Med Imaging Radiat Sci.

[43] Jennett P, Yeo M, Pauls M, Graham J (2003). Organizational readiness for telemedicine: implications for success and failure. J Telemed Telecare.

[44] Pumplun L, Tauchert C, Heidt M (2019). A new organizational chassis for artificial intelligence - exploring organizational readiness factors. https://aisel.aisnet.org/ecis2019_rp/106/.

[45] Someren MW, Barnard YF, Sandberg JAC (1994). The Think Aloud Method A Practical Guide to Modelling Cognitive Processes.

[46] Wolcott MD, Lobczowski NG (2021). Using cognitive interviews and think-aloud protocols to understand thought processes. Curr Pharm Teach Learn.

[47] Peute LWP, de Keizer NF, Jaspers MWM (2015). The value of retrospective and concurrent think aloud in formative usability testing of a physician data query tool. J Biomed Inform.

[48] Helgesson YYL, Höst M, Weyns K (2012). A review of methods for evaluation of maturity models for process improvement. J Software Evolu Process.

[49] Wolf-Ostermann K, Fürstenau D, Theune S Concept for embedding AI systems in nursing care: exploratory project on AI in nursing care (sokip). https://media.suub.uni-bremen.de/entities/publication/7f4e91a6-6598-4b1c-bec7-fcbf513dd8eb.

[50] Nyström ME, Karltun J, Keller C, Andersson Gäre B (2018). Collaborative and partnership research for improvement of health and social services: researcher’s experiences from 20 projects. Health Res Policy Syst.

[51] Greenhalgh T, Abimbola S (2019). The NASSS framework - a synthesis of multiple theories of technology implementation. Stud Health Technol Inform.

