# Supplementary material for: Readiness Assessment for AI in Nursing Care Projects: Multimethods Study
Source: JMIR Nurs. 2026 Jun 2;9:e84148. doi: 10.2196/84148 (PMC13229396; doi:10.2196/84148)
Supplement: Multimedia Appendix 1 [file nursing-v9-e84148-s001.pdf]

Table 1: English language search terms.

Block1: Artificial Intelligence	Block 2: Readiness	Block 3: Assessment
artificial intelligence<br>machine learning	readiness<br>preparedness<br>fitness<br>maturity	assessment<br>evaluation<br>tool<br>checklist<br>instrument<br>framework<br>model

Table 2: Guiding questions for group discussions.

Evaluation criteria	Guiding question: "With regard to the AINCRA dimension presented to you …"
Benefits and consequences	What benefits do you see, and when, from applying the AINCRA?<br>What benefits or consequences do you see resulting from the AINCRA results?
Rater-entity	Who should carry out the AINCRA rating for the attributes?
Attainability of AINCRA Levels	How do you assess the attainability of the AINCRA levels?
Achievement of AINCRA levels	When you think of AINC projects that were, in your view, successful:<br>What levels did these projects have at the start?<br>What levels were reached by the end of the project, or up to which levels did development occur?
Step size of AINCRA levels	How do you assess the step size between the AINCRA levels?
Influenceability	How do you assess the influenceability of the attributes by the actors in AINC projects?<br>Which attributes are particularly influenced by external actors or contextual factors?
Availability	How do you assess the availability of the information or the data needed to evaluate the attributes for actors in AINC projects?
Clarity and Comprehensibility	How do you assess the clarity and comprehensibility of the AINCRA?
Applicability	How do you assess the applicability of the AINCRA?
Comparability	How do you assess the comparability of evaluations obtained with the AINCRA across different AINC projects?
Completeness	Finally, how do you assess the completeness of the AINCRA?<br>Which attributes are missing and should be added?

AINC=AI in nursing care; AINCRA= AI in nursing care readiness assessment.

Table: 3 Number of comments obtained from think aloud interviews and changes made to pilot AINCRA version.

AINCRA dimension	Attributes in dimension	Attributes with expert comments	N expert comments			Changes to AINCRA pilot version: Suggested by experts	Realized	Realized
	N		Min	Max	Mean	N		%
Regulatory requirements and aspects	9	9	2	3	2.7	6	3	50.00%
Processual and translational requirements and aspects	41	38	0	9	4.9	64	27	42.19%
Technical Requirements and Aspects	4	4	3	5	4.0	2	2	100.00%
Social and ethical requirements and aspects	10	10	4	5	4.0	6	4	66.67%
Community Building requirements and aspects	3	3	3	4	3.7	1	1	100.00%
Total	67	64				70	37	52.86%

Table 4: Characteristics of think aloud interview and group discussion participants.

Interview number	AINC project stakeholder group, setting, position/ role	Experience with AINC projects	AINC project reflected on in think aloud interview is	AINCRA dimension assessed in think aloud interview	Involved in the German funding programme "Making Repositories and AI Systems Usable in Everyday Care"
1	Clinical partner, long-term care, project coordination	6-10 years	completed	PROC	N
2	Nursing/health science, university, project coordination	< 5 years	ongoing	REG<br>TECH<br>COM	Y
3	AI R&D, tech company, project coordination	> 10 years	completed	REG<br>TECH<br>SOC	N
4	Nursing/health science, clinical partner, hospital, project coordination	< 5 years	ongoing	SOC<br>COM	Y
5	Clinical partner, long-term care, project coordination	< 5 years	ongoing	PROC	N
6	Nursing/health science, university, project coordination	< 5 years	ongoing	PROC	N
7	Nursing/health science, research institute, project coordination	< 5 years	ongoing	PROC	Y
8	Nursing/health science, university, project coordination	< 5 years	ongoing	SOC<br>COM	N
9	Nursing/health science, university, project coordination	< 5 years	in planning	SOC	N
10	Nursing/health science, university, project coordination, nursing informatics	> 10 years	ongoing	PROC	N
11	Clinical partner, hospital, data science, project coordination, co-creation facilitator	6-10 years	completed	SOC<br>COM	N
12	Nursing/health science, university, hospital, project coordination	< 5 years	ongoing	PROC<br>COM	Y
13	Nursing/health science, clinical partner, university, hospital, project coordination, digitization	unclear	completed	PROC	N
14	AI R&D, research institute, model development	unclear	ongoing	REG<br>TECH<br>SOC	Y
15	AI R&D, research institute, model development	unclear	ongoing	PROC	Y
16	AI R&D, tech company, project coordination	unclear	ongoing	PROC	Y
17	AI R&D, research institute, model development	unclear	ongoing	REG<br>TECH<br>COM	Y
18	Clinical partner, hospital, IT infrastructure, AI model development	> 10 years	ongoing	REG<br>TECH	N

Y=Yes; N=No; AI R&D=Artificial Intelligence Research and Development; REG=Regulatory Requirements and Aspects; TECH=Technical Requirements and Aspects; PROC=Processual and Translational Requirements and Aspects; SOC= Social and Ethical Requirements and Aspects; COM=Community Building Requirements and Aspects. IT=Information Technology.

Table 5: Summary of expert ratings from the think aloud interviews.

Evaluation criteria	Summary of expert ratings and feedback (N=18)
**Benefits of AINCRA application**	**Overall beneficial**<br><br>**Clinical partners, AI R&D, and research partners in AINC projects**<br>• Deciding whether to participate<br>• Project planning<br>• Grant application<br>• Agreements between individual partners<br>• Agreements among multiple partners<br>• Identifying and arguing for:<br>o Human resources<br>o Internal expertise<br>o External expertise<br>o Training needs (for project leads, nursing professionals, and others)<br>• Aligning perspectives of project partners<br>• Reaching a common understanding of goals and perspectives<br>• Early identification of obstacles during the project<br>• As a project management tool<br>• As-is analysis<br>• Formative evaluation<br>• Summative evaluation<br><br>**Funding bodies**<br>Documentation of AINC projects<br>• Monitoring of AINC projects<br>• Interim evaluations of AINC projects<br>• Assessment of AINC projects<br>• Comparison of AINC projects<br><br>**Tech companies selling and implementing AI systems in care facilities and hospitals**<br>• Project management<br>• Consulting for companies<br>• Supporting the long-term use of AI systems in organizations<br><br>**Scientific (accompanying) research on AI care projects**<br>• Standardized documentation of AINC projects<br>• Cross-sectional or longitudinal analysis of AINC projects
**Consequences of AINCRA results**	**Identifying areas of improvement in AINC projects, including:**<br>• Deriving necessary and possible changes<br>• Basis for decisions on whether to continue or terminate a project<br>• Enables early evaluation of the practical utility and added value of the AI system<br>• Competitive advantage in grant selection for applicants with high AINCRA levels<br><br>**Positive or negative emotional responses:**<br>• Low AINCRA level placement may lead to<br>o Feelings of irritation, frustration, or disappointment<br>o Sparking ambition and interest in improvement<br>o Demotivation of clinical partners<br>o Opting out of clinical partners
**Rater-entity**	**Depending on dimension and attribute**<br>• Different individuals<br>• Collaborative or group assessment<br>o Tandem

	○ Inter-professional team<br>**Assessment of processual and translational requirements and aspects**<br>• Perspective of clinical partner is key<br>• Individuals with long-standing experience at the clinical partner organization, with backgrounds in nursing and digitalization<br>• IT specialists from the clinical partner working together with nurse managers and leaders<br>• Collaboration between AI R&D and clinical partner<br>• Participatory, guided completion of the AINCRA with nurses directly involved in the AI system's field of application
**Achievability of AINCRA levels**	**Overall achievable**<br>**Level 5**<br>• Currently not or hardly achievable for clinical partners<br>• Ambitious and difficult to reach<br>• Future goal<br>• Achievable for clinical partners implementing many AINC projects<br>• Not realistic for classic R&D projects<br>**Level 4**<br>• Maximum realistically achievable level for R&D projects<br>• Desirable and satisfactory for clinical partners<br>**Level 3**<br>• Desirable and satisfactory for clinical partners<br>**Level 2**<br>• Not mentioned<br>**Level 1**<br>• Some clinical partners start AINC projects above this level
**Achievement of AINCRA levels**	**Depending on goals and structures of an AINC project**<br>**Progressive nature of AINC projects**<br>**Development of trajectories**<br>• Advancement by one or more levels<br>• Remaining at the same level<br>• Regression to a lower level<br>**Entry levels**<br>• Facilitate selection of suitable clinical partners<br>• Data quality: Level 1 is a knockout criterion<br>• Data availability: Level 1 is a knockout criterion<br>• Not needed for each attribute
**Step size of AINCRA levels**	**Sufficient**<br>**Appropriate**<br>**Distinct (clearly differentiated)**
**Influenceability**	**Overall influenceable**<br>**Strong influenceability of**<br>• Attitudes<br>• Acceptance<br>• Knowledge<br>• Willingness to change<br>**Limited influenceability of**<br>• Fundamental corporate decisions<br>• External conditions of clinical partners

**Availability**	**Overall available or attainable with reasonable effort**<br>**Facilitated by AINC project stakeholder involvement**
**Clarity and Comprehensibility**	**Understandable for AINC project leaders**<br>**Low-threshold**<br>**Details are not quickly grasped**<br>**Request for changes or explanation of**<br>• Order of attributes<br>• Abbreviations<br>• Selected terms<br>• Descriptions of levels
**Applicability**	**Overall applicable**<br>**Inter-professional assessment improves applicability**<br>**AINCRA is complex and text-heavy**<br>• Difficult to apply without prior AINC experience<br>**Time Effort**<br>• Time-consuming initial use<br>• Understanding takes time<br>• Long duration may lower acceptance to carry out the AINCRA<br>• Application becomes easier over time, familiarity reduces effort
**Comparability**	**May be difficult**<br>**Can be ensured through external evaluation**<br>**Comparability possible of**<br>• Individual attributes or dimensions<br>• Multiple clinical partners within one AINC project<br>• Multiple AINC projects within the same funding programme
**Completeness**	**Overall complete**<br>**Suggestions for additions or change**<br>• Include staff representatives as important stakeholders<br>• Allow custom attributes to be added by users<br>• Enable mapping of multiple clinical partners within a single AINC project

AINC=AI in nursing care; AINCRA= AI in nursing care readiness assessment.

Researcher's background and relationship with participants

Researchers involved in data collection (KS, JA, LB, AN, RG, KB) are all members of the accompanying scientific research for the German funding programme "Making Repositories and AI Systems Usable in Everyday Care", with the principal data collectors (KS, DD, LB) being significantly involved in a preliminary study on AINC needs and challenges [13] and familiar in person with 13 experts in total while having extensive experience in conducting online interviews and focus group formats. Researchers involved in qualitative content analysis (KS, LB, AN, JA, JP) are all members of the accompanying scientific research project and include perspectives from nursing or health science, information and data science, and clinical partners. KS, DD, and JA hold a doctorate or M.Sc./M.A. degree in nursing or health science, LB holds a M.A. degree in communication science with a focus on psychology, AN has a M.Sc. in business informatics and solid experience in conducting think aloud interviews, RG has a M.Sc. in corporate management, KB has a doctorate in philosophy, and JP holds a doctorate in computer science. Further, DD, SJ, DF, FB, and KWO were involved in consensus building and commenting on the final AINCRA terminology. SJ has a M.Sc. in data science. DF, FB, and KWO were project leads for the preliminary study cited above and hold professorships for business administration with a focus on IT management and digital transformation, machine learning, and healthcare research, respectively. DF has conducted prior research on maturity model building for the digitalization of public health agencies.
