# Supplementary material for: Readiness Assessment for AI in Nursing Care Projects: Multimethods Study
Source: JMIR Nurs. 2026 Jun 2;9:e84148. doi: 10.2196/84148 (PMC13229396; doi:10.2196/84148)
Supplement: Multimedia Appendix 2 [file nursing-v9-e84148-s002.pdf]

Gefördert durch:
Bundesministerium
für Forschung, Technologie
und Raumfahrt

# AI Nursing Care Readiness Assessment (AINCRA) Instrument

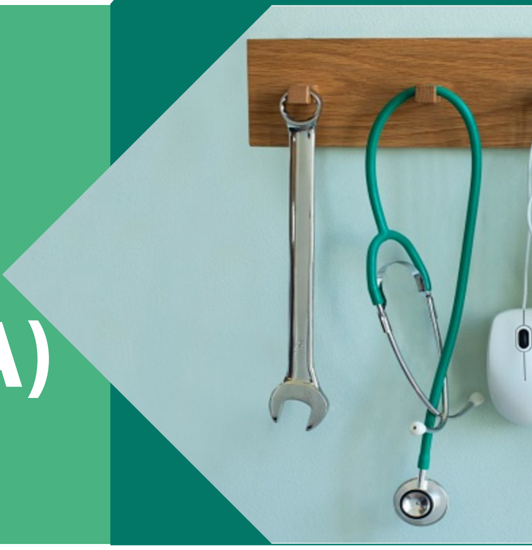

A result of the accompanying scientific research in the BMFTR funding program “Making Repositories and AI Systems Usable in Everyday Care.”

July 2025

ProKIP

Gefördert durch:
Bundesministerium für Forschung, Technologie und Raumfahrt

| Dimension 1<br>Regulatory Requirements and Aspects | | | | | | | | | |
|---|---|---|---|---|---|---|---|---|---|
| **Attribute Number** | **Subdimension/ Attribute** | **Level 1 (initial)** | **Level 2 (assessing)** | **Level 3 (determined)** | **Level 4 (managed)** | **Level 5 (optimised)** | **Not applicable** | **(Joint) Assessment by (Rater Entity)** | **Source** |
| **1.1** | **Analysis of the data set: Information content (despite anonymisation)** | The information content of the data required for the AINC project is unknown or the data exist as non-anonymised personal data. | The data required for the AINC project are available as anonymised data, with an almost complete loss of diagnostically relevant informational value. | The data required for the AINC project are available as anonymised data, with a low level of diagnostically relevant informational value. | The data required for the AINC project are available as anonymised data, with a medium level of diagnostically relevant informational value. | The data required for the AINC project are available as anonymised data, with preserved diagnostically relevant informational value. | Not applicable | AI R&D<br>Nursing Science<br>Clinical Partner | 1 |
| **1.2** | **Analysis of the data set: Representativeness of training data** | Unknown or almost no alignment between the data and the intended application context of the AI system (e.g., demographics, region, or work culture) in the training data of the AINC project. | Low alignment between the data and the intended application context of the AI system (e.g., demographics, region, or work culture) in the training data of the AINC project. | Moderate alignment between the data and the intended application context of the AI system (e.g., demographics, region, or work culture) in the training data of the AINC project. | High alignment between the data and the intended application context of the AI system (e.g., demographics, region, or work culture) in the training data of the AINC project. | Complete alignment between the data and the intended application context of the AI system (e.g., demographics, region, or work culture) in the training data of the AINC project. | Not applicable | AI R&D<br>Nursing Science | 1,2 |
| **1.3** | **Analysis of the data set: Quality** | Data quality is unknown or there is no consistent data quality (e.g., handwritten or synonym-based diagnoses) in the data required for the AINC project [from the clinical partner]. | Initial demands and considerations regarding the standardisation of data quality [of the clinical partner] in the AINC project, or execution of a data quality assessment (e.g., Pandas Profile). | Internal operational quality standards (e.g., for key terminologies) [at the clinical partner] in the AINC project. | Cross-organisational quality standards (e.g., for key terminologies) [at the clinical partner] in the AINC project. | International quality standards (e.g., Unified Medical Language System (UMLS)) implemented [at the clinical partner] in the AINC project. | Not applicable | AI R&D<br>Clinical Partner | 1,2 |
| **1.4** | **Analysis of the data set: Availability** | The data required for the AINC project are barely or not at all digitally available [at the clinical partner]. | Initial efforts to centrally collect and provide the data required for the AINC project [at the clinical partner].<br><br>However, data availability is sporadic and often incomplete. Basic storage systems exist, but the data are frequently outdated or only available for specific areas. | The data required for the AINC project are stored in central systems [at the clinical partner] and are digitally available to multiple departments.<br><br>Data availability is more consistent, but occasional bottlenecks still occur.<br><br>Data topicality is regularly checked, but not always ensured in real time. | The digital data required for the AINC project are consistently available throughout operations [at the clinical partner] and is centrally and automatically managed.<br><br>Data availability is high, with clear processes for data management.<br><br>Most data are accessible in real time and regularly updated to meet operational requirements. | The digital data required for the AINC project are available at all times and in real time throughout the entire organisation [of the clinical partner].<br><br>Data availability is ensured through redundancy and advanced technologies.<br><br>Data management processes are fully optimised, and data availability proactively supports operational decisions and strategic goals. | Not applicable | AI R&D<br>Clinical Partner | 1-4 |

Gefördert durch:
Bundesministerium für Forschung, Technologie und Raumfahrt

| Dimension 1<br>Regulatory Requirements and Aspects | | | | | | | | | |
|---|---|---|---|---|---|---|---|---|---|
| **Attribute Number** | **Subdimension/ Attribute** | **Level 1 (initial)** | **Level 2 (assessing)** | **Level 3 (determined)** | **Level 4 (managed)** | **Level 5 (optimised)** | **Not applicable** | **(Joint) Assessment by (Rater Entity)** | **Source** |
| 1.5 | **Analysis of the data set: Access** | The access path to the data required for the AINC project is unknown or there is no systematic access to the data [at the practice partner].<br><br>Data are either not collected or only available locally and in unstructured form.<br>Access to data is sporadic and manual, often only on request. | Initial steps are being taken to collect and gather data needed for the AINC project [at the practice partner].<br><br>However, access is limited and often restricted to specific departments or systems.<br><br>Data are partially outdated and access is unreliable and inefficient. | A systematic approach to collecting and storing the data required for the AINC project [at the practice partner] is being pursued.<br><br>Operational data is available to relevant user groups, though not yet consistently in real time.<br><br>Data are partially integrated and there are defined processes for access. | Access to the data required for the AINC project [at the practice partner] are consistently available and largely automated.<br><br>Data are current, consistent, and accessible in real time.<br><br>A centralised system enables controlled access to data across departments. | Access to the data required for the AINC project [at the practice partner] is fully integrated and optimised.<br><br>Data are available in real time at all times to all authorised users.<br><br>Advanced analytics tools and automated processes ensure efficient and secure access, which is continuously optimised. | Not applicable | AI R&D<br>Clinical Partner | 2,3,5 |
| 1.6 | **Data sharing models** | Data sharing models relevant to the AINC project are unknown or unclear.<br><br>No considerations regarding appropriate models and approaches exist. | Initial considerations and steps toward defining and implementing a data sharing model are being taken.<br><br>However, the model is not finalised, and necessary people and procedures are not yet fully clarified. | The data sharing model and approach are defined, and most legal, organisational, and technical requirements are identified.<br><br>Contact persons and responsibilities are known, but detailed negotiation and implementation have not started. | The data sharing model and approach are defined and known to all project participants.<br><br>All legal, organisational, and technical requirements are fully identified and documented in a structured manner.<br><br>Responsible contacts are involved and took part in detail negotiations.<br><br>Implementation includes appropriate regulatory, organisational, and technical safeguards | The model and processes of data sharing have been implemented with all necessary regulatory, organisational, and technical safeguards.<br><br>Fulfilment of requirements is proven (e.g., through internal/external certification).<br><br>Data sharing and usage are fully documented, and documentation is regularly reviewed.<br><br>Processes and structures evolve based on audit results. | Not applicable | AI R&D | 2,3,5 |

| Dimension 1<br>Regulatory Requirements and Aspects | | | | | | | | | |
|---|---|---|---|---|---|---|---|---|---|
| **Attribute Number** | **Subdimension/ Attribute** | **Level 1 (initial)** | **Level 2 (assessing)** | **Level 3 (determined)** | **Level 4 (managed)** | **Level 5 (optimised)** | **Not applicable** | **(Joint) Assessment by (Rater Entity)** | **Source** |
| **1.7** | **EU Medical Device Regulation (EU MDR) (and/or, although phasing out, MPG)**<br><br>(Note: MPG = German Medical Devices Act) | No consideration of EU MDR or MPG in the AINC project, or relevant content is unknown. | Initial steps to comply with EU MDR or other relevant medical device regulations in the AINC project.<br><br>A basic overview of legal requirements exists, but they are only partially, unsystematically, or inconsistently implemented.<br><br>Responsibilities (e.g., sponsor role) and required persons or institutions are not fully clarified. | The AINC project is committed to comprehensive compliance with EU MDR or other relevant medical device regulations.<br><br>A broad overview of legal requirements has been obtained, and project responsibilities have been defined.<br><br>Many requirements have been or are planned to be implemented.<br><br>However, full coverage is not ensured, and implementation is only partly documented, tested, or evaluated. | The AINC project is committed to full compliance with EU MDR or other medical device regulations.<br><br>A structured overview of all relevant legal requirements has been created and shared with participants.<br><br>Responsibilities are defined, involved parties are integrated, and implementation follows a defined standard.<br><br>Implementation is planned or completed with adequate resources.<br><br>Documentation and evaluation are regular and comprehensive. | The AINC project ensures full compliance with EU MDR or other medical device regulations.<br><br>A complete and structured legal requirement overview exists and is shared with all participants.<br><br>Responsibilities are clear.<br><br>Implementation follows national or international standards (e.g., ISO 13485).<br><br>All requirements are fulfilled, documented, tested, and externally certified.<br><br>Regular monitoring and updates ensure lasting compliance. | Not applicable | AI R&D<br>Nursing Science<br>Clinical Partner | 2,5,6 |
| **1.8** | **EU GDPR and sector-specific laws** | No consideration of GDPR or other relevant data protection regulations in the AINC project.<br><br>No awareness of which parts of the project are affected. | Initial steps to comply with GDPR or similar regulations are being taken.<br><br>A basic overview exists, but requirements are only partly, inconsistently, or unsystematically implemented.<br><br>Responsibilities and external involvement are not fully clarified. | The AINC project is committed to GDPR compliance and has obtained a broad overview of legal requirements.<br><br>Responsibilities are defined, many requirements are planned or implemented, but completeness is not ensured, and documentation/testing is partial. | The AINC project is committed to GDPR compliance.<br><br>A structured overview of all relevant requirements exists and has been communicated.<br><br>Responsibilities are defined and implementation follows a standard process.<br>All requirements are planned or implemented with sufficient resources.<br><br>Implementation is well-documented, tested, and regularly reviewed. | Full and demonstrable GDPR compliance is ensured.<br><br>A structured legal framework is known to all participants.<br><br>Implementation follows national/international standards (e.g., Standard Data Protection Model).<br><br>All requirements are implemented, tested, documented, and externally certified.<br><br>Regular review and improvement cycles are in place. | Not applicable | AI R&D<br>Nursing Science<br>Clinical Partner | 2,5,6 |

| Dimension 1<br>Regulatory Requirements and Aspects | | | | | | | | | |
|---|---|---|---|---|---|---|---|---|---|
| **Attribute Number** | **Subdimension/ Attribute** | **Level 1 (initial)** | **Level 2 (assessing)** | **Level 3 (determined)** | **Level 4 (managed)** | **Level 5 (optimised)** | **Not applicable** | **(Joint) Assessment by (Rater Entity)** | **Source** |
| **1.9** | **EU AI Act** | No consideration of the EU AI Act in the AINC project.<br><br>No awareness of which parts of the project are affected. | Initial steps to comply with the EU AI Act are underway.<br><br>A basic overview exists, but the requirements are inconsistently implemented.<br><br>Responsibilities and external actors are not fully clarified. | The AINC project is committed to compliance with the EU AI Act.<br><br>A broad overview exists, responsibilities are defined.<br><br>Many requirements are implemented or planned, but coverage and documentation are not complete. | The AINC project is committed to AI Act compliance and has a structured overview of legal requirements.<br><br>Responsibilities are clear and implementation follows a defined process standard.<br><br>All requirements are planned or fulfilled with explicit deadlines and sufficient resources (time, personnel, other).<br><br>Implementation is well-documented, tested, and regularly monitored. | Full compliance with the EU AI Act is ensured.<br>A complete and structured legal framework exists and is shared with all participants.<br><br>Responsibilities are clear, and implementation follows international standards (e.g., ISO/IEC 42001).<br><br>All requirements are planned or fulfilled with explicit deadlines and sufficient resources (time, personnel, other), documented, tested, externally certified, and continuously improved. | Not applicable | AI R&D<br>Nursing Science<br>Clinical Partner | PROKIP |

| Dimension 2<br>Processual and Translational Requirements and Aspects | | | | | | | | | |
|---|---|---|---|---|---|---|---|---|---|
| **Attribute Number** | **Subdimension/ Attribute** | **Level 1 (initial)** | **Level 2 (assessing)** | **Level 3 (determined)** | **Level 4 (managed)** | **Level 5 (optimised)** | **Not applicable** | **(Joint) Assessment by (Rater Entity)** | **Source** |
| **2.1** | **Clinical Partner: Time Resources***<br>*Time resources may refer to leadership and nursing staff of the organisation, but also to other personnel (e.g., IT professionals or other occupational groups) who need to be planned accordingly. | Time resources for the planning, implementation, and evaluation of AINC projects [at the clinical partner] are unknown or not available or not intended.<br>Employees are fully occupied with routine tasks.<br>There are no clear time slots for planning, implementation, or evaluation of AINC projects. | Initial considerations for the allocation of time resources for AINC projects [at the clinical partner] exist.<br>However, there are no fixed schedules or clear assignments.<br>Employees only occasionally devote time to these projects, often outside their regular working hours, which leads to delays. | Specific time slots for the planning, implementation, and evaluation of AINC projects exist.<br>However, these resources are still limited and often have to compete with other tasks.<br>In some cases, employees are designated for AINC projects, but the time resources are not always sufficient. | Time resources for AINC projects are firmly scheduled [at the clinical partner] and are systematically managed.<br>There are clearly defined schedules that cover planning, implementation, and evaluation.<br>Employees regularly have dedicated time for AINC projects, and their progress is monitored and adjusted. | Time resources for AINC projects are fully integrated and optimised [at the clinical partner].<br>Employees have sufficient and flexible time slots to efficiently manage all phases of the KIP projects.<br>Time resources are continuously monitored, and there are mechanisms for rapid adjustment when needed to ensure project success. | Not applicable | Clinical Partner | 4 |
| **2.2** | **Clinical Partner: Personnel Resources*: Own staff position for implementing AI in the organisation**<br>*Personnel resources may refer to leadership and nursing staff of the organisation, but also to other personnel (e.g., IT professionals or other occupational groups) who need to be planned accordingly. | There is no dedicated position or role for the implementation of AI [at the clinical partner].<br>Responsibility for AI is not formally assigned, and AINC projects are either not undertaken at all or only sporadically and without clear leadership. | Initial considerations for creating a dedicated position for AI implementation [at the clinical partner] exist.<br>There may be staff members informally responsible or project-based teams, but there is no officially defined role or position.<br>AI-related tasks are carried out alongside other duties. | A dedicated position for the implementation of AI [at the clinical partner] is defined and established.<br>However, this role is still in development and may not be fully staffed.<br>The role has clear responsibilities, but resources and authority are still limited. | A dedicated position for the implementation of AI [at the clinical partner] is fully established, staffed, and known within the organisation.<br>This role is clearly integrated into the organisational structure and has the necessary resources and authority to effectively advance AI initiatives.<br>There are clear processes for collaboration with other departments. | A dedicated position for the implementation of AI [at the clinical partner] is fully optimised and strategically aligned.<br>This role is central to the organisation's innovation strategy and has comprehensive access to resources as well as influence on decision-making processes.<br>There is ongoing training and adaptation of the role to meet changing requirements. | Not applicable | Clinical Partner | 7 |

| Dimension 2<br>Processual and Translational Requirements and Aspects | | | | | | | | | |
|---|---|---|---|---|---|---|---|---|---|
| **Attribute Number** | **Subdimension/ Attribute** | **Level 1 (initial)** | **Level 2 (assessing)** | **Level 3 (determined)** | **Level 4 (managed)** | **Level 5 (optimised)** | **Not applicable** | **(Joint) Assessment by (Rater Entity)** | **Source** |
| **2.3** | | There is no dedicated position or role for participation in AI research and development (R&D) [at the clinical partner].<br>Participation in AI R&D is not formally assigned, and AINC projects are either not undertaken at all or only sporadically and without clear involvement. | Initial considerations for creating a dedicated position for participation in AI R&D [at the clinical partner] exist.<br>There may be staff members informally responsible or project-based teams, but no officially defined role or position.<br>Tasks related to participation in AI R&D are carried out alongside other duties. | A dedicated position for participation in AI R&D [at the clinical partner] is defined and established.<br>However, this role is still in development and may not be fully staffed.<br>The role has clear responsibilities, but resources and authority are still limited. | A dedicated position for participation in AI R&D [at the clinical partner] is fully established and staffed.<br>This role is clearly embedded in the organisational structure and has the necessary resources and authority to effectively drive R&D efforts.<br>There are clear processes for collaboration with other departments. | A dedicated position for participation in AI R&D [at the clinical partner] is fully optimised and strategically aligned.<br>This role is pivotal in shaping the organisation's AI R&D strategy and has comprehensive access to resources as well as influence on decision-making processes.<br>There is ongoing professional development and adaptation of the role to meet changing requirements. | Not applicable | Clinical Partner | 7 |
| **2.4** | **Clinical Partner: Personnel Resources: Available personnel resources for AINC projects and AI integration in the organisation** | There are no dedicated positions or roles for AINC projects and AI integration, e.g., in the IT department or management [at the clinical partner].<br>Responsibility for AINC projects is not formally assigned, and such projects are approached sporadically and without clear leadership. | Initial considerations for creating dedicated positions for AINC projects and AI integration [at the clinical partner] exist.<br>There may be informal persons responsible or project-based teams, but no officially defined role or position.<br>Tasks in AINC projects are carried out alongside other duties. | Dedicated positions for AINC projects and AI integration are defined and established [at the clinical partner].<br>However, these positions are still in development and may not be fully staffed.<br>The roles have clear responsibilities, but resources and authority are still limited. | Dedicated positions for AINC projects and AI integration [at the clinical partner] are fully established and staffed.<br>These roles are clearly embedded in the organisational structure and have the necessary resources and authority to effectively advance AI initiatives.<br>There are clear processes for collaboration with other departments. | Dedicated positions for AINC projects and AI integration [at the clinical partner] are fully optimised and strategically aligned.<br>These roles have comprehensive access to resources.<br>There is ongoing professional development and adaptation of the roles to meet changing requirements. | Not applicable | Clinical Partner | 4 |

| Dimension 2<br>Processual and Translational Requirements and Aspects | | | | | | | | | |
|---|---|---|---|---|---|---|---|---|---|
| **Attribute Number** | **Subdimension/ Attribute** | **Level 1 (initial)** | **Level 2 (assessing)** | **Level 3 (determined)** | **Level 4 (managed)** | **Level 5 (optimised)** | **Not applicable** | **(Joint) Assessment by (Rater Entity)** | **Source** |
| **2.5** | **Clinical Partner: Personnel Resources: Available Data Scientists***<br>*Data Scientists use data analytics, machine learning, and statistics based on the clinical partner's data to improve nursing care, optimise operations, and support research. Clinical partners employing their own data scientists find it easier to utilise structured and unstructured data in the organisation for AINC projects. | No data scientists are involved [at the clinical partner] in the AINC project.<br>External consulting is either not used at all or only very sporadically and as needed. | Initial considerations for hiring or contracting data scientists [at the clinical partner] exist, but there is still no permanently hired or contracted expertise within the project consortium/team. | One or more data scientists have been hired or contracted [at the clinical partner], but their integration and influence are still limited. | A sufficient number of data scientists are permanently integrated into the team [at the clinical partner] and are actively involved in AINC projects. | Data scientists are fully integrated into the team [at the clinical partner], strategically involved, and play a central role in the AINC project. | Not applicable | Clinical Partner with IT staff<br>AI R&D | 5 |
| **2.6** | **Clinical Partner: Personnel Resources: Available Data Champions***<br>*Data Champions understand the types of data generated at the clinical partner, advocate for proper data handling, and mediate between nursing staff and IT. They ensure data is correct, complete, and up to date, identify and resolve data collection issues, enhance staff data literacy through training, and raise awareness about the importance of data in nursing care and research. | There are no Data Champions [at the clinical partner] in the AINC project, and the use of data is not actively promoted or suppoyrted. | First Data Champions [at the clinical partner] are being identified, but their role is not yet formalised or recognised. | Some Data Champions [at the clinical partner] are active, their role is defined, and they promote a data culture in selected areas. | Data Champions [at the clinical partner] are established and work across departments to promote and support data use. | Data Champions are fully integrated into the organisational structure [of the clinical partner] and actively drive a data-driven culture forward. | Not applicable | Clinical Partner with IT staff<br>AI R&D | 5 |
| **2.7** | **Clinical Partner: General Degree of Digitisation***<br>*Overall digitisation level of the clinical partner's organisation and its different departments and data types (e.g., HR data, patient/care data, administrative data, etc.) | The general level of digitalisation [at the clinical partner] is unknown, or only non-digital data (e.g., paper-based records) exist. | Low proportion of digitised data in care/patient and personnel data (e.g., only available for central organisational processes) [at the clinical partner]. | Moderate proportion of digitised data in care/patient and personnel data (e.g., only central quality indicators) [at the clinical partner]. | High proportion of digitised data in care/patient and personnel data (e.g., time series data on disease progression) [at the clinical partner]. | Fully digitised data in care/patient and personnel records [at the clinical partner]. | Not applicable | Clinical Partner with IT staff<br>AI R&D | 1,4,5 |
| **2.8** | **Clinical Partner: AI-specific Degree of Digitisation** | The AI-specific level of digitalisation [at the clinical partner] is unknown, very low, or non-existent. AI systems are not used. | Initial steps toward digitalisation with a focus on AI are being explored [at the clinical partner], but have not yet been comprehensively implemented. | The clinical partner has integrated initial AI systems, but the level of digitalisation with a focus on AI is still limited to individual use cases or areas. | The clinical partner is largely digitalised, with a strong focus on the implementation and use of AI systems. | The clinical partner is fully digitalised, and AI systems are deeply integrated into all processes, with continuous optimisation of the level of digitalisation. | Not applicable | Clinical Partner with IT staff<br>AI R&D | 4,5 |

| Dimension 2<br>Processual and Translational Requirements and Aspects | | | | | | | | | |
|---|---|---|---|---|---|---|---|---|---|
| **Attribute Number** | **Subdimension/ Attribute** | **Level 1 (initial)** | **Level 2 (assessing)** | **Level 3 (determined)** | **Level 4 (managed)** | **Level 5 (optimised)** | **Not applicable** | **(Joint) Assessment by (Rater Entity)** | **Source** |
| **2.9** | **Clinical Partner: Degree of Digitisation: Data Quality Standards** | The data quality standard [at the clinical partner] is unknown or no uniform data quality standard exists. | Initial calls for standardizing data quality [at the clinical partner]. | Internal quality standards (e.g., for key terminologies such as the International Classification of Diseases – ICD) [at the clinical partner]. | Care facility-/clinic-wide and overarching quality standards (e.g., for key terminologies) [at the clinical partner]. | International quality standards [at the clinical partner]. | Not applicable | Clinical Partner with IT staff AI R&D | 1 |
| **2.10** | **Clinical Partner: Degree of Digitisation: Standard for Data Collection***<br>*Refers to a standard for collecting routine data generated in everyday care and organisational processes. | Processes for data collection [at the clinical partner] are unknown, or there are no standardised data collection processes. Data are collected sporadically and unsystematically. | Initial considerations for standardizing data collection [at the clinical partner] are being made, but have not yet been implemented. | Partially standardised data collection processes exist [at the clinical partner], but they are still incomplete. | Data collection [at the clinical partner] is largely standardised and systematic, but not consistently optimised. | Data collection [at the clinical partner] is fully standardised, systematic, and optimised, with regular review and adjustment. | Not applicable | Clinical Partner with IT staff AI R&D | 3 |
| **2.11** | **Clinical Partner: Degree of Digitisation: Standard for Data Storage** | Procedures for data storage [at the clinical partner] are unknown or no standardised procedures exist.<br><br>Data are stored unsystematically and in a decentralised manner. | Initial steps toward standardizing data storage [at the clinical partner] are being taken but not consistently implemented. | Partially standardised procedures for data storage exist [at the clinical partner], but they are still incomplete or have room for improvement. | Data storage [at the clinical partner] is largely standardised and centralised, with clear processes. | Data storage [at the clinical partner] is fully standardised, centralised, and optimised, with continuous improvement. | Not applicable | Clinical Partner with IT staff AI R&D | 3 |
| **2.12** | **Clinical Partner: Degree of Digitisation: Standard for Data Exchange** | The standard for data exchange [at the clinical partner] is unknown, or data exchange occurs sporadically and unsystematically without established standards. | Initial approaches to standardizing data exchange [at the clinical partner] exist but are not yet fully implemented. | Partially standardised procedures for data exchange exist [at the clinical partner], but they are still incomplete or need optimisation. | Data exchange [at the clinical partner] is largely standardised and efficient, but not consistently optimised. | Data exchange [at the clinical partner] is fully standardised, efficient, and optimised, with clear, established processes. | Not applicable | Clinical Partner with IT staff AI R&D | 3 |

| Dimension 2<br>Processual and Translational Requirements and Aspects | | | | | | | | | |
|---|---|---|---|---|---|---|---|---|---|
| **Attribute Number** | **Subdimension/ Attribute** | **Level 1 (initial)** | **Level 2 (assessing)** | **Level 3 (determined)** | **Level 4 (managed)** | **Level 5 (optimised)** | **Not applicable** | **(Joint) Assessment by (Rater Entity)** | **Source** |
| **2.13** | **Clinical Partner: Degree of Digitisation – Standard for Ontological Representation***<br>*Ontological representation refers to the structured depiction of knowledge in a specific domain (e.g., medicine), to make data uniform, interpretable, and high-quality. A standard for ontological representation helps structure and standardise data semantically, making it understandable, comparable, and interoperable. A hospital, for example, may use different terms for the same illness across different documents: “heart attack,” “myocardial infarction,” “ICD-10: I21.” An ontological representation would unify these terms under one standardised definition, allowing IT systems, healthcare professionals, and AI developers to clearly recognise they refer to the same condition. | The ontological representation of data [at the clinical partner] in the AINC project is unknown or no standards exist. | Initial considerations for introducing ontological standards [at the clinical partner] in the AI care project exist, but no concrete implementation has taken place. | Partially implemented ontological standards [at the clinical partner] in the AI care project exist, but still show gaps and inconsistencies. | Ontological standards are largely implemented and consistent [at the clinical partner] in the AINC project, but not fully optimised. | The ontological representation of data [at the clinical partner] in the AINC project is fully standardised, consistent, and optimised. | Not applicable | Clinical Partner with IT staff<br>AI R&D | 3 |
| **2.14** | **Clinical Partner: Willingness for Digital Transformation: Attitude and handling of AI in the organisation** | Willingness for digital transformation at the clinical partner in the AINC project is unknown or little to no willingness is shown; AI systems are viewed critically or ignored. | Initial steps toward digital transformation [at the clinical partner] in the AINC project are being taken; AI systems are cautiously accepted, but many reservations remain. | Digital transformation is actively promoted [at the clinical partner] in the AINC project, and AI systems are increasingly accepted, though some resistance remains. | High willingness for digital transformation [at the clinical partner] in the AINC project; AI systems are largely accepted and integrated into processes. | Full willingness for digital transformation [at the clinical partner] in the AINC project; AI systems are understood as a key part of the corporate strategy and actively supported. | Not applicable | Clinical Partner with IT staff<br>AI R&D | 3,5 |

| Dimension 2<br>Processual and Translational Requirements and Aspects | | | | | | | | | |
|---|---|---|---|---|---|---|---|---|---|
| **Attribute Number** | **Subdimension/ Attribute** | **Level 1 (initial)** | **Level 2 (assessing)** | **Level 3 (determined)** | **Level 4 (managed)** | **Level 5 (optimised)** | **Not applicable** | **(Joint) Assessment by (Rater Entity)** | **Source** |
| **2.15** | **Clinical Partner: Willingness for Digital Transformation: Support for AI from leadership and stakeholders***<br>*Stakeholders are all individuals or groups who influence the work climate in an organisation – such as managers, team leaders, employees, HR personnel, and sometimes external consultants – whose actions, decisions, and interactions shape the organisational culture, communication, trust, motivation, and overall work environment. | Leaders and stakeholders [at the clinical partner] do not support AI systems or do so only very limitedly. | First signs of support from leaders and stakeholders [at the clinical partner] exist, but they are still hesitant. | Leaders and stakeholders [at the clinical partner] actively support AI systems, but not yet consistently. | Strong support from leaders and stakeholders [at the clinical partner], who are actively engaged in AINC projects. | Leaders and stakeholders [at the clinical partner] fully support AI systems and are central drivers of the AI strategy within the organisation. | Not applicable | Clinical Partner | 5 |
| **2.16** | **Clinical Partner: Willingness for Digital Transformation: Organisation's own AI strategy** | There is no AI strategy in the company of the clinical partner within the AINC project. | Initial considerations for developing an AI strategy [at the clinical partner] exist, but no concrete direction yet. | An AI strategy is defined [at the clinical partner], but still in the early implementation phase and not fully transparent, communicated, or integrated. | An AI strategy is implemented [at the clinical partner], transparent, communicated, and integrated into the business strategy, but still being optimised. | A fully integrated and optimised AI strategy [at the clinical partner], which is continuously reviewed and updated. | Not applicable | Clinical Partner | 1,3 |
| **2.17** | **Clinical Partner: Willingness for Digital Transformation: Organisation's own data governance strategy***<br>*Data governance formalises decision rights, procedures, and controls to resolve conflicts of interest in data processing and sharing among involved actors. This requires maximizing the value of data and minimizing the risks depending on each stakeholder's perspective. | No data governance strategy [at the clinical partner] exists. | Initial considerations for developing a data governance strategy [at the clinical partner] are underway, but no concrete implementation yet. | A data governance strategy is defined [at the clinical partner], but still in the early implementation phase and not fully transparent, communicated, or integrated. | A data governance strategy is implemented [at the clinical partner], transparent, communicated, and integrated into the business strategy, but still being optimised. | A fully integrated and optimised data governance strategy [at the clinical partner], which is continuously reviewed and updated. | Not applicable | Clinical Partner | 1,3-7 |

Gefördert durch:
Bundesministerium für Forschung, Technologie und Raumfahrt

| Dimension 2<br>Processual and Translational Requirements and Aspects | | | | | | | | | |
|---|---|---|---|---|---|---|---|---|---|
| **Attribute Number** | **Subdimension/ Attribute** | **Level 1 (initial)** | **Level 2 (assessing)** | **Level 3 (determined)** | **Level 4 (managed)** | **Level 5 (optimised)** | **Not applicable** | **(Joint) Assessment by (Rater Entity)** | **Source** |
| **2.18** | **Clinical Partner: Willingness for Digital Transformation: Organisation's own IT governance strategy***<br>*IT governance is the strategic framework ensuring that IT is used effectively, securely, and in compliance with regulations to support organisational goals. | No IT governance strategy [at the clinical partner] exists. | Initial considerations for developing an IT governance strategy [at the clinical partner] are underway, but no clear direction yet. | An IT governance strategy is defined [at the clinical partner], but still in the early implementation phase and not fully transparent, communicated, or integrated. | The IT governance strategy is implemented [at the clinical partner], transparent, communicated, and integrated into the business strategy, but still being optimised. | A fully integrated and optimised IT governance strategy [at the clinical partner], which is continuously reviewed and updated. | Not applicable | Clinical Partner | 3 |
| **2.19** | **Clinical Partner: Technology Acceptance: Acceptance of AI by leadership, nursing staff, and other stakeholders***<br>*Stakeholders are all relevant groups other than patients or care recipients - this may include legal guardians, general practitioners, or consultative services depending on the AINC project. A stakeholder analysis should be part of every AINC project plan. | The acceptance of AI systems by leaders, nursing professionals, and other stakeholders [at the clinical partner] is unknown or very low.<br><br>Nursing professionals largely reject the use of AI technologies in the workplace or have strong reservations. | First signs of acceptance by leaders, nursing professionals, or other stakeholders [at the clinical partner], but scepticism and reluctance remain widespread across all groups. | Acceptance of AI is growing; leaders, nursing professionals, and other stakeholders [at the clinical partner] show increasing interest in using AI systems, but uncertainties and resistance persist. | High acceptance of AI technologies; leaders, nursing professionals, and other stakeholders [at the clinical partner] actively support the implementation of AI systems and the execution of AINC projects. | Full acceptance of AI systems; leaders, nursing professionals, and other stakeholders [at the clinical partner] proactively drive AI integration.<br><br>Nursing professionals fully accept the use of AI systems at the workplace and see them as valuable tools supporting their work. | Not applicable | Clinical Partner | 3 |
| **2.20** | **Clinical Partner: Technology Acceptance: Acceptance of AI by care recipients, patients, and their relatives** | The acceptance of AI systems by care recipients, patients, and their relatives is unknown, or care recipients/patients largely reject AI technologies in care or have strong reservations. | First signs of acceptance of AI systems by care recipients, patients, and their relatives, but scepticism remains widespread. | Acceptance of AI is growing; care recipients, patients, and their relatives increasingly accept AI systems in their care, but uncertainties and resistance still exist. | High acceptance of AI systems by care recipients, patients, and their relatives, who increasingly recognise their benefits in care. | Care recipients, patients, and their relatives fully accept AI systems and see them as a valuable part of their care. | Not applicable | Clinical Partner<br>Nursing Science | 1,5,6 |

| Dimension 2<br>Processual and Translational Requirements and Aspects | | | | | | | | | |
|---|---|---|---|---|---|---|---|---|---|
| **Attribute Number** | **Subdimension/ Attribute** | **Level 1 (initial)** | **Level 2 (assessing)** | **Level 3 (determined)** | **Level 4 (managed)** | **Level 5 (optimised)** | **Not applicable** | **(Joint) Assessment by (Rater Entity)** | **Source** |
| 2.21 | **Clinical Partner: Technology Acceptance: Acceptance of AI by staff representatives and their inclusion** | The acceptance of AI systems by staff representatives (e.g., employee council, personnel council) [at the clinical partner] is unknown or very low.<br>Staff representatives are (still) not involved in the implementation of the AINC project or largely reject the use of AI technologies in the workplace or have strong reservations. | First signs of acceptance by staff representatives [at the clinical partner], but scepticism and reluctance remain widespread. Initial efforts to involve staff representatives in the implementation of the AINC project exist, but these are still unsystematic and undocumented. | Acceptance of AI is growing; staff representatives show increasing interest in using AI systems, but uncertainties and resistance persist.<br>The involvement of staff representatives in the implementation of the AINC project is systematically planned but still incomplete, or significant clarification needs or ongoing coordination processes with staff representatives currently hinder progress of the AINC project. | High acceptance of AI technologies; staff representatives [at the clinical partner] actively support the implementation of AI systems and the execution of AINC projects. | Full acceptance of AI systems; staff representatives [at the clinical partner] proactively drive AI integration and see it as a valuable tool to support employees [of the clinical partner]. | Not applicable | Clinical Partner<br>Nursing Science | 1,6 |
| 2.22 | **Clinical Partner: Expectations and concerns** | Expectations and reservations regarding AI systems [from the clinical partner] are unknown or are not systematically recorded or considered. | Initial approaches to capturing expectations and reservations regarding AI systems at the clinical partner exist, but they are hardly systematic or integrated into the project workflow. | Expectations and reservations regarding AI systems are systematically recorded, but there are still gaps in the systematic collection and consideration of these expectations and reservations. | Expectations and reservations regarding AI systems at the clinical partner are systematically and comprehensively recorded and actively incorporated into project planning and implementation. | Expectations and reservations regarding AI systems at the clinical partner are continuously recorded, proactively addressed, and integrated into the strategic planning of the AI nursing project. | Not applicable | Clinical Partner<br>Nursing Science | PROKIP |
| 2.23 | **Clinical Partner: Knowledge and Competencies: AI and digital skills education, training, and continuing education in the organisation** | No training, advanced training, or continuing education offerings on AI systems and digital competence exist [at the clinical partner]. | Initial training and continuing education on AI systems and digital competence are offered [at the clinical partner], but only sporadically and without a clear structure. | Regular training and continuing education on AI systems and digital competence are offered [at the clinical partner], but not yet comprehensively utilised by the appropriate staff. | Comprehensive training, advanced training, and continuing education offerings on AI systems and digital competence are established [at the clinical partner] and are regularly used. | Fully integrated training, advanced training, and continuing education strategies on AI systems and digital competence [at the clinical partner], which are continuously adapted and optimised. | Not applicable | Clinical Partner<br>Nursing Science<br>AI R&D | 1,5 |
| 2.24 | **Clinical Partner: Knowledge and Competencies: Available AI knowledge among staff** | The knowledge of staff in the AINC project [and at the clinical partner] is unknown, or staff has little to no knowledge about AI. | Initial steps to convey knowledge are being taken in the AINC project and at the clinical partner, but the available AI knowledge is still limited. | Staff has basic AI knowledge, but there are still significant knowledge gaps. | Staff has a comprehensive understanding of AI systems, which is applied in most areas. | Staff possesses deep and up-to-date AI knowledge, which is continuously expanded and kept current. | Not applicable | Clinical Partner | 3,8 |

| Dimension 2<br>Processual and Translational Requirements and Aspects | | | | | | | | | |
|---|---|---|---|---|---|---|---|---|---|
| **Attribute Number** | **Subdimension/ Attribute** | **Level 1 (initial)** | **Level 2 (assessing)** | **Level 3 (determined)** | **Level 4 (managed)** | **Level 5 (optimised)** | **Not applicable** | **(Joint) Assessment by (Rater Entity)** | **Source** |
| **2.25** | **Clinical Partner: Knowledge and Competencies: Real-time and/or predictive analytics** | Capabilities for performing real-time or predictive analyses at the clinical partner are unknown or do not exist. | Initial approaches to performing real-time or predictive analyses at the clinical partner are being developed but are not yet applied systematically. | Real-time and predictive analyses are partially performed at the clinical partner but are not yet optimised or fully utilised. | The clinical partner regularly conducts real-time and predictive analyses, which are well integrated into the processes. | Real-time and predictive analyses are fully integrated and optimised at the clinical partner and are a key component of decision-making. | Not applicable | Clinical Partner | 1,4,6,8 |
| **2.26** | **Clinical Partner: Knowledge and Competencies: Available methods for knowledge transfer within the organisation** | Available methods for knowledge transfer at the clinical partner are unknown or no established methods exist. | Initial approaches for knowledge transfer are present but not yet systematically implemented. | Knowledge transfer is partially organised, but there are still gaps in consistency and dissemination. | Methods for knowledge transfer are established and regularly applied, but there is still potential for optimisation. | Knowledge transfer is fully systematised, optimised, and an integral part of the organisational culture. | Not applicable | Clinical Partner | 5 |
| **2.27** | **Clinical Partner: Intangible Assets: Availability and scope of intangible assets in the context of AI within the organisation***<br>*E.g., books, scholarly articles, awards, strategic partnerships, consultations. Intangible assets related to digitisation and digital competencies can also be valuable to clinical partners in AINC projects. | Intangible assets related to AI at the clinical partner are unknown or there are none or very few intangible assets available. | Initial intangible assets are identified and occasionally used at the clinical partner, but not yet comprehensively. | A growing repertoire of intangible assets exists and is actively used at the clinical partner, but not yet fully integrated (e.g., not freely available to all relevant personnel). | Comprehensive collection and systematic use of intangible assets, which are regularly expanded and freely available to relevant personnel. | Comprehensive collection and systematic use of intangible assets, which are regularly expanded and freely available to relevant personnel.<br>Intangible assets are continuously reviewed and updated. | Not applicable | Clinical Partner | 6 |
| **2.28** | **Clinical Partner: Financial Resources and Investments: Available financial resources for AINC projects and AI integration***<br>*These may also be part of general digitisation budgets or other budgets (e.g., innovation or inclusion budgets). | No specific financial resources for AI nursing projects are available at the clinical partner. | Initial financial resources for AI nursing projects are provided at the clinical partner but in a limited scope. | Sufficient financial resources are allocated for most AI nursing projects at the clinical partner, but there is a lack of long-term planning. | Comprehensive and sustainable financial resources for AI nursing projects are available and regularly increased and adjusted. | Fully optimised and continuously adjusted financial resources for AI nursing projects at the clinical partner, strategically used for AINC projects and integration. | Not applicable | Clinical Partner | 5 |

ProKIP

Gefördert durch:
Bundesministerium für Forschung, Technologie und Raumfahrt

| Dimension 2<br>Processual and Translational Requirements and Aspects | | | | | | | | | |
|---|---|---|---|---|---|---|---|---|---|
| **Attribute Number** | **Subdimension/ Attribute** | **Level 1 (initial)** | **Level 2 (assessing)** | **Level 3 (determined)** | **Level 4 (managed)** | **Level 5 (optimised)** | **Not applicable** | **(Joint) Assessment by (Rater Entity)** | **Source** |
| **2.29** | **Financial Resources and Investments: Exploring alternative financing models for including clinical partners** | Alternative funding models for involving clinical partners are unknown or hardly considered at all. | Initial alternative financing models are being explored or developed, but not yet utilised. | Alternative financing models are being evaluated and partially used. | Established alternative financing models exist, which are regularly used and reviewed. | Alternative financing models are actively used, with regular reassessment and adaptation of the search for new alternative financing models to meet changing requirements. | Not applicable | Clinical Partner | 1,3-5 |
| **2.30** | **Financial Resources and Investments: Management: sustainable alignment of resources and investments** | No clear link between resources, investments, and project goals. | Initial steps are being taken to align resources and investments with project goals. | Resources and investments are largely aligned with project goals, but gaps remain. | Resources and investments are systematically aligned with project goals and sustainably secured. | Resources and investments are fully integrated and optimally aligned with the long-term goals and benefits of the project. | Not applicable | AI R&D<br>Nursing Science<br>Clinical Partner | 2 |
| **2.31** | **Research Objective: Needs or problems in nursing practice** | Research objectives of the AINC project are unknown or not aligned with the needs and problems of nursing practice. | Initial considerations are being made to derive research objective from needs and problems of nursing practice. | Research objectives are increasingly derived from specific practical needs, but not yet systematically or without incorporating a theoretical, empirical, and experience-based approach. | Research objectives are systematically derived from the needs of nursing practice, incorporating a theoretical, empirical, and experience-based approach, involving stakeholders from nursing practice, and are regularly reviewed. | Research goals are fully and continuously aligned with the needs of nursing practice, based on a theoretical, empirical, and experience-based approach involving stakeholders from nursing practice, and are proactively adjusted. | Not applicable | AI R&D<br>Nursing Science<br>Clinical Partner | 3 |
| **2.32** | **Practical benefit and added value of the AI system** | The practical benefit or added value of the AI system is unknown, or no clear practical benefit or added value is evident.<br><br>The AINC project is predominantly theoretical. | The potential benefit of the AI system is recognised, but concrete indicators or criteria for capturing the benefit and added value are unclear.<br><br>Initial steps to evaluate the added value are being taken but are not consistently recorded and documented. | The AI system shows a recognizable practical benefit, which is represented by concrete indicators or criteria.<br><br>However, endpoints are only partially captured, or the benefit is only partially realized. | The practical benefit of the AI system is clearly defined and is systematically implemented and demonstrated within the AINC project. | The AI system generates significant practical benefit and added value, which is continuously measured and optimized using clearly defined criteria and indicators. | Not applicable | AI R&D<br>Nursing Science<br>Clinical Partner | 3,5 |
| **2.33** | **Focus on realistic, field-tested projects over "grand vision" projects** | AINC projects are mostly visionary but detached from reality. There are no field trials. | Initial steps are being taken to implement realistic, practice-oriented AINC projects alongside visionary approaches. | Practice-oriented AINC projects are being conducted, but they are not yet fully tested. | AINC projects are both practice-oriented and visionary, with regular field trials for validation. | There is a balanced mix of practice-oriented, field-tested, and visionary AINC projects, optimally aligned with each other. | Not applicable | Nursing Science<br>AI R&D Clinical Partner | 3,5 |

| Dimension 2<br>Processual and Translational Requirements and Aspects | | | | | | | | | |
|---|---|---|---|---|---|---|---|---|---|
| **Attribute Number** | **Subdimension/ Attribute** | **Level 1 (initial)** | **Level 2 (assessing)** | **Level 3 (determined)** | **Level 4 (managed)** | **Level 5 (optimised)** | **Not applicable** | **(Joint) Assessment by (Rater Entity)** | **Source** |
| **2.34** | **Strategies for stakeholder participation and communication** | The strategies for participation and information of stakeholders in the AINC project are unknown or there are no clear strategies. | Initial steps are being taken to involve stakeholders in the AINC project, but without a systematic approach. | Stakeholders are actively involved in the AINC project, but not consistently (e.g., only once or in small numbers) or not comprehensively informed. | There are clear and structured strategies for comprehensive and repeated information and participation of stakeholders in the AI nursing project. | Strategies for stakeholder participation are fully integrated and optimised in the AINC project, with regular adjustments to new requirements (e.g., expanding the group of involved persons or implementing formats for participation and information that were not originally planned). | Not applicable | Nursing Science AI R&D Clinical Partner | 5 |
| **2.35** | **Strategies for trust-building, expectation alignment, and reducing concerns** | Strategies for building trust or reducing reservations towards AI systems in the AINC project are unknown or there are no clear strategies. | Initial approaches to building trust and aligning expectations are being developed but are not systematically and consistently implemented. | Trust-building and expectation management are part of the project planning but are not yet fully systematised and integrated (e.g., responsibility assigned only to one project partner). | Strategies for building trust and reducing reservations in the AINC project are established, documented, and systematically implemented. | Fully integrated and optimised strategies for building trust are implemented, with continuous adjustment and improvement throughout the project. | Not applicable | Nursing Science AI R&D Clinical Partner | 6 |
| **2.36** | **Reflection on the importance of involving humans as mediators between AI systems and actions, and any resulting development implications** | There is no reflection on the role of humans as an intermediary between AI systems and actions. | Initial considerations on the role of humans in interaction with AI systems exist, with internal project discussions, but no concrete measures are reflected in project implementation. | The role of humans as an intermediary is recognised but only partially translated into measures. Accordingly, its influence on AI system development is limited. | The importance of human mediation in interaction with AI is comprehensively reflected and systematically integrated into project implementation and AI system development. | Human mediation is regarded as a central component of AI implementation and is continuously optimised. | Not applicable | Nursing Science AI R&D Clinical Partner | 5 |
| **2.37** | **Strategies for long-term external support and evaluation of AI deployment** | There are no long-term strategies for external monitoring or evaluation of AI deployment, or there are considerations for such strategies but they are not yet designed for the long term. | Initial considerations for long-term external monitoring and evaluation exist, but no systematic planning or concretisation has been done yet. | External monitoring and evaluation take place partially, but are not yet continuous and consistent. | Long-term external monitoring and evaluation are established and regularly conducted. | External monitoring and evaluation are fully integrated, systematic, and optimised, with continuous adaptation. | Not applicable | AI R&D Nursing Science Clinical Partner | 2 |
| **2.38** | **Strategies for long-term external support: software/hardware: updates** | No clear strategies for software/hardware updates of the AI system exist. | Initial considerations for developing an update strategy exist but have not yet been or only very limitedly implemented. | Strategies for software/hardware updates are defined and partially implemented but not consistently optimised. | Comprehensive strategies for regular software/hardware updates exist, though there is still room for improvement. | Fully integrated and optimised strategies for software/hardware updates, which are continuously reviewed and adapted. | Not applicable | AI R&D | 2 |

ProKIP

Gefördert durch:
Bundesministerium für Forschung, Technologie und Raumfahrt

| Dimension 2<br>Processual and Translational Requirements and Aspects | | | | | | | | | |
|---|---|---|---|---|---|---|---|---|---|
| **Attribute Number** | **Subdimension/ Attribute** | **Level 1 (initial)** | **Level 2 (assessing)** | **Level 3 (determined)** | **Level 4 (managed)** | **Level 5 (optimised)** | **Not applicable** | **(Joint) Assessment by (Rater Entity)** | **Source** |
| **2.39** | **Strategies for long-term external support: software/hardware: upgrades** | No clear strategies for software/hardware upgrades of the AI system exist. | Initial considerations for developing an upgrade strategy exist but have not yet been or only very limitedly implemented. | Strategies for software/hardware upgrades are defined and partially implemented but not consistently optimised. | Comprehensive strategies for regular software/hardware upgrades exist, though there is still room for improvement. | Fully integrated and optimised strategies for software/hardware upgrades, which are continuously reviewed and adapted. | Not applicable | AI R&D | 3 |
| **2.40** | **Strategies for long-term external support: software/hardware: maintenance** | No clear strategies for maintenance of software and hardware of the AI system exist. | Initial approaches to develop maintenance strategies for software and hardware exist but are not yet fully implemented. | Strategies for maintenance of software and hardware are partially implemented but not consistently optimised. | Comprehensive strategies for regular maintenance of software and hardware exist, though there is still room for improvement. | Fully integrated and optimised strategies for maintenance of software and hardware, which are continuously reviewed and adapted. | Not applicable | AI R&D | 3 |

| Dimension 3<br>Technical Requirements and Aspects | | | | | | | | | |
|---|---|---|---|---|---|---|---|---|---|
| **Attribute Number** | **Subdimension/ Attribute** | **Level 1 (initial)** | **Level 2 (assessing)** | **Level 3 (determined)** | **Level 4 (managed)** | **Level 5 (optimised)** | **Not applicable** | **(Joint) Assessment by (Rater Entity)** | **Source** |
| **3.1** | **Integration into Existing Data Infrastructures and Platforms** | No integration of the AI system with existing data infrastructures or platforms, such as the European Health Data Space (EHDS). | Initial steps toward connecting the AI system to data infrastructures are being considered but not yet widely implemented. | Partial integration with existing data infrastructures, though there are still issues with interoperability. | Comprehensive integration with existing data infrastructures, which are largely well integrated. | Fully optimised integration with data infrastructures that operate seamlessly and efficiently and are regularly reviewed. | Not applicable | AI R&D | 2 |
| **3.2** | **Use of Technical Interoperability Standards and Nomenclatures** | No use of interoperability standards or nomenclatures. | Initial steps toward using and considering interoperability standards and nomenclatures in the development of the AI system within the AINC project, but not yet fully implemented. | Interoperability standards and nomenclatures are partially considered and used in the development of the AI system within the AINC project, but not consistently. | Extensive use and integration of interoperability standards and nomenclatures in the development of the AI system within the AINC project. | Complete and optimised use of interoperability standards and nomenclatures in the development of the AI system within the AINC project, regularly updated and applied across all relevant areas. | Not applicable | AI R&D<br>Nursing Science | 2,3,5 |
| **3.3** | **IT Security: Protection of Critical Infrastructure and Encryption Technologies (at the Clinical Partner)** | No specific measures in place for infrastructure protection or encryption technologies [at the clinical partner]. | Initial IT security measures and encryption techniques are being implemented [at the clinical partner], but gaps remain. | IT security practices and encryption technologies are partially established, but there is still room for improvement. | Comprehensive IT security measures and state-of-the-art encryption technologies are implemented and regularly reviewed. | Fully integrated, cutting-edge IT security infrastructure that is continuously optimised and monitored. | Not applicable | AI R&D<br>Clinical Partner | 3,5 |
| **3.4** | **IT Security: Security Certifications** | No security certifications available. | Initial steps toward obtaining security certifications are underway. | Some security certifications have been achieved, but not all relevant areas are covered. | Comprehensive security certifications are in place and regularly updated. | Fully optimised and up-to-date security certifications covering all relevant areas. | Not applicable | AI R&D | 5,6 |
| **3.5** | **Clinical Partner: Digital Infrastructure: Technical Infrastructure** | The technical infrastructure [at the clinical partner] is unknown or inadequate for AINC projects. | Initial steps have been taken to improve the technical infrastructure [at the clinical partner], but implementation is still lacking | A basic technical infrastructure for AINC projects exists [at the clinical partner], but there are still bottlenecks and limitations. | The technical infrastructure [at the clinical partner] is well developed and effectively supports most AI applications. | The technical infrastructure [at the clinical partner] is state-of-the-art, fully integrated, and optimised for AI use. | Not applicable | AI R&D | 2,3,5 |
| **3.6** | **IT Infrastructure: AI Compute: Hardware** | No dedicated AI hardware available. | Dedicated AI hardware is available but not yet connected, e.g., to data interfaces. | Dedicated AI hardware is available and connected (e.g., to data interfaces). It is possible to transfer data into the runtime environment for model development. | Dedicated AI hardware is available and connected. Standardised interfaces are in place for data transfer and for offering model predictions. Continuous testing and maintenance of the AI system is possible. | Fully optimised and up-to-date dedicated (hardware and software) runtime environments are available. Model development and maintenance are implemented according to modern CI/CD components. | Not applicable | AI R&D | PROKIP |

ProKIP

Gefördert durch:
Bundesministerium für Forschung, Technologie und Raumfahrt

| Dimension 4<br>Social and Ethical Requirements and Aspects | | | | | | | | | |
|---|---|---|---|---|---|---|---|---|---|
| **Attribute Number** | **Subdimension/ Attribute** | **Level 1 (initial)** | **Level 2 (assessing)** | **Level 3 (determined)** | **Level 4 (managed)** | **Level 5 (optimised)** | **Not applicable** | **(Joint) Assessment by (Rater Entity)** | **Source** |
| **4.1** | **Ethics Vote** | No ethics vote has been prepared or is planned.<br><br>It is unclear whether one is needed or what kind. | Initial steps to obtain an ethics vote have been taken but are incomplete, or responsibilities are not coordinated within the project consortium.<br><br>No final ethics vote has been obtained. | Ethics votes are obtained for some project steps but are not systematically coordinated, causing redundancy and additional effort. | A positive ethics vote exists for all relevant project steps and is renewed when needed.<br><br>Responsibilities and procedures for amendments are clear and known amongst all project partners. | A fully integrated, planned, and optimised process for obtaining and updating ethics votes exists across the entire AINC project. | Not applicable | AI R&D<br>Nursing Science | 2 |
| **4.2** | **Informed and Ongoing Consent (Including Alternatives)** | No strategies or considerations for informed or ongoing consent for the AINC project exist or are known.<br><br>It's unclear if or how ongoing consent should be implemented in the AINC project. | Initial ideas to obtain informed or ongoing consent or alternative approaches are being developed but are not yet fully defined or implemented. | Informed Consent or Ongoing Consent are mostly implemented, but gaps remain (e.g., not present in all project parts or phases where it would be necessary). | Comprehensive strategies for informed or ongoing consent, including alternatives, are in place and regularly reviewed | Fully optimised and flexible strategies for informed or ongoing exist, continuously adapted and developed further. | Not applicable | AI R&D<br>Nursing Science | 2 |
| **4.3** | **Centralised and Decentralised Methods for and Limitations of Ensuring Study Participant* Privacy**<br>*Study Participants are all individuals from whom data is collected in the AINC project to develop or evaluate an AI system. This also includes nurses and other health professionals, for example, those who participate in focus groups. | No methods for ensuring participant privacy in the AINC project exist or methods are unknown. | Initial methods for ensuring participant privacy are being developed but are not yet comprehensive. | Central and decentralised methods for ensuring participant privacy are partly implemented but not optimised. | Comprehensive and regularly reviewed methods for ensuring participant privacy are in place and recurringly revised. | Fully integrated, optimised, and regularly updated methods for ensuring participant privacy are implemented. | Not applicable | AI R&D<br>Nursing Science | 2 |
| **4.4** | **Engagement with Ethical-Normative Values of Nursing and Care, and Individual Clinical Partners** | No engagement with ethical values (has taken place) in the AINC project. | Initial ideas on how to engage with ethical-normative values in the AINC project exist but are not concrete or systematic. | Methods to engage with ethical-normative values are defined but only partially implemented or exclude some stakeholders. | Regular, systematic, and documented engagement with ethical-normative values and all stakeholders takes place in the AINC project. | Regular, systematic, and documented engagement with ethical-normative values and all stakeholders takes place in the AINC project, and is adapted as needed. | Not applicable | AI R&D<br>Nursing Science<br>Clinical Partner | 2,5,6 |

| Dimension 4<br>Social and Ethical Requirements and Aspects | | | | | | | | | |
|---|---|---|---|---|---|---|---|---|---|
| **Attribute Number** | **Subdimension/ Attribute** | **Level 1 (initial)** | **Level 2 (assessing)** | **Level 3 (determined)** | **Level 4 (managed)** | **Level 5 (optimised)** | **Not applicable** | **(Joint) Assessment by (Rater Entity)** | **Source** |
| **4.5** | **Reflection on Impact of the AI System on the Nursing Work Environment** | No reflection on the impact of AI on the work environment has taken place. | Initial considerations on how a reflection of the impact of AI on the nursing work environment will take place in the AINC project exist but are not systematic or implemented. | Impact of AI on the nursing work environment takes place in the AINC project but partial and unsystematic reflection takes place. | Comprehensive, systematic reflection of the impact of AI on the nursing work environment is part of the AINC project. | Comprehensive, fully systematic reflection of the impact of AI on the nursing work environment for all stakeholders is part of the AINC project and is adapted as needed. | Not applicable | AI R&D<br>Nursing Science<br>Clinical Partner | 2 |
| **4.6** | **Reflection on the Impact of AI on the Nursing Profession** | The general influence of AI on Nursing is acknowledged, but there is no project-level reflection. | Initial reflections on the impact of AI on the nursing profession are present.<br><br>However, reflections are unsystematic and without an effect on system development, implementation or evaluation. | Systematic reflection on the impact of AI on the nursing profession is part of the AINC project but lacks influence on development, implementation or evaluation. | Comprehensive reflection on the impact of AI on the nursing profession that informs system development, implementation and evaluation takes place in the AINC project. | Fully integrated reflection on the impact of AI on the nursing profession included in AINC project results and documentation | Not applicable | AI R&D<br>Nursing Science<br>Clinical Partner | 2 |
| **4.7** | **Strategies for Systematic Assessment of Intended and Unintended Effects of AI** | No strategies for or discussions on systematic assessment of intended and unintended effects of AI are in place in the AINC project. | Initial strategies for or discussions on systematic assessment of intended and unintended effects of AI in the AINC project exist. | Initial strategies for a systematic assessment of intended and unintended effects of AI in the AINC project developed but not fully integrated or systematically implemented. | Fully integrated and systematically implemented strategies for a systematic assessment of intended and unintended effects of AI in the AINC project exist. | Fully integrated and systematically implemented strategies for a systematic assessment of intended and unintended effects of AI in the AINC project exist and are adapted continuously during the project based on need. | Not applicable | AI R&D<br>Nursing Science | 2 |
| **4.8** | **Reflection on Data Representativeness and Transferability of Results of the AINC Project** | No (not yet) reflection on data representativeness and result transferability in the AINC project. | Initial reflections on data representativeness or result transferability in the AINC project exist but are undocumented | Reflections on data representativeness and result transferability in the AINC project exist, but documentation is partial with gaps. | Comprehensive reflection on data representativeness and result transferability in the AINC project exists and is regularly reviewed and documented. | Fully integrated, regularly reviewed, and continuously updated and documented reflection on data representativeness and result transferability in the AINC project. | Not applicable | AI R&D<br>Nursing Science | 2 |

| Dimension 4<br>Social and Ethical Requirements and Aspects | | | | | | | | | |
|---|---|---|---|---|---|---|---|---|---|
| **Attribute Number** | **Subdimension/ Attribute** | **Level 1 (initial)** | **Level 2 (assessing)** | **Level 3 (determined)** | **Level 4 (managed)** | **Level 5 (optimised)** | **Not applicable** | **(Joint) Assessment by (Rater Entity)** | **Source** |
| **4.9** | **Strategies to Increase Transparency and Explainability of AI Decisions** | No (not yet) strategies to increase transparency and explainability of AI decisions in the AINC project exist or are known. | Initial strategies to increase transparency and explainability of AI decisions in the AINC project are developed but not fully implemented. | Strategies to increase transparency and explainability of AI decisions in the AINC project are partially implemented but not optimised. | Optimised strategies to increase transparency and explainability of AI decisions in the AINC project are implemented and regularly reviewed. | Comprehensive, regularly reviewed, and continuously improved strategies to increase transparency and explainability of AI decisions in the AINC project exist. | Not applicable | AI R&D<br>Nursing Science | 2,5 |
| **4.10** | **Responsible Data Management: Individual Consent, Data Donation, Research Exemption or Data Trusteeship***<br>*When data are collected or used – for example, data from nursing documentation, app usage data, or movement data – this should be done responsibly. The aim is to protect individuals' privacy and handle their information fairly. There are various concepts for this:<br>Individual consent: People decide for themselves whether and which of their data may be used.<br>Data donation: People voluntarily provide their data for research purposes.<br>Research exemption: In certain cases, researchers may use data without direct consent, for example, when there is a particularly strong public interest and data protection is still ensured.<br>Example: Health data from a hospital is anonymised and used to study a pandemic.<br>Data trusteeship: A neutral third party (e.g., a foundation or public authority) manages the data and only releases it when certain rules are met. | No reflection on responsible data handling in the AINC project. | Initial but undocumented reflections on responsible data management in the AINC project. | Responsible data management is mostly systematically reflected on the AINC project but some gaps exist. | Comprehensive systematic reflection on responsible data management in the AINC project, regularly documented. | Fully optimised, documented, and continuously updated reflection on responsible data management in the AINC project. | Not applicable | AI R&D | 2 |

Gefördert durch:
Bundesministerium für Forschung, Technologie und Raumfahrt

| Dimension 4<br>Social and Ethical Requirements and Aspects | | | | | | | | | |
|---|---|---|---|---|---|---|---|---|---|
| **Attribute Number** | **Subdimension/ Attribute** | **Level 1 (initial)** | **Level 2 (assessing)** | **Level 3 (determined)** | **Level 4 (managed)** | **Level 5 (optimised)** | **Not applicable** | **(Joint) Assessment by (Rater Entity)** | **Source** |
| **4.11** | **Consideration of Values of Nursing and Positioning** | No reflection on nursing value orientation in the AINC project. | Initial but undocumented reflection on nursing value orientation in the AINC project. | Nursing value orientation is mostly systematically reflected on in the AINC project but some gaps exist. | Comprehensive systematic reflection on nursing value orientation in the AINC project, regularly documented. | Fully optimised, documented, and continuously updated reflection on nursing value orientation in the AINC project. | Not applicable | AI R&D<br>Nursing Science<br>Clinical Partner | PROKIP |

| 5<br>Community Building Requirements and Aspects | | | | | | | | | |
|---|---|---|---|---|---|---|---|---|---|
| **Attribute Number** | **Subdimension/ Attribute** | **Level 1 (initial)** | **Level 2 (assessing)** | **Level 3 (determined)** | **Level 4 (managed)** | **Level 5 (optimised)** | **Not applicable** | **(Joint) Assessment by (Rater Entity)** | **Source** |
| **5.1** | **Technological Knowledge Transfer** | The AINC project is not involved in technological knowledge transfer.<br><br>Technological knowledge transfer is not planned or possible.<br>Activities related to technological knowledge transfer are unknown. | Initial steps and explorations for technological knowledge transfer are being undertaken, but not yet systematically implemented. | Technological knowledge transfer is partially conducted, but there are still gaps in consistency (e.g., irregular participation, depending on the engagement of individual employees in the AINC project) and scope (e.g., only regional or addressing only selected but not all target groups). | Comprehensive technological knowledge transfer is established and occurs regularly.<br><br>The approach for technological knowledge transfer is regularly updated and reviewed.<br><br>There is at least national reach of the technological knowledge transfer. | Fully integrated and optimised technological knowledge transfer is taking place, which is continuously expanded and adapted to new findings of the AINC project and the AI system.<br><br>National and international reach of the technological knowledge transfer is ensured. | Not applicable | AI R&D<br>Nursing Science | 2 |
| **5.2** | **Participation in Online Exchange** | No participation of partners in the AINC project in online exchange through engagement in Q&A networks.<br><br>No involvement of programmers in a shared repository. | Initial steps are being taken to set up an online exchange in Q&A networks or to participate in a shared repository, but these are not yet comprehensively implemented. | Online exchange and participation in a shared repository are partially established, but there is still room for improvement (e.g., exchange and participation depend on the engagement of individual employees in the AINC project or participation is unsystematic). | Comprehensive online exchange and regular participation in Q&A networks and a shared repository are established and actively used by all suitable employees in the AINC project. | Fully integrated and optimised participation in online exchange and a shared repository, which is continuously expanded and adapted to the latest findings of the AINC project or AI system. | Not applicable | AI R&D<br>Nursing Science<br>Clinical Partner | 2 |
| **5.3** | **Strategic Partnerships** | Existing strategic partnerships are unknown or there are no strategic partnerships in the AINC project or among individual project partners. | Initial strategic partnerships are being explored, but have not yet been fully concluded or formalised. | Strategic partnerships are partially established, but not all partners in the AINC project have strategic partnerships.<br><br>Clinical partners in particular do not have strategic partnerships outside of the project consortium. | Comprehensive strategic partnerships are established.<br><br>Clinical partners also have strategic partnerships outside of the project consortium.<br><br>Existing strategic partnerships are regularly reviewed and expanded, especially on a national level. | Proven and effective strategic partnerships are established for all partners in the AINC project.<br><br>Existing strategic partnerships are continuously and internationally expanded. | Not applicable | AI R&D<br>Nursing Science<br>Clinical Partner | 6 |

## References

1. Pumplun, L., Fecho, M., Wahl, N., Peters, F. & Buxmann, P. Adoption of Machine Learning Systems for Medical Diagnostics in Clinics: Qualitative Interview Study. J Med Internet Res 23, e29301 (2021).
2. Wolf-Ostermann, K., et al. Konzept zur Einbettung von KI-Systemen in der Pflege: Sondierungsprojekt zu KI in der Pflege (SoKIP). (Universität Bremen, Bremen, 2021).
3. Alami, H., et al. Organizational readiness for artificial intelligence in health care: insights for decision-making and practice. J Health Organ Manag (2020).
4. Weinert, L., Müller, J., Svensson, L. & Heinze, O. Perspective of Information Technology Decision Makers on Factors Influencing Adoption and Implementation of Artificial Intelligence Technologies in 40 German Hospitals: Descriptive Analysis. JMIR Medical Informatics 10(2022).
5. Chang, A., Implementation of Artificial Intelligence in Medicine, in Intelligence-Based Medicine. Artificial Intelligence and Human Cognition in Clinical Medicine and Healthcare. 2020, Academic Press: London, San Diego, Cambridge, Oxford. p. 397-412.
6. Wiljer, D. & Hakim, Z. Developing an Artificial Intelligence–Enabled Health Care Practice: Rewiring Health Care Professions for Better Care. Journal of Medical Imaging and Radiation Sciences 50, S8-S14 (2019).
7. Abuzaid, M.M., Elshami, W., Tekin, H. & Issa, B. Assessment of the Willingness of Radiologists and Radiographers to Accept the Integration of Artificial Intelligence Into Radiology Practice. Acad Radiol 29, 87-94 (2022).
8. Andersson, J., et al. Artificial intelligence and the medical physics profession - A Swedish perspective. Phys Med 88, 218-225 (2021).

Gefördert durch:
Bundesministerium für Forschung, Technologie und Raumfahrt

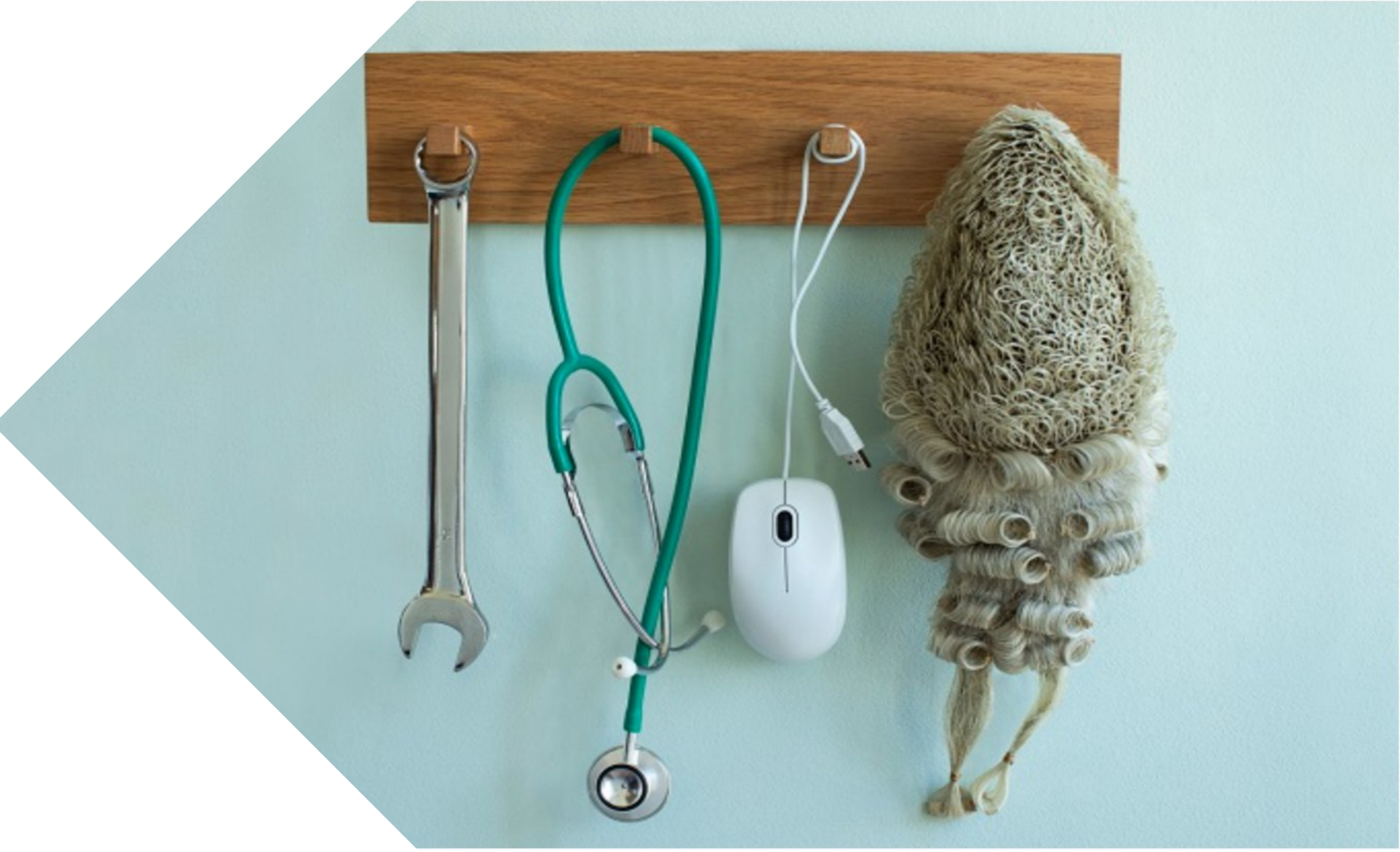

The scientific accompanying project „Process development and support for the use of AI in care" within the funding initiative "Making Repositories and AI Systems Usable in Everyday Nursing Care" is funded by the Federal Ministry of Research, Technology and Space (Funding Code: 16SV8835). The funding body had no influence on the study design, data collection, analysis, interpretation, or the writing of the manuscript.

**Authors:**
Kathrin Seibert, Dominik Domhoff, Janissa Altona, Sebastian Jäger, Felix Bießmann, Alessia Nowak, Rahel Gubser, Matthias Schulte-Althoff, Daniel Fürstenau, Jörg Pohle, Lea Bergmann, Kathi Beier, Dagmar Borchers, Karin Wolf-Ostermann
**With contributions from:** David Walter, Richard Dulzon

University of Bremen – Faculty 11 Human and Health Sciences – Institute for Public Health and Nursing Research – Grazer Str. 4, 28359 Bremen – https://www.uni-bremen.de/en/institut-fuer-public-health-und-pflegeforschung



Universität Bremen · vediso · BHT Berliner Hochschule für Technik · Einstein Center Digital Future · Alexander von Humboldt Institut für Internet und Gesellschaft · Charité Universitätsmedizin Berlin

Gefördert durch:
Bundesministerium für Forschung, Technologie und Raumfahrt

# KI-Pflege-Readiness-Assessment (KIP-RA)

## Instrument

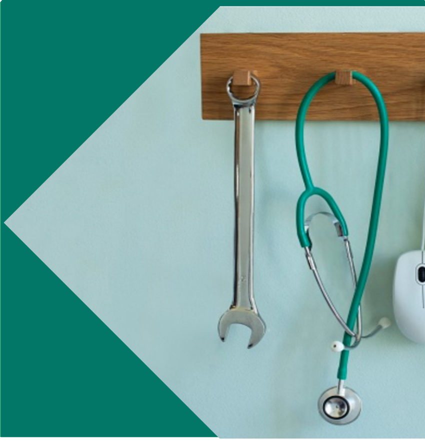

Ein Ergebnis der wissenschaftlichen Begleitforschung im BMBFTR-Förderprogramm „Repositorien und KI-Systeme im Pflegealltag nutzbar machen“

Juli 2025

Gefördert durch:
Bundesministerium für Forschung, Technologie und Raumfahrt

| Dimension 1<br>Regulatorische Voraussetzungen und Aspekte | | | | | | | | | |
|---|---|---|---|---|---|---|---|---|---|
| **Attribut-Nummer** | **Attribute** | **Stufe 1 (initial)** | **Stufe 2 (erkundend)** | **Stufe 3 (entschlossen)** | **Stufe 4 (gesteuert)** | **Stufe 5 (optimiert)** | **Trifft nicht zu** | **Bewertung durch** | **Quelle** |
| **1.1** | Analyse des Datenbestandes: Informationsgehalt (trotz Anonymisierung) | Der Informationsgehalt der für das KIP-Projekt benötigten Daten ist unbekannt oder die Daten liegen als nicht anonymisierte personenbezogene Daten vor. | Die für das KIP-Projekt benötigten Daten liegen als anonymisierte Daten, mit fast vollständigem Verlust des diagnostisch relevanten Informationswerts vor. | Die für das KIP-Projekt benötigten Daten liegen als anonymisierte Daten, mit niedrigem Niveau des diagnostisch relevanten Informationswerts vor. | Die für das KIP-Projekt benötigten Daten liegen als anonymisierte Daten, mit mittlerem Niveau des diagnostisch relevanten Informationswerts vor. | Die für das KIP-Projekt benötigten Daten liegen als anonymisierte Daten, mit erhaltenem diagnostisch relevantem Informationswert vor. | Trifft nicht zu. | KI F&E<br>Pflege-wissenschaft<br>Praxispartner | 1 |
| **1.2** | Analyse des Datenbestandes: Repräsentativität von Trainingsdaten | Unbekannte Übereinstimmung oder fast keine Übereinstimmung zwischen den Daten und dem vorgesehenen Anwendungskontext des KI-Systems (z.B. Demografie, Region oder Arbeitskultur) in den Trainingsdaten des KIP-Projektes. | Geringe Übereinstimmung zwischen den Daten und dem vorgesehenen Anwendungskontext des KI-Systems (z.B. Demografie, Region oder Arbeitskultur) in den Trainingsdaten des KIP-Projektes. | Mittlere Übereinstimmung zwischen den Daten und dem vorgesehenen Anwendungskontext des KI-Systems (z.B. Demografie, Region oder Arbeitskultur) in den Trainingsdaten des KIP-Projektes. | Hohe Übereinstimmung zwischen den Daten und dem vorgesehenen Anwendungskontext des KI-Systems (z.B. Demografie, Region oder Arbeitskultur) in den Trainingsdaten des KIP-Projektes. | Vollständige Übereinstimmung zwischen den Daten und dem vorgesehenen Anwendungskontext des KI-Systems (z.B. Demografie, Region oder Arbeitskultur) in den Trainingsdaten des KIP-Projektes. | Trifft nicht zu. | KI F&E<br>Pflege-wissenschaft | 1,2 |
| **1.3** | Analyse des Datenbestandes: Qualität | Die Datenqualität ist unbekannt oder keine einheitliche Datenqualität (z. B. handschriftliche, synonymbasierte Diagnosen) in den für das KIP-Projekt benötigten Daten [beim Praxispartner]. | Erste Forderungen und Überlegungen nach Vereinheitlichung der Datenqualität [des Praxispartners] im KIP-Projekt oder Durchführung eines Datenqualitäts-Assessments (z.B. Pandas Profile). | Betriebsinterne Qualitätsstandards (z. B. für Schlüsselterminologien) [beim Praxispartner] im KIP-Projekt. | Betriebsübergreifende Qualitätsstandards (z. B. für Schlüsselterminologien) [beim Praxispartner] im KIP-Projekt. | Internationale Qualitätsstandards (z.B. Unified Medical Language System (UMLS)) [beim Praxispartner] im KIP-Projekt umgesetzt. | Trifft nicht zu. | KI F&E<br>Praxispartner | 1,2 |

ProKIP

Gefördert durch: Bundesministerium für Forschung, Technologie und Raumfahrt

| Dimension 1<br>Regulatorische Voraussetzungen und Aspekte | | | | | | | | | |
|---|---|---|---|---|---|---|---|---|---|
| **Attribut-Nummer** | **Attribute** | **Stufe 1 (initial)** | **Stufe 2 (erkundend)** | **Stufe 3 (entschlossen)** | **Stufe 4 (gesteuert)** | **Stufe 5 (optimiert)** | **Trifft nicht zu** | **Bewertung durch** | **Quelle** |
| **1.4** | Analyse des Datenbestandes: Verfügbarkeit | Für das KIP-Projekt benötigte Daten sind [beim Praxispartner] kaum oder gar nicht digital verfügbar. | Erste Bemühungen zur zentralen digitalen Erfassung und Bereitstellung von für das KIP-Projekt benötigten Daten [beim Praxispartner]. Datenverfügbarkeit ist jedoch sporadisch und oft unvollständig. Es gibt grundlegende Speichersysteme, aber die Daten sind häufig veraltet oder nur für bestimmte Bereiche verfügbar. | Für das KIP-Projekt benötigte Daten sind [beim Praxispartner] in zentralen Systemen gespeichert und stehen für mehrere Abteilungen digital zur Verfügung. Die Datenverfügbarkeit ist konsistenter, aber es gibt noch gelegentliche Engpässe. Die Datenaktualität wird regelmäßig überprüft, jedoch nicht immer in Echtzeit gewährleistet. | Für das KIP-Projekt benötigte digitale Daten sind durchgängig im Betrieb [beim Praxispartner] verfügbar und werden zentral und automatisiert verwaltet. Die Datenverfügbarkeit ist hoch und es gibt klare Prozesse für das Datenmanagement. Die meisten Daten sind in Echtzeit zugänglich und werden regelmäßig aktualisiert, um Betriebsanforderungen zu erfüllen. | Für das KIP-Projekt benötigte digitale Daten sind jederzeit und in Echtzeit im gesamten Betrieb [des Praxispartners] verfügbar. Die Verfügbarkeit ist durch Redundanz und fortschrittliche Technologien sichergestellt. Datenmanagementprozesse sind vollständig optimiert und die Datenverfügbarkeit unterstützt proaktiv betriebliche Entscheidungen und strategische Ziele. | Trifft nicht zu. | KI F&E Praxispartner | 1-4 |
| **1.5** | **Analyse des Datenbestandes: Zugang** | Der Zugangsweg zu den für das KIP-Projekt benötigten Daten ist unbekannt oder es ist kein systematischer Zugang zu den Daten [beim Praxispartner] vorhanden. Daten werden entweder nicht erfasst oder sind nur lokal und in unstrukturierter Form vorhanden. Der Zugriff auf Daten erfolgt sporadisch und manuell, oft nur auf Anforderung. | Erste Schritte zur Erfassung und Sammlung von für das KIP-Projekt benötigten Daten [beim Praxispartner] werden unternommen. Der Zugang ist jedoch begrenzt und oft auf bestimmte Abteilungen oder Systeme beschränkt. Daten sind teilweise veraltet und der Zugriff ist unzuverlässig und ineffizient. | Systematischer Ansatz zur Datenerfassung und -speicherung der für das KIP-Projekt benötigten Daten [beim Praxispartner] wird verfolgt.<br>Der Zugang zu Betriebsdaten ist für relevante Nutzendengruppen möglich, jedoch noch nicht durchgängig in Echtzeit. Daten sind teilweise integriert und es gibt festgelegte Prozesse für den Zugriff. | Der Zugang zu den für das KIP-Projekt benötigten Daten [beim Praxispartner] ist durchgängig verfügbar und weitgehend automatisiert.<br>Die Daten sind aktuell, konsistent und in Echtzeit zugänglich.<br>Ein zentralisiertes System ermöglicht den kontrollierten Zugriff auf Daten über verschiedene Abteilungen hinweg. | Der Zugang zu den für das KIP-Projekt benötigten Daten [beim Praxispartner] ist vollständig integriert und optimiert. Daten sind jederzeit und für alle berechtigten Nutzenden in Echtzeit verfügbar. Fortgeschrittene Analysewerkzeuge und automatisierte Prozesse sorgen für einen effizienten und sicheren Zugang, der kontinuierlich optimiert wird. | Trifft nicht zu. | KI F&E Praxispartner | 2,3,5 |

| Dimension 1<br>Regulatorische Voraussetzungen und Aspekte | | | | | | | | | |
|---|---|---|---|---|---|---|---|---|---|
| **Attribut-Nummer** | **Attribute** | **Stufe 1 (initial)** | **Stufe 2 (erkundend)** | **Stufe 3 (entschlossen)** | **Stufe 4 (gesteuert)** | **Stufe 5 (optimiert)** | **Trifft nicht zu** | **Bewertung durch** | **Quelle** |
| **1.6** | Modelle des Datenteilens | Für das KIP-Projekt relevante Modelle des Datenteilens sind unbekannt oder unklar. Es gibt keine Überlegungen zu geeigneten Modellen und Vorgehensweisen. | Erste Überlegungen und Schritte zur Festlegung und Umsetzung des Modells zum Teilen von Daten werden unternommen. Das Modell ist jedoch nicht festgelegt und notwendige einzubindende Personen und Vorgehensweisen sind nicht hinreichend geklärt. | Das Modell des Datenteilens und die Vorgehensweise sind festgelegt, die zu erfüllenden rechtlichen, organisatorischen und technischen Anforderungen sind weitgehend identifiziert. Kontaktpersonen und Verantwortlichkeiten für die Umsetzung sind bekannt, aber mit Aushandlung der Details und der Umsetzung wurde noch nicht begonnen. | Das Modell des Datenteilens und die Vorgehensweise sind festgelegt und allen Projektbeteiligten bekannt. Die zu erfüllenden rechtlichen, organisatorischen und technischen Anforderungen sind vollständig identifiziert und liegen in systematisch-strukturierter Weise vor. Verantwortliche Kontaktpersonen sind in das KIP-Projekt eingebunden und haben an der Aushandlung der Details teilgenommen. Die Umsetzung des Datenteilens findet mit entsprechenden regulatorischen, organisatorischen und technischen Vorkehrungen statt. | Das Modell und die Prozesse des Datenteilens sind mit allen erforderlichen regulatorischen, organisatorischen und technischen Vorkehrungen umgesetzt. Die Erfüllung der Anforderungen wird nachgewiesen, z.B. durch Selbst- oder externe Zertifizierung. Die Umsetzung – das praktizierte Teilen und die Nutzung der geteilten Daten – ist vollständig und verständlich dokumentiert, die Dokumentation wird regelmäßig überprüft, die Strukturen und Prozesse werden entsprechend der Prüfergebnisse nachvollziehbar weiterentwickelt. | Trifft nicht zu. | KI F&E | 2,3,5 |

| Dimension 1<br>Regulatorische Voraussetzungen und Aspekte | | | | | | | | | |
|---|---|---|---|---|---|---|---|---|---|
| **Attribut-Nummer** | **Attribute** | **Stufe 1 (initial)** | **Stufe 2 (erkundend)** | **Stufe 3 (entschlossen)** | **Stufe 4 (gesteuert)** | **Stufe 5 (optimiert)** | **Trifft nicht zu** | **Bewertung durch** | **Quelle** |
| **1.7** | EU-Medizinprodukte-Verordnung EU-MPV (und/oder, wenn auch auslaufend, MPG) | Keine Berücksichtigung von EU-MPV oder MPG in dem KIP-Projekt oder für das KIP-Projekt relevante Inhalte von EU-MPV oder MPG sind unbekannt. | Erste Schritte zur Einhaltung der EU-MPV oder anderer relevanter Medizinproduktregelungen in dem KIP-Projekt, grundlegende Übersicht über die entsprechenden rechtlichen Anforderungen vorhanden, die Anforderungen sind aber nur teilweise, unvollständig, unsystematisch bzw. uneinheitlich umgesetzt. Die Verantwortlichkeiten im Projekt (z.B. die Rolle des "Sponsors" in klinischen Studien) sowie notwendige einzubindende Personen oder Stellen außerhalb des Projekts sowie Vorgehensweisen sind nicht abschließend geklärt. | Das KIP-Projekt hat sich auf die umfassende Einhaltung der EU-MPV oder anderer relevanter Medizinproduktregelungen verständigt, sich eine weitgehende Übersicht über die entsprechenden rechtlichen Anforderungen verschafft sowie Verantwortlichkeiten im Projekt festgelegt. Viele Anforderungen sind umgesetzt bzw. zur Umsetzung geplant, aber die Vollständigkeit der Abdeckung ist nicht gegeben, die Umsetzungen sind allenfalls teilweise dokumentiert, getestet und evaluiert. | Das KIP-Projekt hat sich auf die umfassende Einhaltung der EU-MPV oder anderer relevanter Medizinproduktregelungen verständigt sowie sich eine umfassende und strukturierte Übersicht über die entsprechenden rechtlichen Anforderungen verschafft, und die Projektbeteiligten darüber informiert. Die Verantwortlichkeiten im Projekt sind festgelegt, und die Verantwortlichen sind in das Projekt eingebunden. Das Vorgehen bei der Umsetzung folgt einem definierten Standard. Alle Anforderungen sind umgesetzt bzw. mit konkreten Deadlines und entsprechenden Ressourcen (Zeit, Personal, sonstige) zur Umsetzung geplant. Die Vollständigkeit der Abdeckung ist sichergestellt, die Umsetzungen sind umfassend dokumentiert, getestet und evaluiert, und die Prozesse und Ergebnisse werden regelmäßig überwacht und aktualisiert. | Das KIP-Projekt hat sich auf die umfassende Einhaltung der EU-MPV oder anderer relevanter Medizinproduktregelungen verständigt sowie sich eine vollständige und systematisch strukturierte Übersicht über die entsprechenden rechtlichen Anforderungen verschafft und allen Projektbeteiligten bekannt gemacht. Die Verantwortlichkeiten im Projekt sind festgelegt, und die Verantwortlichen sind in das Projekt eingebunden. Das Vorgehen bei der Umsetzung folgt einem definierten nationalem oder internationalem Standard, etwa ISO 13485. Alle Anforderungen sind umgesetzt bzw. mit konkreten Deadlines und entsprechenden Ressourcen (Zeit, Personal, sonstige) zur Umsetzung geplant. Die Vollständigkeit der Abdeckung ist nachweisbar sichergestellt, die Umsetzungen sind vollständig dokumentiert, getestet und evaluiert sowie extern zertifiziert, und die Prozesse und Ergebnisse werden regelmäßig überwacht und aktualisiert. | Trifft nicht zu. | KI F&E<br>Pflege-wissenschaft<br>Praxispartner | 2,5,6 |

| Dimension 1<br>Regulatory Requirements and Aspects | | | | | | | | | |
|---|---|---|---|---|---|---|---|---|---|
| **Attribut-Nummer** | **Attribute** | **Stufe 1 (initial)** | **Stufe 2 (erkundend)** | **Stufe 3 (entschlossen)** | **Stufe 4 (gesteuert)** | **Stufe 5 (optimiert)** | **Trifft nicht zu** | **Bewertung durch** | **Quelle** |
| **1.8** | EU-DSGVO und Spezialgesetze | Keine Berücksichtigung der EU-DSGVO oder anderer relevanter Datenschutzregelungen in dem KIP-Projekt. Keine Kenntnisse vorhanden, welche Projektteile datenschutzrechtsrelevant sind. | Erste Schritte zur Einhaltung der EU-DSGVO oder anderer relevanter Datenschutzregelungen in dem KIP-Projekt, grundlegende Übersicht über die entsprechenden rechtlichen Anforderungen vorhanden, die Anforderungen sind aber nur teilweise, unvollständig, unsystematisch bzw. uneinheitlich umgesetzt. Die Verantwortlichkeiten im Projekt sowie notwendige einzubindende Personen oder Stellen außerhalb des Projekts sowie Vorgehensweisen sind nicht abschließend geklärt. | Das KIP-Projekt hat sich auf die umfassende Einhaltung der EU-DSGVO oder anderer relevanter Datenschutzregelungen verständigt, sich eine weitgehende Übersicht über die entsprechenden rechtlichen Anforderungen verschafft sowie Verantwortlichkeiten im Projekt festgelegt. Viele Anforderungen sind umgesetzt bzw. zur Umsetzung geplant, aber die Vollständigkeit der Abdeckung ist nicht gegeben, die Umsetzungen sind allenfalls teilweise dokumentiert, getestet und evaluiert. | Das KIP-Projekt hat sich auf die umfassende Einhaltung der EU-DSGVO oder anderer relevanter Datenschutzregelungen verständigt sowie sich eine umfassende und strukturierte Übersicht über die entsprechenden rechtlichen Anforderungen verschafft, und die Projektbeteiligten darüber informiert. Die Verantwortlichkeiten im Projekt sind festgelegt, und die Verantwortlichen sind in das Projekt eingebunden. Das Vorgehen bei der Umsetzung folgt einem definierten Standard. Alle Anforderungen sind umgesetzt bzw. mit konkreten Deadlines und entsprechenden Ressourcen (Zeit, Personal, sonstige) zur Umsetzung geplant. Die Vollständigkeit der Abdeckung ist sichergestellt, die Umsetzungen sind umfassend dokumentiert, getestet und evaluiert, und die Prozesse und Ergebnisse werden regelmäßig überwacht und aktualisiert. | Das KIP-Projekt hat sich auf die umfassende Einhaltung der EU-DSGVO oder anderer relevanter Datenschutzregelungen verständigt sowie sich eine vollständige und systematisch strukturierte Übersicht über die entsprechenden rechtlichen Anforderungen verschafft und allen Projektbeteiligten bekannt gemacht. Die Verantwortlichkeiten im Projekt sind festgelegt, und die Verantwortlichen sind in das Projekt eingebunden. Das Vorgehen bei der Umsetzung folgt einem definierten nationalem oder internationalem Standard, etwa dem Standard-Datenschutzmodell (SDM). Alle Anforderungen sind umgesetzt bzw. mit konkreten Deadlines und entsprechenden Ressourcen (Zeit, Personal, sonstige) zur Umsetzung geplant. Die Vollständigkeit der Abdeckung ist nachweisbar sichergestellt, die Umsetzungen sind vollständig dokumentiert, getestet und evaluiert sowie extern zertifiziert, und die Prozesse und Ergebnisse werden regelmäßig überwacht und aktualisiert. | Trifft nicht zu. | KI F&E<br>Pflege-wissenschaft<br>Praxispartner | 2,5,6 |
| Dimension 1<br>Regulatory Requirements and Aspects | | | | | | | | | |

| Attribut-Nummer | Attribute | Stufe 1 (initial) | Stufe 2 (erkundend) | Stufe 3 (entschlossen) | Stufe 4 (gesteuert) | Stufe 5 (optimiert) | Trifft nicht zu | Bewertung durch | Quelle |
|---|---|---|---|---|---|---|---|---|---|
| **1.9** | EU-KI-Verordnung | Keine Berücksichtigung der EU-KI-Verordnung in dem KIP-Projekt. Keine Kenntnisse vorhanden, welche Projektteile KI-verordnungsrelevant sind. | Erste Schritte zur Einhaltung der EU-KI-Verordnung in dem KIP-Projekt, grundlegende Übersicht über die entsprechenden rechtlichen Anforderungen vorhanden, die Anforderungen sind aber nur teilweise, unvollständig, unsystematisch bzw. uneinheitlich umgesetzt. Die Verantwortlichkeiten im Projekt sowie notwendige einzubindende Personen oder Stellen außerhalb des Projekts sowie Vorgehensweisen sind nicht abschließend geklärt. | Das KIP-Projekt hat sich auf die umfassende Einhaltung der EU-KI-Verordnung verständigt, sich eine weitgehende Übersicht über die entsprechenden rechtlichen Anforderungen verschafft sowie Verantwortlichkeiten im Projekt festgelegt. Viele Anforderungen sind umgesetzt bzw. zur Umsetzung geplant, aber die Vollständigkeit der Abdeckung ist nicht gegeben, die Umsetzungen sind allenfalls teilweise dokumentiert, getestet und evaluiert. | Das KIP-Projekt hat sich auf die umfassende Einhaltung der EU-KI-Verordnung verständigt sowie sich eine umfassende und strukturierte Übersicht über die entsprechenden rechtlichen Anforderungen verschafft, und die Projektbeteiligten darüber informiert. Die Verantwortlichkeiten im Projekt sind festgelegt, und die Verantwortlichen sind in das Projekt eingebunden. Das Vorgehen bei der Umsetzung folgt einem definierten Standard. Alle Anforderungen sind umgesetzt bzw. mit konkreten Deadlines und entsprechenden Ressourcen (Zeit, Personal, sonstige) zur Umsetzung geplant. Die Vollständigkeit der Abdeckung ist sichergestellt, die Umsetzungen sind umfassend dokumentiert, getestet und evaluiert, und die Prozesse und Ergebnisse werden regelmäßig überwacht und aktualisiert. | Das KIP-Projekt hat sich auf die umfassende Einhaltung der EU-KI-Verordnung verständigt sowie sich eine vollständige und systematisch strukturierte Übersicht über die entsprechenden rechtlichen Anforderungen verschafft und allen Projektbeteiligten bekannt gemacht. Die Verantwortlichkeiten im Projekt sind festgelegt, und die Verantwortlichen sind in das Projekt eingebunden. Das Vorgehen bei der Umsetzung folgt einem definierten nationalem oder internationalem Standard, etwa ISO/IEC 42001. Alle Anforderungen sind umgesetzt bzw. mit konkreten Deadlines und entsprechenden Ressourcen (Zeit, Personal, sonstige) zur Umsetzung geplant. Die Vollständigkeit der Abdeckung ist nachweisbar sichergestellt, die Umsetzungen sind vollständig dokumentiert, getestet und evaluiert sowie extern zertifiziert, und die Prozesse und Ergebnisse werden regelmäßig überwacht und aktualisiert. | Trifft nicht zu. | KI F&E Pflege-wissenschaft Praxispartner | PROKIP |

Gefördert durch:
Bundesministerium für Forschung, Technologie und Raumfahrt

| Dimension 2<br>Prozessuale und translationale Voraussetzungen und Aspekte | | | | | | | | | |
|---|---|---|---|---|---|---|---|---|---|
| **Attribut-Nummer** | **Attribute** | **Stufe 1 (initial)** | **Stufe 2 (erkundend)** | **Stufe 3 (entschlossen)** | **Stufe 4 (gesteuert)** | **Stufe 5 (optimiert)** | **Trifft nicht zu** | **Bewertung durch** | **Quelle** |
| **2.1** | Praxispartner: Zeitliche Ressourcen*<br>*Zeitliche Ressourcen können sich auf Führungskräfte und Pflegefachpersonen der Organisation beziehen, aber auch auf weiteres Personal (z.B. IT-Fachpersonal der Organisation, andere Berufsgruppen), das entsprechend einzuplanen ist. | Die zeitlichen Ressourcen für die Planung, Umsetzung und Evaluation von KIP-Projekten [beim Praxispartner] sind unbekannt oder nicht vorhanden oder vorgesehen. Mitarbeitende sind vollständig mit Routineaufgaben ausgelastet.Keine klaren Zeitfenster für die Planung, Umsetzung oder Evaluation von KIP-Projekten vorhanden. | Erste Überlegungen zur Zuweisung von zeitlichen Ressourcen für KIP-Projekte [beim Praxispartner]. Aber keine festen Zeitpläne oder klare Zuordnung vorhanden. Mitarbeitende widmen KIP-Projekten nur gelegentlich Zeit, häufig außerhalb ihrer regulären Arbeitszeiten, was zu Verzögerungen führt. | Spezifische Zeitfenster für die Planung, Umsetzung und Evaluation von KIP-Projekten sind [beim Praxispartner] vorhanden. Diese Ressourcen sind jedoch noch begrenzt und müssen oft mit anderen Aufgaben konkurrieren. Zum Teil sind Mitarbeitende für KIP-Projekte freigestellt, aber die Zeitressourcen sind nicht immer ausreichend | Zeitliche Ressourcen für KIP-Projekte sind [beim Praxispartner] fest eingeplant und werden systematisch verwaltet. Definierte Zeitpläne, die sowohl die Planung als auch die Umsetzung und Evaluation abdecken, sind vorhanden. Mitarbeitende haben regelmäßig dedizierte Zeit für KIP-Projekte, und deren Fortschritt wird überwacht und angepasst. | Die zeitlichen Ressourcen für KIP-Projekte sind vollständig [beim Praxispartner] integriert und optimiert. Mitarbeitende haben ausreichende und flexible Zeitfenster, um alle Phasen der KIP-Projekte effizient zu bewältigen. Zeitressourcen werden kontinuierlich überwacht. Mechanismen zur schnellen Anpassung bei Bedarf, um den Projekterfolg sicherzustellen, sind vorhanden. | Trifft nicht zu. | Praxispartner | 4 |
| **2.2** | Praxispartner: Personelle Ressourcen*: Eigene Personalstelle für die Implementierung von KI in der Organisation<br>*Personelle Ressourcen können sich auf Führungskräfte und Pflegefachpersonen der Organisation beziehen, aber auch auf weiteres Personal (z.B. IT-Fachpersonal der Organisation, andere Berufsgruppen), das entsprechend einzuplanen ist. | Keine dedizierte Stelle oder Position für die Implementierung von KI [beim Praxispartner] vorhanden. Die Verantwortung für KI wird nicht formal zugewiesen, und KIP-Projekte werden gar nicht oder nur sporadisch und ohne klare Führung angegangen. | Erste Überlegungen zur Schaffung einer eigenen Stelle für KI-Implementierung [beim Praxispartner]. Möglicherweise informelle Verantwortliche oder projektbasierte Teams vorhanden, aber keine offiziell definierte Rolle oder Position. Die Aufgaben im Bereich KI werden neben anderen Tätigkeiten wahrgenommen. | Eigene spezifische Stelle für die Implementierung von KI [beim Praxispartner] ist definiert und eingerichtet. Diese Position ist jedoch noch im Aufbau und möglicherweise nicht vollumfänglich besetzt. Die Rolle hat klare Aufgaben, aber die Ressourcen und Befugnisse sind noch begrenzt. | Eigene spezifische Stelle für die Implementierung von KI [beim Praxispartner] ist vollständig etabliert, besetzt und im Unternehmen bekannt. Diese Position ist klar in die Organisationsstruktur eingebunden und verfügt über die notwendigen Ressourcen und Befugnisse, um KI-Initiativen effektiv voranzutreiben. Definierte Prozesse für die Zusammenarbeit mit anderen Abteilungen sind vorhanden. | Eigene spezifische Stelle für die Implementierung von KI [beim Praxispartner] ist vollständig optimiert und strategisch ausgerichtet. Diese Position ist zentral für die Innovationsstrategie der Organisation und hat umfassenden Zugang zu Ressourcen, sowie Einfluss auf Entscheidungsprozesse. Es gibt kontinuierliche Weiterbildung und Anpassung der Rolle, um den sich ändernden Anforderungen gerecht zu werden. | Trifft nicht zu. | Praxispartner | 7 |

| Dimension 2<br>Prozessuale und translationale Voraussetzungen und Aspekte | | | | | | | | | |
|---|---|---|---|---|---|---|---|---|---|
| **Attribut-Nummer** | **Attribute** | **Stufe 1 (initial)** | **Stufe 2 (erkundend)** | **Stufe 3 (entschlossen)** | **Stufe 4 (gesteuert)** | **Stufe 5 (optimiert)** | **Trifft nicht zu** | **Bewertung durch** | **Quelle** |
| **2.3** | Praxispartner: Personelle Ressourcen: Eigene Personalstelle, die sich an Forschung und Entwicklung (F&E) von KI beteiligt | Keine dedizierte Stelle oder Position für die Beteiligung an F&E von KI [beim Praxispartner] vorhanden. Die Beteiligung an F&E von KI wird nicht formal zugewiesen, und KIP-Projekte werden gar nicht oder nur sporadisch und ohne klare Beteiligung angegangen. | Erste Überlegungen zur Schaffung einer eigenen Stelle für die Beteiligung an F&E von KI [beim Praxispartner]. Es gibt möglicherweise informelle Verantwortliche oder projektbasierte Teams, aber keine offiziell definierte Rolle oder Position. Die Aufgaben im Bereich der Beteiligung an F&E von KI werden neben anderen Tätigkeiten wahrgenommen. | Eigene Stelle für Beteiligung an F&E von KI ist [beim Praxispartner] definiert und eingerichtet. Diese Position ist jedoch noch im Aufbau und möglicherweise nicht vollumfänglich besetzt. Die Rolle hat klare Aufgaben, aber die Ressourcen und Befugnisse sind noch begrenzt. | Die Stelle für Beteiligung an F&E von KI [beim Praxispartner] ist vollständig etabliert und besetzt. Diese Position ist klar in die Organisationsstruktur eingebunden und verfügt über die notwendigen Ressourcen und Befugnisse, um F&E effektiv voranzutreiben. Es gibt klare Prozesse für die Zusammenarbeit mit anderen Abteilungen. | Die Stelle für die Beteiligung an F&E von KI [beim Praxispartner] ist vollständig optimiert und strategisch ausgerichtet. Diese Position ist maßgeblich für die Ausrichtung der F&E an KI in der Organisation verantwortlich und hat umfassenden Zugang zu Ressourcen, sowie Einfluss auf Entscheidungsprozesse. Es gibt kontinuierliche Weiterbildung und Anpassung der Rolle, um den sich ändernden Anforderungen gerecht zu werden. | Trifft nicht zu. | Praxispartner | 7 |
| **2.4** | Praxispartner: Personelle Ressourcen: Verfügbare personelle Ressourcen für KIP-Projekte und KI-Integration in der Organisation | Keine dedizierten Stellen oder Positionen für KIP-Projekte und KI-Integration, z.B. in der IT-Abteilung oder im Management [beim Praxispartner] vorhanden. Die Verantwortung für KIP-Projekte wird nicht formal zugewiesen und KIP-Projekte werden sporadisch und ohne klare Führung angegangen. | Erste Überlegungen zur Schaffung eigener Stellen für KIP-Projekte und KI-Integration [beim Praxispartner]. Es gibt möglicherweise informelle Verantwortliche oder projektbasierte Teams, aber keine offiziell definierte Rolle oder Position. Die Aufgaben in KIP-Projekten werden neben anderen Tätigkeiten wahrgenommen. | Eigene Stellen für KIP-Projekte und KI-Integration sind [beim Praxispartner] definiert und eingerichtet. Diese Positionen sind jedoch noch im Aufbau und möglicherweise nicht vollumfänglich besetzt. Die Rolle hat klare Aufgaben, aber die Ressourcen und Befugnisse sind noch begrenzt. | Die Stellen für KIP-Projekte und KI-Integration sind [beim Praxispartner] vollständig etabliert und besetzt. Diese Position ist klar in die Organisationsstruktur eingebunden und verfügt über die notwendigen Ressourcen und Befugnisse, um KI-Initiativen effektiv voranzutreiben. Es gibt klare Prozesse für die Zusammenarbeit mit anderen Abteilungen. | Die Stellen für KIP-Projekte und KI-Integration [beim Praxispartner] sind vollständig optimiert und strategisch ausgerichtet. Die Stellen haben umfassenden Zugang zu Ressourcen. Es gibt kontinuierliche Weiterbildung und Anpassung der Rolle, um den sich ändernden Anforderungen gerecht zu werden | Trifft nicht zu. | Praxispartner | 4 |

| Dimension 2<br>Prozessuale und translationale Voraussetzungen und Aspekte | | | | | | | | | |
|---|---|---|---|---|---|---|---|---|---|
| **Attribut-Nummer** | **Attribute** | **Stufe 1 (initial)** | **Stufe 2 (erkundend)** | **Stufe 3 (entschlossen)** | **Stufe 4 (gesteuert)** | **Stufe 5 (optimiert)** | **Trifft nicht zu** | **Bewertung durch** | **Quelle** |
| **2.5** | Praxispartner: Personelle Ressourcen: Verfügbare Data Scientists*<br>*Data Scientists nutzen Datenanalyse, maschinelles Lernen und Statistik auf der Datengrundlage des Praxispartners, um die pflegerische Versorgung zu verbessern, betriebliche Abläufe zu optimieren und Forschung zu unterstützen. Praxispartnern, die eigene Data Scientists beschäftigen fällt es leichter, in der Organisation vorhandene unstrukturierte und strukturierte Daten für KIP-Projekte nutzbar zu machen. | Keine Data Scientists [beim Praxispartner] im KIP-Projekt vorhanden. Externe Beratung wird gar nicht oder nur bei Bedarf und sehr sporadisch genutzt. | Erste Überlegungen zur Einstellung oder zum Einkauf von Data Scientists [beim Praxispartner], aber noch keine fest angestellte oder eingekaufte Expertise im Projektverbund/ Team. | Ein oder mehrere Data Scientists wurden [beim Praxispartner] eingestellt oder eingekauft, aber ihre Integration und Einfluss sind noch begrenzt. | Ausreichend viele Data Scientists sind fest im Team [des Praxispartners] eingebunden und aktiv an KIP-Projekten beteiligt. | Data Scientists sind im Team [des Praxispartners] vollständig integriert, strategisch eingebunden und spielen eine zentrale Rolle im KIP-Projekt. | Trifft nicht zu. | Praxispartner mit IT-Fach-personal<br>KI F&E | 5 |
| **2.6** | **Praxispartner: Personelle Ressourcen: Verfügbare Data Champions***<br>*Data Champions kennen die beim Praxispartner anfallenden Arten von Daten, setzen sich besonders für den Umgang mit Daten ein und vermitteln zwischen dem Pflegefachpersonal und der IT-Abteilung. Sie stellen sicher, dass verschiedene Arten von Daten korrekt, vollständig und aktuell sind. Sie identifizieren und beheben Probleme in der Datenerfassung. Sie fördern die Datenkompetenz der Mitarbeitenden durch Schulungen und sensibilisieren in der Organisation für die Bedeutung von Daten in der Versorgung und Forschung. | Keine Data Champions [beim Praxispartner] im KIPProjekt vorhanden, die Nutzung von Daten wird nicht aktiv gefördert oder unterstützt. | Erste Data Champions [beim Praxispartner] werden identifiziert, aber ihre Rolle ist noch nicht formalisiert oder anerkannt. | Einige Data Champions sind [beim Praxispartner] aktiv, ihre Rolle ist definiert, und sie fördern die Datenkultur in ausgewählten Bereichen. | Data Champions sind [beim Praxispartner] etabliert und arbeiten bereichsübergreifend, um die Datennutzung zu fördern und zu unterstützen. | Data Champions sind vollständig in die Unternehmensstruktur [des Praxispartners] integriert und treiben aktiv eine datengetriebene Kultur voran | Trifft nicht zu. | Praxispartner mit IT-Fach-personal<br>KI F&E | 5 |

| Dimension 2<br>Prozessuale und translationale Voraussetzungen und Aspekte | | | | | | | | | |
|---|---|---|---|---|---|---|---|---|---|
| **Attribut-Nummer** | **Attribute** | **Stufe 1 (initial)** | **Stufe 2 (erkundend)** | **Stufe 3 (entschlossen)** | **Stufe 4 (gesteuert)** | **Stufe 5 (optimiert)** | **Trifft nicht zu** | **Bewertung durch** | **Quelle** |
| **2.7** | Praxispartner: Digitalisierungsgrad generell*<br>*Genereller Digitalisierungsgrad in dem Unternehmen des Praxispartners und in den unterschiedlichen Organisationsbereichen und Datengrundlagen (z.B. Personaldaten, Pflege-/Patient:innendaten, Verwaltungsdaten etc.) | Der generelle Digitalisierungsgrad [des Praxispartners] ist unbekannt oder es gibt nur nicht digitalisierte Daten (z.B. papierbasierte Daten). | Geringer Anteil digitalisierter Daten in Pflege-/Patient:innen und Personaldaten (z.B. nur zentrale organisatorische Abläufe) [des Praxispartners]. | Mittlerer Anteil digitalisierter Daten in Pflege-/Patient:innen und Personaldaten (z.B. nur zentrale Qualitätsindikatoren) [beim Praxispartner] . | Hoher Anteil digitalisierter Daten in Pflege-/Patient:innen und Personaldaten (z.B. Zeitreihendaten über Krankheitsverläufe) [beim Praxispartner] . | Vollständig digitalisierte Daten in Pflege-/Patient:innen und Personaldaten [beim Praxispartner] . | Trifft nicht zu. | Praxispartner mit IT-Fach-personal<br>KI F&E | 1,4,5 |
| **2.8** | Praxispartner: Digitalisierungsgrad KI-spezifisch | Der KI-spezifische Digitalisierungsgrad [des Praxispartners] ist unbekannt oder ist sehr gering oder nicht vorhanden. KI-Systeme werden nicht genutzt. | Erste Ansätze zur Digitalisierung mit Fokus auf KI werden [beim Praxispartner] erkundet, aber noch nicht umfassend umgesetzt. | Der Praxispartner hat erste KI-Systeme integriert, der Digitalisierungsgrad mit Fokus auf KI ist jedoch noch auf einzelne Anwendungsfälle oder Bereiche begrenzt. | Der Praxispartner ist weitgehend digitalisiert, mit einem starken Fokus auf die Implementierung und Nutzung von KI-Systemen. | Der Praxispartner ist vollständig digitalisiert, und KI-Systeme sind tief in alle Prozesse integriert, mit kontinuierlicher Optimierung des Digitalisierungsgrades. | Trifft nicht zu. | Praxispartner mit IT-Fach-personal<br>KI F&E | 4,5 |
| **2.9** | Praxispartner: Digitalisierungsgrad: Qualitätsstandards für Daten | Der Qualitätsstandard für Daten [beim Praxispartner] ist unbekannt oder kein einheitlicher Qualitätsstandard für Daten vorhanden. | Erste Forderungen nach Vereinheitlichung der Datenqualität [beim Praxispartner]. | Interne Qualitätsstandards (z. B. für Schlüsselterminologien wie die International Classification of Diseases ICD) [beim Praxispartner]. | Pflegeeinrichtungs-/ Klinik-übergreifende Qualitätsstandards (z. B. für Schlüsselterminologien) [beim Praxispartner]. | Internationale Qualitätsstandards [beim Praxispartner]. | Trifft nicht zu. | Praxispartner mit IT-Fach-personal<br>KI F&E | 1 |
| 2.10 | Praxispartner: Digitalisierungsgrad: Standard Datensammlung*<br>*Gemeint ist ein Standard für die Sammlung von Daten, die bei dem Praxispartner als Routinedaten im alltäglichen Pflege- und Organisationsprozess anfallen. | Prozesse zur Datensammlung [beim Praxispartner] sind unbekannt oder keine standardisierten Prozesse zur Datensammlung vorhanden. Daten werden sporadisch und unsystematisch erhoben. | Erste Überlegungen zur Standardisierung der Datensammlung [beim Praxispartner] werden angestellt, jedoch noch nicht umgesetzt. | Es existieren teilweise standardisierte Prozesse zur Datensammlung [beim Praxispartner], die jedoch noch lückenhaft sind. | Die Datensammlung [beim Praxispartner] ist weitgehend standardisiert und systematisch, jedoch nicht durchgängig optimiert. | Die Datensammlung [beim Praxispartner] ist vollständig standardisiert, systematisch und optimiert, mit regelmäßiger Überprüfung und Anpassung. | Trifft nicht zu. | Praxispartner mit IT-Fach-personal<br>KI F&E | 3 |
| **2.11** | Praxispartner: Digitalisierungsgrad: Standard Datenspeicherung | Verfahren zur Datenspeicherung [beim Praxispartner] sind unbekannt oder keine standardisierten vorhanden. Daten werden unsystematisch und dezentral gespeichert. | Erste Schritte zur Standardisierung der Datenspeicherung [beim Praxispartner] werden unternommen, aber nicht konsequent umgesetzt. | Teilweise standardisierte Verfahren zur Datenspeicherung [beim Praxispartner], jedoch noch lückenhaft oder mit Verbesserungspotenzial. | Die Datenspeicherung ist [beim Praxispartner] weitgehend standardisiert und zentralisiert, mit klaren Prozessen. | Die Datenspeicherung [beim Praxispartner] ist vollständig standardisiert, zentralisiert und optimiert, mit kontinuierlicher Verbesserung. | Trifft nicht zu. | Praxispartner mit IT-Fach-personal<br>KI F&E | 3 |

| Dimension 2<br>Prozessuale und translationale Voraussetzungen und Aspekte | | | | | | | | | |
|---|---|---|---|---|---|---|---|---|---|
| **Attribut-Nummer** | **Attribute** | **Stufe 1 (initial)** | **Stufe 2 (erkundend)** | **Stufe 3 (entschlossen)** | **Stufe 4 (gesteuert)** | **Stufe 5 (optimiert)** | **Trifft nicht zu** | **Bewertung durch** | **Quelle** |
| **2.12** | **Praxispartner: Digitalisierungsgrad: Standard Datenaustausch** | Standards für den Datenaustausch [beim Praxispartner] sind unbekannt oder der Datenaustausch erfolgt sporadisch und unsystematisch, ohne festgelegte Standards. | Erste Ansätze zur Standardisierung des Datenaustauschs [beim Praxispartner] sind vorhanden, aber noch nicht vollständig implementiert. | Es existieren teilweise standardisierte Verfahren für den Datenaustausch [beim Praxispartner], die jedoch noch lückenhaft sind oder optimiert werden müssen. | Der Datenaustausch [beim Praxispartner] ist weitgehend standardisiert und effizient, jedoch nicht durchgängig optimiert. | Der Datenaustausch [beim Praxispartner] ist vollständig standardisiert, effizient und optimiert, mit klaren, etablierten Prozessen. | Trifft nicht zu. | Praxispartner mit IT-Fach-personal<br>KI F&E | 3 |
| **2.13** | Praxispartner: Digitalisierungsgrad: Standard ontologische Repräsentation*<br>*Ontologische Repräsentation meint die strukturierte Darstellung von Wissen in einem bestimmten Fachgebiet (z. B. Medizin), um Daten einheitlich, interpretierbar und qualitativ hochwertig zu machen. Ein Standard für eine ontologische Repräsentation hilft dabei, Daten semantisch zu strukturieren und zu standardisieren, sodass sie verständlich, vergleichbar und interoperabel werden. Ein Krankenhaus kann z.B. verschiedene Begriffe für dieselbe Erkrankung an verschiedenen Dokumentationsorten verwenden: "Herzinfarkt", "Myokardinfarkt", "ICD-10: I21". Eine ontologische Repräsentation würde diese Begriffe einer einheitlichen, standardisierten Definition zuordnen. Dadurch könnten IT-Systeme, Gesundheitsfachpersonal oder auch KI-Entwickler:innen eindeutig erkennen, dass sie sich auf dieselbe Erkrankung beziehen. | Keine Standards für die ontologische Repräsentation von Daten [beim Praxispartner] im KIP-Projekt vorhanden oder Standards sind unbekannt. | Erste Überlegungen zur Einführung ontologischer Standards [beim Praxispartner] im KI-Pflege-Projekt, aber noch keine konkrete Umsetzung. | Teilweise implementierte ontologische Standards [beim Praxispartner] im KI-Pflege-Projekt, die jedoch noch Lücken und Inkonsistenzen aufweisen. | Ontologische Standards sind [beim Praxispartner] im KIP-Projekt weitgehend implementiert und konsistent, aber nicht vollständig optimiert. | Die ontologische Repräsentation von Daten [beim Praxispartner] im KIP-Projekt ist vollständig standardisiert, konsistent und optimiert. | Trifft nicht zu. | Praxispartner mit IT-Fach-personal<br>KI F&E | 3 |

| Dimension 2<br>Prozessuale und translationale Voraussetzungen und Aspekte | | | | | | | | | |
|---|---|---|---|---|---|---|---|---|---|
| **Attribut-Nummer** | **Attribute** | **Stufe 1 (initial)** | **Stufe 2 (erkundend)** | **Stufe 3 (entschlossen)** | **Stufe 4 (gesteuert)** | **Stufe 5 (optimiert)** | **Trifft nicht zu** | **Bewertung durch** | **Quelle** |
| **2.14** | Praxispartner: Bereitschaft zur digitalen Transformation: Haltung und Umgang mit KI in der Organisation | Die Bereitschaft zur digitalen Transformation bei dem Praxispartner im KIP-Projekt ist unbekannt oder mangelnde oder gar keine Bereitschaft zur digitalen Transformation erkennbar, KI-Systeme werden kritisch betrachtet oder ignoriert. | Erste Schritte zur digitalen Transformation [beim Praxispartner] im KI-Pflege-Projekt, KI-Systeme werden zögerlich akzeptiert, aber es gibt noch viele Vorbehalte. | Die digitale Transformation wird aktiv [beim Praxispartner] im KIP-Projekt vorangetrieben, und KI-Systeme sind zunehmend akzeptiert, aber es gibt noch Widerstände. | Hohe Bereitschaft zur digitalen Transformation [beim Praxispartner] im KI-Pflege-Projekt, KI-Systeme sind weitgehend akzeptiert und in die Prozesse integriert. | Volle Bereitschaft zur digitalen Transformation [beim Praxispartner] im KI-Pflege-Projekt, KI-Systeme werden als wesentlicher Bestandteil der Unternehmensstrategie verstanden und aktiv gefördert. | Trifft nicht zu. | Praxispartner | 3,5 |
| **2.15** | Praxispartner: Bereitschaft zur digitalen Transformation: Unterstützung von KI durch Führungskräfte und Stakeholder:innen*<br>*Stakeholder:innen sind alle Einzelpersonen oder Gruppen, die das Arbeitsklima in einer Organisation beeinflussen – wie Führungskräfte, Teamleitungen, Mitarbeitende, Personalverantwortliche und mitunter auch externe Berater:innen – deren Handlungen, Entscheidungen und Interaktionen die Organisationskultur, Kommunikation, das Vertrauen, die Motivation und das gesamte Arbeitsumfeld prägen. | Führungskräfte und Stakeholder:innen [beim Praxispartner] unterstützen KI-Systeme nicht oder nur sehr eingeschränkt. | Erste Anzeichen von Unterstützung durch Führungskräfte und Stakeholder:innen [beim Praxispartner], aber noch zögerlich. | Führungskräfte und Stakeholder:innen [beim Praxispartner] unterstützen KI-Systeme aktiv, aber noch nicht durchgängig. | Hohe Unterstützung durch Führungskräfte und Stakeholder:innen [beim Praxispartner], die sich aktiv für KIP-Projekte engagieren. | Führungskräfte und Stakeholder:innen [beim Praxispartner] unterstützen KI-Systeme vollständig und sind zentrale Treiber der KI-Strategie im Unternehmen. | Trifft nicht zu. | Praxispartner | 5 |
| **2.16** | **Praxispartner: Bereitschaft zur digitalen Transformation: Eigene KI-Strategie der Organisation** | Keine KI-Strategie im Unternehmen des Praxispartner im KIP-Projekt vorhanden. | Erste Überlegungen zur Entwicklung einer KI-Strategie [beim Praxispartner], aber noch keine konkrete Ausrichtung. | Eine KI-Strategie ist [beim Praxispartner] definiert, aber noch in der frühen Implementierungsphase und nicht vollständig transparent, kommuniziert und integriert. | Eine KI-Strategie ist [beim Praxispartner] implementiert, transparent, kommuniziert und in die Geschäftsstrategie integriert, wird aber noch optimiert. | Eine vollständig integrierte und optimierte KI-Strategie [beim Praxispartner], die kontinuierlich überprüft und aktualisiert wird. | Trifft nicht zu. | Praxispartne r | 1,3 |

Gefördert durch:
Bundesministerium für Forschung, Technologie und Raumfahrt

| Dimension 2<br>Prozessuale und translationale Voraussetzungen und Aspekte | | | | | | | | | |
|---|---|---|---|---|---|---|---|---|---|
| **Attribut-Nummer** | **Attribute** | **Stufe 1 (initial)** | **Stufe 2 (erkundend)** | **Stufe 3 (entschlossen)** | **Stufe 4 (gesteuert)** | **Stufe 5 (optimiert)** | **Trifft nicht zu** | **Bewertung durch** | **Quelle** |
| **2.17** | Praxispartner: Bereitschaft zur digitalen Transformation: Eigene Data-Governance-Strategie der Organisation*<br>*Data Governance formalisiert Entscheidungsrechte, Verfahren und Kontrollen, um die bei der Verarbeitung sowie gemeinsamen Nutzung von Daten zwischen den beteiligten Akteuren auftretenden Interessenskonflikte so gut es geht aufzulösen. Dafür ist es erforderlich, sowohl den Wert der Daten als auch die mit ihrer Verarbeitung verbundenen Risiken je nach Perspektive der beteiligten Akteure möglichst optimal zu schöpfen bzw. zu kontrollieren. | Keine Data-Governance-Strategie [beim Praxispartner] vorhanden. | Erste Überlegungen zur Entwicklung einer Data-Governance-Strategie [beim Praxispartner], aber noch keine konkrete Umsetzung. | Eine Data-Governance-Strategie ist [beim Praxispartner] definiert, aber noch in der frühen Implementierungsphase und nicht vollständig transparent, kommuniziert und integriert. | Die Data-Governance-Strategie ist [beim Praxispartner] implementiert, tranparent, kommuniziert und in die Geschäftsstrategie integriert, wird aber noch optimiert. | Eine vollständig integrierte und optimierte Data-Governance-Strategie [beim Praxispartner], die kontinuierlich überprüft und aktualisiert wird. | Trifft nicht zu. | Praxispartner | 1,3-7 |
| **2.18** | **Praxispartner: Bereitschaft zur digitalen Transformation: eigene IT-Governance-Strategie der Organisation***<br>*IT Governance ist der strategische Rahmen, der sicherstellt, dass die IT eines Unternehmens oder einer Organisation effektiv, sicher und regelkonform betrieben wird. Sie sorgt dafür, dass IT-Ressourcen optimal eingesetzt werden, um die Unternehmensziele zu unterstützen. | Keine IT-Governance-Strategie [beim Praxispartner] vorhanden. | Erste Überlegungen zur Entwicklung einer IT-Governance-Strategie [beim Praxispartner], aber noch keine klare Ausrichtung. | Eine IT-Governance-Strategie ist [beim Praxispartner] definiert, aber noch in der frühen Implementierungsphase und nicht vollständig transparent, kommuniziert und integriert. | Die IT-Governance-Strategie ist [beim Praxispartner] implementiert, transparent, kommuniziert und in die Geschäftsstrategie integriert, wird aber noch optimiert. | Eine vollständig integrierte und optimierte IT-Governance-Strategie [beim Praxispartner], die kontinuierlich überprüft und aktualisiert wird. | Trifft nicht zu. | Praxispartner | 3 |

| Dimension 2<br>Prozessuale und translationale Voraussetzungen und Aspekte | | | | | | | | | |
|---|---|---|---|---|---|---|---|---|---|
| **Attribut-Nummer** | **Attribute** | **Stufe 1 (initial)** | **Stufe 2 (erkundend)** | **Stufe 3 (entschlossen)** | **Stufe 4 (gesteuert)** | **Stufe 5 (optimiert)** | **Trifft nicht zu** | **Bewertung durch** | **Quelle** |
| **2.19** | Praxispartner: Technikakzeptanz: Akzeptanz von KI durch Führungskräfte, Pflegefachpersonen und andere Stakeholder:innen* *Stakeholder:innen sind alle weiteren relvanten Personengruppen, die nicht die Pflegeempfänger:innen oder Patient:innen sind. Je nach KIP-Projekt können das z.B. auch gesetzliche Betreuer:innen, Hausärzt:innen oder Konsildienste sein. Eine Analyse der relevanten Stakeholder:innen sollte Teil jeder Planung eines KIP-Projektes sein. | Die Akzeptanz von KI-Systemen durch Führungskräfte, Pflegefachpersonen und andere Stakeholder:innen [beim Praxispartner] ist unbekannt oder geringe bis keine Akzeptanz von KI. Pflegefachpersonen lehnen die Nutzung von KI-Technologien am Arbeitsplatz weitgehend ab oder haben starke Vorbehalte. | Erste Anzeichen von Akzeptanz durch Führungskräfte oder Pflegefachpersonen oder andere Stakeholder:innen [beim Praxispartner], aber noch weit verbreitete Skepsis und Zurückhaltung bei allen Gruppen. | Akzeptanz von KI wächst, Führungskräfte und Pflegefachpersonen oder andere Stakeholder:innen [beim Praxispartner] zeigen zunehmendes Interesse an der Nutzung von KI-Systemen, aber es gibt noch Unsicherheiten und Widerstände. | Hohe Akzeptanz der Nutzung von KI-Technologien, Führungskräfte, Pflegefachpersonen und andere Stakeholder:innen [beim Praxispartner] unterstützen aktiv die Implementierung von KI-Systemen und die Umsetzung von KIP-Projekten. | Vollständige Akzeptanz der Nutzung von KI-Systemen,Führungskräfte, Pflegefachpersonen und andere Stakeholder:innen [beim Praxispartner] treiben die KI-Integration proaktiv voran. Pflegefachpersonen akzeptieren die Nutzung von KI-Systemen am Arbeitsplatz vollständig und sehen sie als wertvolles Werkzeug zur Unterstützung ihrer Arbeit. | Trifft nicht zu. | Praxispartner Pflege-wissenschaft | 3 |
| **2.20** | Praxispartner: Technikakzeptanz: Akzeptanz von KI durch Pflegeempfänger:innen, Patient:innen und ihre An- und Zugehörigen | Die Akzeptanz von KI-Systemen durch Pflegeempfänger:innen, Patient:innen und ihre An- und Zugehörigen ist unbekannt oder Pflegebedürftige/Patient:innen lehnen KI-Technologien in der Versorgung weitgehend ab oder haben starke Vorbehalte. | Erste Anzeichen von Akzeptanz von KI-Systemen in der Versorgung durch Pflegeempfänger:innen, Patient:innen und ihre An- und Zugehörigen, aber noch weit verbreitete Skepsis. | Akzeptanz von KI wächst, Pflegeempfänger:innen, Patient:innen und ihre An- und Zugehörigen akzeptieren KI-Systeme in ihrer Versorgung zunehmend, aber es gibt noch Unsicherheiten und Widerstände. | Hohe Akzeptanz von KI-Systemen durch Pflegeempfänger:innen, Patient:innen und ihre An- und Zugehörigen, die deren Vorteile in ihrer Versorgung zunehmend erkennen. | Pflegeempfänger:innen, Patient:innen und ihre An- und Zugehörigen akzeptieren KI-Systeme vollständig und sehen sie als wertvollen Bestandteil ihrer Versorgung. | Trifft nicht zu. | Praxispartner Pflege-wissenschaft | 1,5,6 |

| Dimension 2<br>Prozessuale und translationale Voraussetzungen und Aspekte | | | | | | | | | |
|---|---|---|---|---|---|---|---|---|---|
| **Attribut-Nummer** | **Attribute** | **Stufe 1 (initial)** | **Stufe 2 (erkundend)** | **Stufe 3 (entschlossen)** | **Stufe 4 (gesteuert)** | **Stufe 5 (optimiert)** | **Trifft nicht zu** | **Bewertung durch** | **Quelle** |
| **2.21** | Praxispartner: Technikakzeptanz: Akzeptanz von KI durch Personalvertretungen und deren Einbezug | Die Akzeptanz von KI-Systemen durch Personalvertretungen (z.B. Mitarbeitervertretung, Personalrat) [beim Praxispartner] ist unbekannt oder geringe bis keine Akzeptanz von KI. Personalvertretungen sind (noch) nicht in die Umsetzung des KIP-Projektes eingebunden oder lehnen die Nutzung von KI-Technologien am Arbeitsplatz weitgehend ab oder haben starke Vorbehalte. | Erste Anzeichen von Akzeptanz durch Personalvertretungen [beim Praxispartner], aber noch weit verbreitete Skepsis und Zurückhaltung. Erste Bemühungen zum Einbezug von Personalvertretungen in die Umsetzung des Kip-Projektes. Diese sind aber noch unsystematisch und werden nicht dokumentiert. | Akzeptanz von KI wächst, Personalvertretungen zeigen zunehmendes Interesse an der Nutzung von KI-Systemen, aber es gibt noch Unsicherheiten und Widerstände. Der Einbezug von Personalvertretungen in die Umsetzung des KIP-Projektes ist systematisch eingeplant, erfolgt aber noch lückenhaft oder hohe Klärungsbedarfe oder andauernde Abstimmungsprozesse mit der Personalvertretung verhindern (noch) das Fortschreiten des KIP-Projekts. | Hohe Akzeptanz der Nutzung von KI-Technologien, Personalvertretungen [beim Praxispartner] unterstützen aktiv die Implementierung von KI-Systemen und die Umsetzung von KIP-Projekten. | Vollständige Akzeptanz der Nutzung von KI-Systemen, Personalvertretungen [beim Praxispartner] treiben die KI-Integration proaktiv voran und sehen sie als wertvolles Werkzeug zur Unterstützung der Mitarbeitenden [des Praxispartners]. | Trifft nicht zu. | Praxispartner Pflege-wissenschaft | 1,6 |
| **2.22** | Praxispartner: Erwartungen und Vorbehalte | Erwartungen und Vorbehalte [seitens des Praxispartners] gegenüber KI-Systemen sind unbekannt oder werden nicht systematisch erfasst oder berücksichtigt. | Erste Ansätze zur Erfassung von Erwartungen und Vorbehalten gegenüber KI-Systemen beim Praxispartner, aber noch kaum systematisch und in den Projektablauf integriert. | Erwartungen und Vorbehalte gegenüber KI-Systemen werden systematisch erfasst, aber es gibt noch Lücken in der systematischen Erfassung und Berücksichtigung der Erwartungen und Vorbehalte. | Erwartungen und Vorbehalte gegenüber KI-Systemen beim Praxispartner sind systematisch und umfassend erfasst und werden aktiv in die Projektplanung und -umsetzung einbezogen. | Erwartungen und Vorbehalte gegenüber KI-Systemen beim Praxispartner werden kontinuierlich erfasst, proaktiv adressiert und in die strategische Planung des KIP-Projektes integriert. | Trifft nicht zu. | Praxispartner Pflege-wissenschaft KI F&E | PROKIP |
| **2.23** | Praxispartner: Wissen und Kompetenzen: Aus-, Fort- und Weiterbildung zu KI und Digitaler Kompetenz in der Organisation | Keine Aus-, Fort- und Weiterbildungsangebote zu KI-Systemen und zu digitalen Kompetenz [beim Praxispartner] vorhanden. | Erste Schulungen und Weiterbildungen zu KI-Systemen und digitaler Kompetenz werden [beim Praxispartner] angeboten, jedoch nur vereinzelt und ohne klare Struktur. | Regelmäßige Schulungen und Weiterbildungen zu KI-Systemen und digitaler Kompetenz werden [beim Praxispartner] angeboten, aber noch nicht umfassend von den geeigneten Mitarbeitenden genutzt. | Umfassende Aus-, Fort- und Weiterbildungsangebote zu KI-Systemen und Digitaler Kompetenz sind [beim Praxispartner] etabliert und werden regelmäßig genutzt. | Vollständig integrierte Aus-, Fort- und Weiterbildungsstrategien zu KI-Systemen und Digitaler Kompetenz [beim Praxispartner], die kontinuierlich angepasst und optimiert werden. | Trifft nicht zu. | Praxispartner | 1,5 |
| **2.24** | Praxispartner: Wissen und Kompetenzen: Verfügbares KI-Wissen des Personals | Das Wissen des Personals im KIP-Projekt [und bei dem Praxispartner] ist unbekannt oder das Personal hat wenig bis kein Wissen über KI. | Erste Schritte zur Wissensvermittlung werden im KIP-Projekt und beim Praxispartner unternommen, aber das verfügbare KI-Wissen ist noch begrenzt. | Das Personal verfügt über grundlegendes KI-Wissen, aber es gibt noch erhebliche Wissenslücken. | Das Personal hat ein umfassendes Verständnis von KI-Systemen, das in den meisten Bereichen angewendet wird. | Das Personal verfügt über tiefgehendes und aktuelles KI-Wissen, das kontinuierlich erweitert und auf dem neuesten Stand gehalten wird. | Trifft nicht zu. | Praxispartner | 3,8 |

| Dimension 2<br>Prozessuale und translationale Voraussetzungen und Aspekte | | | | | | | | | |
|---|---|---|---|---|---|---|---|---|---|
| **Attribut-Nummer** | **Attribute** | **Stufe 1 (initial)** | **Stufe 2 (erkundend)** | **Stufe 3 (entschlossen)** | **Stufe 4 (gesteuert)** | **Stufe 5 (optimiert)** | **Trifft nicht zu** | **Bewertung durch** | **Quelle** |
| **2.25** | Praxispartner: Wissen und Kompetenzen: Echtzeit- und/oder prädiktive Analysen | Keine Fähigkeiten zur Durchführung von Echtzeit- oder prädiktiven Analysen beim Praxispartner oder Fähigkeiten sind unbekannt. | Erste Ansätze zur Durchführung von Echtzeit- oder prädiktiven Analysen beim Praxispartner werden entwickelt, aber noch nicht systematisch angewendet. | Echtzeit- und prädiktive Analysen werden beim Praxispartner teilweise durchgeführt, aber noch nicht optimiert oder umfassend genutzt. | Der Praxispartner führt regelmäßig Echtzeit- und prädiktive Analysen durch, die gut in die Prozesse integriert sind. | Echtzeit- und prädiktive Analysen sind [beim Praxispartner] vollständig integriert und optimiert, und sie sind ein wesentlicher Bestandteil der Entscheidungsfindung. | Trifft nicht zu. | Praxispartner | 1,4,6,8 |
| **2.26** | Praxispartner: Wissen und Kompetenzen: Verfügbare Verfahren für den Wissenstransfer in der Organisation | Keine etablierten Verfahren für den Wissenstransfer [beim Praxispartner] oder verfügbare Verfahren sind unbekannt. | Erste Ansätze für den Wissenstransfer sind vorhanden, aber noch nicht systematisch umgesetzt. | Wissenstransfer wird teilweise organisiert, aber es gibt noch Lücken in der Konsistenz und Verbreitung. | Verfahren für den Wissenstransfer sind etabliert und werden regelmäßig angewendet, aber es gibt noch Potenzial zur Optimierung. | Der Wissenstransfer ist vollständig systematisiert, optimiert und integraler Bestandteil der Organisationskultur. | Trifft nicht zu. | Praxispartner | 5 |
| **2.27** | Praxispartner: Immaterielle Güter: Verfügbarkeit und Ausmaß immaterieller Güter im Kontext KI in der Organisation (Bücher, Fachbeiträge, Preise, strategische Partnerschaften, Konsultationen ...) *<br>*Auch immaterielle Güter in Bezug auf Digitalisierung und zu Digitalen Kompetenzen können für Praxispartner in KIP-Projekten wertvoll sein. | Keine oder sehr wenige immateriellen Güter zu KI [beim Praxispartner] oder immaterielle Güter sind unbekannt. | Erste immaterielle Güter werden [beim Praxispartner] identifiziert und gelegentlich genutzt, aber noch nicht umfassend. | Ein wachsendes Repertoire an immateriellen Gütern ist vorhanden und wird aktiv [beim Praxispartner] genutzt, jedoch noch nicht vollständig integriert (steht etwa nicht allen geeigneten Personen frei zur Verfügung). | Umfassende Sammlung und systematische Nutzung immaterieller Güter, die regelmäßig erweitert werden und geeigneten Personen frei zur Verfügung stehen. | Umfassende Sammlung und systematische Nutzung immaterieller Güter, die regelmäßig erweitert werden und geeigneten Personen frei zur Verfügung stehen. Immaterielle Güter werden regelmäßig überprüft und erweitert. | Trifft nicht zu. | Praxispartner | 6 |
| **2.28** | Praxispartner: Finanzielle Ressourcen und Investitionen: Verfügbare finanzielle Ressourcen für KIP-Projekte und KI-Integration*<br>*Finanzielle Ressourcen für KIP-Projekte können auch in allgemeinen Digitalisierungsbudgets oder anderen Budgets (Innovationsbudget, Teilhabebudget) enthalten sein. | Keine spezifischen finanziellen Ressourcen für KIP-Projekte [beim Praxispartner] verfügbar. | Erste finanzielle Ressourcen für KIP-Projekte werden [beim Praxispartner] bereitgestellt, aber in begrenztem Umfang. | Ausreichende finanzielle Ressourcen werden [beim Praxispartner] für die meisten KIP-Projekte zugewiesen, aber es fehlt an langfristiger Planung. | Umfassende und nachhaltige finanzielle Ressourcen für für KIP-Projekte sind vorhanden und werden regelmäßig aufgestockt und angepasst. | Vollständig optimierte und kontinuierlich angepasste finanzielle Ressourcen für für KIP-Projekte beim Praxispartner, die strategisch für KIP-Projekte und -Integration eingesetzt werden. | Trifft nicht zu. | Praxispartner | 5 |

| Dimension 2<br>Prozessuale und translationale Voraussetzungen und Aspekte | | | | | | | | | |
|---|---|---|---|---|---|---|---|---|---|
| **Attribut-Nummer** | **Attribute** | **Stufe 1 (initial)** | **Stufe 2 (erkundend)** | **Stufe 3 (entschlossen)** | **Stufe 4 (gesteuert)** | **Stufe 5 (optimiert)** | **Trifft nicht zu** | **Bewertung durch** | **Quelle** |
| **2.29** | Finanzielle Ressourcen und Investitionen: Prüfung alternativer Finanzierungsformate für die Einbindung von Praxispartnern | Alternative Finanzierungsformate für die Einbindung von Praxispartnern sind unbekannt oder es wird kaum bis gar nicht über alternative Finanzierungsformate nachgedacht. | Erste alternative Finanzierungsformate werden geprüft oder entwickelt, aber noch nicht genutzt. | Alternativen Finanzierungsformate werden geprüft und teilweise genutzt. | Es gibt etablierte alternative Finanzierungsformate, die regelmäßig genutzt und überprüft werden. | Alternative Finanzierungsformate werden genutzt, mit regelmäßiger erneuter Überprüfung und Anpassung der Suche nach alternativen Finanzierungsformaten an neue Anforderungen. | Trifft nicht zu. | KI F&E<br>Pflege-wissenschaft<br>Praxispartner | 1,3-5 |
| **2.30** | Finanzielle Ressourcen und Investitionen: Management: Nachhaltige Passung von Ressourcen und Investitionen | Keine erkennbare Verbindung zwischen Ressourcen, Investitionen und Projektzielen. | Erste Schritte zur Abstimmung von Ressourcen und Investitionen auf Projektziele werden unternommen. | Ressourcen und Investitionen sind weitgehend auf die Projektziele abgestimmt, aber es gibt noch Lücken. | Ressourcen und Investitionen sind systematisch auf die Projektziele abgestimmt und nachhaltig gesichert. | Ressourcen und Investitionen sind vollständig integriert und optimal auf die langfristigen Ziele und den Nutzen des Projekts abgestimmt. | Trifft nicht zu. | KI F&E<br>Pflege-wissenschaft<br>Praxispartner | 2 |
| **2.31** | Forschungsziel: Bedarfe oder Probleme der Pflegepraxis | Forschungsziele des KI-Pflege-Projekts sind unbekannt oder orientieren sich nicht an den Bedarfen und Problemen der Pflegepraxis. | Erste Überlegungen zur Ableitung von Forschungszielen aus Praxisbedarfen werden angestellt. | Forschungsziele werden zunehmend aus spezifischen Praxisbedarfen abgeleitet, jedoch noch nicht systematisch oder nicht unter Einbezug eines theoretischen, empirischen und erfahrungsbasierten Ansatzes. | Forschungsziele werden systematisch aus den Bedarfen der Pflegepraxis unter Einbezug eines theoretischen, empirischen und erfahrungsbasiertem Ansatz unter Einbezug von Stakeholder:innen der Pflegepraxis abgeleitet und regelmäßig überprüft. | Forschungsziele sind vollständig und kontinuierlich unter Einbezug eines theoretischen, empirischen und erfahrungsbasiertem Ansatz unter Einbezug von Stakeholder:innen der Pflegepraxis an den Bedarfen der Pflegepraxis ausgerichtet und werden proaktiv angepasst. | Trifft nicht zu. | KI F&E<br>Pflege-wissenschaft<br>Praxispartner | 3 |
| **2.32** | Praktischer Nutzen und Mehrwert des KI-Systems | Kein praktischer Nutzen oder Mehrwert durch das KI-System erkennbar oder der praktische Nutzen oder Mehrwert sind unbekannt. Das KIP-Projekt ist überwiegend theoretisch. | Der potenzielle Nutzen des KI-Systems wird erkannt, aber konkrete Indikatoren oder Kriterien für die Abbildung des Nutzens und Mehrwerts sind unklar. Erste Schritte zur Bewertung des Mehrwerts werden unternommen aber nicht konsequent erfasst und dokumentiert. | Das KI-System weist einen erkennbaren praktischen Nutzen auf, der durch konkrete Indikatoren oder Kriterien abgebildet wird. Allerdings werden Endpunkte nur in Teilen erfasst oder Nutzen nur in Teilen realisiert. | Der praktische Nutzen des KI-Systems ist klar definiert und wird in dem KIP-Projekt systematisch umgesetzt und nachgewiesen. | Das KI-System generiert signifikanten praktischen Nutzen und Mehrwert, der mittels explizierter Kriterien und Indikatoren kontinuierlich gemessen und optimiert wird. | Trifft nicht zu. | Pflege-wissenschaft<br>Praxispartner<br>KI F&E | 3,5 |

ProKIP
Gefördert durch:
Bundesministerium für Forschung, Technologie und Raumfahrt

| Dimension 2<br>Prozessuale und translationale Voraussetzungen und Aspekte | | | | | | | | | |
|---|---|---|---|---|---|---|---|---|---|
| **Attribut-Nummer** | **Attribute** | **Stufe 1 (initial)** | **Stufe 2 (erkundend)** | **Stufe 3 (entschlossen)** | **Stufe 4 (gesteuert)** | **Stufe 5 (optimiert)** | **Trifft nicht zu** | **Bewertung durch** | **Quelle** |
| **2.33** | **Umsetzung realistischer, Feld-erprobter Projekte vor "Grand Vision" Projekten** | Keine Feldversuche. KIP-Projekte sind überwiegend visionär, aber realitätsfern. | Erste Schritte zur Umsetzung realistischer, praxisnaher KIP-Projekte neben visionären Ansätzen. | Praxisnahe KIP-Projekte werden durchgeführt, die jedoch noch nicht vollständig erprobt sind. | KIP-Projekte sind sowohl praxisnah als auch visionär, mit regelmäßigen Feldversuchen zur Validierung. | Es besteht eine ausgewogene Balance zwischen praxisnahen, feld-erprobten und visionären KIP-Projekten, die optimal aufeinander abgestimmt sind. | Trifft nicht zu. | Pflege-wissenschaft Praxispartner KI F&E | 3,5 |
| **2.34** | Strategien für die Partizipation und Information von Stakeholder:innen | Keine Strategien für die Partizipation und Information von Stakeholder:innen im KIP-Projekt oder Strategien sind unbekannt. | Erste Schritte zur Einbindung von Stakeholder:innen im KIP-Projekt werden unternommen, aber ohne systematischen Ansatz. | Stakeholder:innen werden aktiv in das KIP-Projekt einbezogen, jedoch nicht konsistent (etwa nur einmalig in kleiner Anzahl) oder nicht umfassend informiert. | Es existieren klare und strukturierte Strategien zur umfassenden und wiederholten Information und Partizipation von Stakeholder:innen im KI-Pflege-Projekt. | Strategien zur Stakeholder:innen-Partizipation sind im KIP-Projekt vollständig integriert und optimiert, mit regelmäßiger Anpassung an neue Anforderungen (etwa Erweiterung des einzubeziehenden Personenkreis oder Umsetzung ursprünglich nicht geplanter Formate zur Beteiligung und Information). | Trifft nicht zu. | Pflege-wissenschaft Praxispartner KI F&E | 5 |
| **2.35** | Strategien zur Vertrauensbildung, Abstimmung von Erwartungen und zum Abbau von Vorbehalten | Keine Strategien zur Vertrauensbildung oder zum Abbau von Vorbehalten gegenüber KI-Systemen im KIP-Projekt oder Strategien sind unbekannt. | Erste Ansätze zur Vertrauensbildung und Abstimmung von Erwartungen werden entwickelt, aber nicht systematisch und konsequent umgesetzt. | Vertrauensbildung und Erwartungsmanagement sind Teil der Projektplanung, aber noch nicht durchgängig systematisiert und integriert (etwa Verantwortlichkeit nur bei einem Projektpartner angesiedelt). | Strategien zur Vertrauensbildung und zum Abbau von Vorbehalten im KIP-Projekt sind festegelgt, dokumentiert und werden systematisch umgesetzt. | Vollständig integrierte und optimierte Strategien zur Vertrauensbildung werden umgesetzt, mit kontinuierlicher Anpassung und Verbesserung im Projektverlauf. | Trifft nicht zu. | Pflege-wissenschaft Praxispartner KI F&E | 6 |
| **2.36** | Reflexion der Bedeutung der Einbindung von Menschen als vermittelnde Instanz zwischen KI-System und Handlung und gegebenenfalls daraus resultierender Entwicklungskonsequenzen | Keine Reflexion über die Rolle von Menschen als vermittelnde Instanz zwischen KI-System und Handlung im KIP-Projekt. | Erste Überlegungen zur Rolle von Menschen in der Interaktion mit KI-Systemen, projektinterne Diskussion, aber noch keine konkreten Maßnahmen die sich in der Umsetzung des Projektes widerspiegeln. | Die Rolle von Menschen als vermittelnde Instanz wird anerkannt, jedoch nur teilweise in Maßnahmen übersetzt. Entsprechend gering ist der Einfluss auf die Entwicklung des KI-Systems. | Die Bedeutung menschlicher Vermittlung in der Interaktion mit KI wird umfassend reflektiert und systematisch in die Projektumsetzung und Entwicklung der KI-Systeme integriert. | Menschliche Vermittlung wird als zentraler Bestandteil der KI-Implementierung betrachtet und kontinuierlich optimiert. | Trifft nicht zu. | KI F&E Pflege-wissenschaft Praxispartner | 5 |

| Dimension 2<br>Prozessuale und translationale Voraussetzungen und Aspekte | | | | | | | | | |
|---|---|---|---|---|---|---|---|---|---|
| **Attribut-Nummer** | **Attribute** | **Stufe 1 (initial)** | **Stufe 2 (erkundend)** | **Stufe 3 (entschlossen)** | **Stufe 4 (gesteuert)** | **Stufe 5 (optimiert)** | **Trifft nicht zu** | **Bewertung durch** | **Quelle** |
| **2.37** | Strategien für eine langfristige externe Begleitung und Evaluation des KI-Einsatzes | Keine langfristigen Strategien für die externe Begleitung oder Evaluation des KI-Einsatzes vorhanden oder Überlegungen zu Strategien für die externe Begleitung oder Evaluation des KI-Einsatzes sind vorhanden, aber noch nicht langfristig gedacht. | Erste Überlegungen zur langfristigen, externen Begleitung und Evaluation, aber noch keine systematische Planung oder Konkretisierung. | Externe Begleitung und Evaluation erfolgen teilweise, aber noch nicht durchgängig und kontinuierlich. | Langfristige externe Begleitung und Evaluation sind etabliert und werden regelmäßig durchgeführt. | Die externe Begleitung und Evaluation sind vollständig integriert, systematisch und optimiert, mit kontinuierlicher Anpassung. | Trifft nicht zu. | KI F&E | 2 |
| **2.38** | Strategien für eine langfristige externe Begleitung: Software/ Hardware: Updates | Keine Strategien für Software-/Hardware-Updates des KI-Systems vorhanden. | Erste Überlegungen zur Entwicklung einer Strategie für Updates, aber noch nicht oder nur sehr eingeschränkt umgesetzt. | Strategien für Software-/Hardware-Updates sind festgelegt und teilweise implementiert, aber nicht durchgängig optimiert. | Umfassende Strategien für regelmäßige Software-/Hardware-Updates existieren, die jedoch noch Verbesserungspotenzial haben. | Vollständig integrierte und optimierte Strategien für Software-/Hardware-Updates, die kontinuierlich überprüft und angepasst werden. | Trifft nicht zu. | KI F&E | 2 |
| **2.39** | Strategien für eine langfristige externe Begleitung: Software/ Hardware: Upgrades | Keine Strategien für Software-/Hardware-Upgrades des KI-Systems vorhanden. | Erste Überlegungen zur Entwicklung einer Strategie für Upgrades, aber noch nicht oder nur sehr eingeschränkt umgesetzt. | Strategien für Software-/Hardware-Upgrades sind festgelegt und teilweise implementiert, aber nicht durchgängig optimiert. | Umfassende Strategien für regelmäßige Software-/Hardware-Upgrades existieren, die jedoch noch Verbesserungspotenzial haben. | Vollständig integrierte und optimierte Strategien für Software-/Hardware-Upgrades, die kontinuierlich überprüft und angepasst werden. | Trifft nicht zu. | KI F&E | 3 |
| **2.40** | Strategien für eine langfristige externe Begleitung: Software/ Hardware: Maintenance | Keine Strategien für die Wartung und Maintenance von Software und Hardware des KI-Systems vorhanden. | Erste Ansätze zur Strategieentwicklung für Wartung und Maintenance von Software und Hardware, aber noch nicht vollständig umgesetzt. | Strategien für Wartung und Maintenance von Software und Hardware sind teilweise implementiert, aber nicht durchgängig optimiert. | Es existieren umfassende Strategien für die regelmäßige Wartung und Maintenance von Software und Hardware, die jedoch noch Verbesserungspotenzial haben. | Vollständig integrierte und optimierte Strategien für Wartung und Maintenance von Software und Hardware, die kontinuierlich überprüft und angepasst werden. | Trifft nicht zu. | KI F&E | 3 |

ProKIP

Gefördert durch:
Bundesministerium für Forschung, Technologie und Raumfahrt

| Dimension 3<br>Technische Voraussetzungen und Aspekte | | | | | | | | | |
|---|---|---|---|---|---|---|---|---|---|
| **Attribut-Nummer** | **Attribute** | **Stufe 1 (initial)** | **Stufe 2 (erkundend)** | **Stufe 3 (entschlossen)** | **Stufe 4 (gesteuert)** | **Stufe 5 (optimiert)** | **Trifft nicht zu** | **Bewertung durch** | **Quelle** |
| **3.1** | Angliederung an existierende Dateninfrastrukturen und -plattformen | Keine Anbindung des KI-Systems an existierende Dateninfrastrukturen oder -plattformen, wie den European Health Data Space (EHDS). | Erste Anbindungen des KI-Systems an Dateninfrastrukturen werden geprüft, aber noch nicht umfassend umgesetzt. | Teilweise Anbindung an bestehende Dateninfrastrukturen, aber es gibt noch Integrationsthemen. | Umfassende Anbindung an existierende Dateninfrastrukturen, die weitgehend gut integriert sind. | Vollständig optimierte Anbindung an Dateninfrastrukturen, die nahtlos und effizient arbeitet und regelmäßig überprüft wird. | Trifft nicht zu. | KI F&E | 2 |
| **3.2** | Nutzung technischer Interoperabilitätsstandards und Nomenklaturen | Keine Nutzung von Interoperabilitätsstandards und Nomenklaturen. | Erste Schritte zur Nutzung und Berücksichtigung von Interoperabilitätsstandards und Nomenklaturen bei der Entwicklung des KI-Systems im KIP-Projekt, aber noch nicht vollständig implementiert. | Interoperabilitätsstandards und Nomenklaturen werden in Teilen für die Entwicklung des KI-Systems im KIP-Projekt berücksichtigt und genutzt, aber nicht durchgängig. | Weitgehende Nutzung und Integration von Interoperabilitätsstandards und Nomenklaturen bei der Entwicklung des KI-Systems im KIP-Projekt. | Vollständige und optimierte Nutzung Interoperabilitätsstandards und Nomenklaturen bei der Entwicklung des KI-Systems im KIP-Projekt, die regelmäßig aktualisiert und in allen relevanten Bereichen angewendet werden. | Trifft nicht zu. | KI F&E<br>Pflege-wissenschaft | 2,3,5 |
| **3.3** | IT-Sicherheit: Absicherung kritischer Infrastruktur und Verschlüsselungstechniken: verfügbare Cyber Security [beim Praxispartner] | Keine spezifischen Maßnahmen zur Absicherung der Infrastruktur oder zur Anwendung von Verschlüsselungstechniken [beim Praxispartner]. | Erste Sicherheitsmaßnahmen und Verschlüsselungstechniken werden [beim Praxispartner] implementiert, aber noch lückenhaft. | Sicherheitspraktiken und Verschlüsselungstechniken sind teilweise etabliert, jedoch besteht noch Verbesserungspotenzial. | Weitgehende Sicherheitsmaßnahmen und modernste Verschlüsselungstechniken sind implementiert und regelmäßig überprüft. | Vollständig integrierte, hochmoderne Sicherheitsinfrastruktur, die kontinuierlich optimiert und überwacht wird. | Trifft nicht zu. | KI F&E<br>Praxispartner | 3,5 |
| **3.4** | IT-Sicherheit: Sicherheitszertifizierungen | Keine Sicherheitszertifizierungen vorhanden. | Erste Schritte zur Erlangung von Sicherheitszertifizierungen werden unternommen. | Einige Sicherheitszertifizierungen wurden erreicht, aber nicht alle relevanten Bereiche sind abgedeckt. | Weitgehende Sicherheitszertifizierungen sind erreicht und werden regelmäßig aktualisiert. | Vollständig optimierte und auf dem neuesten Stand gehaltene Sicherheitszertifizierungen, die alle relevanten Bereiche abdecken. | Trifft nicht zu. | KI F&E | 5,6 |
| **3.5** | Praxispartner: Digitale Infrastruktur: Technische Infrastruktur | Die technische Infrastruktur [beim Praxispartner] ist unbekannt oder ist unzureichend für KI-Pflege-Projekte. | Erste Schritte zur Verbesserung der technischen Infrastruktur [beim Praxispartner], aber noch keine hinreichende Umsetzung. | Eine grundlegende technische Infrastruktur für KIP-Projekte ist [beim Praxispartner] vorhanden, aber es gibt noch Engpässe und Einschränkungen. | Die technische Infrastruktur [beim Praxispartner] ist gut entwickelt und unterstützt die meisten KI-Anwendungen effektiv. | Die technische Infrastruktur [beim Praxispartner] ist hochmodern, vollständig integriert und optimiert für den KI-Einsatz. | Trifft nicht zu. | Praxispartner mit IT-Fach-personal<br>KI F&E | 2,3,5 |

Gefördert durch:
Bundesministerium für Forschung, Technologie und Raumfahrt

| Dimension 3<br>Technische Voraussetzungen und Aspekte | | | | | | | | | |
|---|---|---|---|---|---|---|---|---|---|
| **Attribut-Nummer** | **Attribute** | **Stufe 1 (initial)** | **Stufe 2 (erkundend)** | **Stufe 3 (entschlossen)** | **Stufe 4 (gesteuert)** | **Stufe 5 (optimiert)** | **Trifft nicht zu** | **Bewertung durch** | **Quelle** |
| **3.6** | IT-Infrastruktur: KI Compute: Hardware | Keine dedizierte KI Hardware vorhanden. | Dedizierte KI Hardware ist vorhanden, aber noch nicht angebunden, etwa an Datenschnittstellen. | Dedizierte KI Hardware KI Hardware ist vorhanden und angebunden (etwa an Datenschnittstelle). Es ist möglich Daten für Modellentwicklung in die Laufzeitumgebung zu transferieren. | Dezidierte KI Hardware ist vorhanden und angebunden. Standardisierte Schnittstellen sind vorhanden, um Daten zu transferieren und Modellvorhersagen über Schnittstellen anzubieten. Kontinuierliche Testung und Wartung des KI Systems ist möglich. | Vollständig optimierte und auf dem neuesten Stand gehaltene dezidierte (Hard- und Software) Laufzeitumgebungen sind vorhanden. Modellentwicklung und -wartung entsprechend moderner CI/CD Komponenten ist implementiert. | Trifft nicht zu. | KI F&E | PROKIP |

| Dimension 4<br>Soziale und ethische Voraussetzungen und Aspekte | | | | | | | | | |
|---|---|---|---|---|---|---|---|---|---|
| **Attribut-Nummer** | **Attribute** | **Stufe 1 (initial)** | **Stufe 2 (erkundend)** | **Stufe 3 (entschlossen)** | **Stufe 4 (gesteuert)** | **Stufe 5 (optimiert)** | **Trifft nicht zu** | **Bewertung durch** | **Quelle** |
| **4.1** | Ethikvotum | Ein Ethikvotum für das KIP-Projekt ist entweder noch nicht vorbereitet worden oder gar nicht vorgesehen oder es ist unbekannt, ob oder welche Art eines Ethikvotums notwendig ist. | Erste Schritte zur Einholung eines Ethikvotums für das KIP-Projekt wurden unternommen, diese sind aber noch nicht abgeschlossen, sodass noch kein positives Votum einer Ethikkommission vorliegt. Oder die Zuständigkeiten bei der Erstellung und beantragung des Ethikvotums sind nicht im Projektverbund abgestimmt. | Ethikvoten werden für einige Projektschritte eingeholt, sind aber nicht systematisch im Projektverbund abgestimmt (wodurch Doppel- oder Mehrarbeit im Prozess der Antragsstellung bei einer Ethikkommission entsteht). | Ein positives Ethikvotum ist für alle relevanten Projektschritte vorhanden und wird bei Bedarf auch erneuert/erweitert. Zuständigkeiten und Verfahren für den Umgang mit Ergänzungen (Amendments) sind geklärt und allen Projektbetiligten bekannt. | Ein positives Ethikvotum ist für alle relevanten Projektschritte vorhanden und Ergebnis eines vollständig im KIP-Projekt integrierten und geplanten Verfahrens zur Einholung und Überprüfung von Ethikvoten. | Trifft nicht zu. | KI F&E Pflege-wissenschaft | 2 |
| **4.2** | Informierte Einwilligung (Informed Consent) und Andauernde Einwilligung (Ongoing Consent): Möglichkeiten und Grenzen eines Informed oder Ongoing Consent und alternative Lösungen | Keine Strategien oder Überlegungen zu Informed Consent oder alternativen Lösungen im KIP-Projekt vorhanden oder Stategien sind unbekannt.<br>Ob und wie ein Ongoing Consent im KIP-Projekt umgesetzt werden soll ist unklar. | Erste Ansätze zum Einholen eines Informed Consent oder alternative Lösungen oder eines Ongoing Consent werden entwickelt, sind aber noch nicht vollständig festgelegt oder umgesetzt. | Informed Consent oder Ongoing Consent werden überwiegend berücksichtigt, es gibt jedoch noch Lücken (z.B. nicht in jedem Projekt oder jeder Projektphase, in der es sinnvoll wäre). | Umfassende Strategien für Informed Consent oder Ongoing Consent sind etabliert, einschließlich alternativer Lösungen, die regelmäßig dokumentiert und überprüft werden. | Es gibt vollständig optimierte und flexible Strategien für Informed und Ongoing Consent, die kontinuierlich angepasst und weiterentwickelt werden. | Trifft nicht zu. | KI F&E Pflege-wissenschaft | 2 |
| **4.3** | Möglichkeiten und Grenzen von zentralen und dezentralen Methoden zur Sicherstellung der Privatsphäre von Studienteilnehmenden*<br>(*Studienteilnehmende sind alle Personen, von denen im KIP-Projekt Daten zur Entwicklung oder Evaluation des KI-Systems erhoben werden. Auch Pflegefachpersonen, die z.B. an Fokusgruppen teilnehmen, sind Studienteilnehmende.) | Keine Methoden zur Sicherstellung der Privatsphäre von Studienteilnehmenden im KIP-Projekt vorhanden oder Methoden sind unbekannt. | Erste Methoden zur Sicherstellung der Privatsphäre werden entwickelt, aber noch nicht umfassend umgesetzt. | Zentrale und dezentrale Methoden zur Sicherstellung der Privatsphäre sind teilweise implementiert, aber noch nicht optimiert. | Umfassende Methoden zur Sicherstellung der Privatsphäre sind etabliert und werden regelmäßig überprüft. | Es gibt vollständig integrierte und optimierte Methoden zur Sicherstellung der Privatsphäre, die regelmäßig aktualisiert werden. | Trifft nicht zu. | KI F&E Pflege-wissenschaft | 2 |

| Dimension 4<br>Soziale und ethische Voraussetzungen und Aspekte | | | | | | | | | |
|---|---|---|---|---|---|---|---|---|---|
| **Attribut-Nummer** | **Attribute** | **Stufe 1 (initial)** | **Stufe 2 (erkundend)** | **Stufe 3 (entschlossen)** | **Stufe 4 (gesteuert)** | **Stufe 5 (optimiert)** | **Trifft nicht zu** | **Bewertung durch** | **Quelle** |
| **4.4** | Auseinandersetzung mit ethisch-normativen Wertorientierungen des Feldes und einzelner Praxispartner | (Noch) Keine Auseinandersetzung mit ethischen Wertorientierungen im KIP-Projekt stattfindend. | Erste Überlegungen dazu, wie die Auseinandersetzung mit ethischen Wertorientierungen im KIP-Projekt stattfinden soll, aber diese sind noch nicht konkret und werden auch noch nicht systematisch umgesetzt. | Methoden zur Auseinandersetzung mit ethischen Wertorientierungen sind im KIP-Projekt vorgesehen und wurden bereits konkretisiert, sie werden aber nur teilweise und noch nicht systematisch umgesetzt und schließen einige Stakeholder:innen aus. | Im KIP-Projekt erfolgt regelmäßig und systematisch eine konkrete Auseinandersetzung mit ethischen Wertorientierungen mit allen Stakeholder:innen, die auch dokumentiert wird. | Im KIP-Projekt erfolgt regelmäßig und systematisch eine konkrete Auseinandersetzung mit ethischen Wertorientierungen mit allen Stakeholder:innen, die im Projektverlauf bei Bedarf angepasst wird. | Trifft nicht zu. | KI F&E<br>Pflege-wissenschaft<br>Praxispartner | 2,5,6 |
| **4.5** | Reflexion von Auswirkungen des KI-Einsatzes auf die direkte Arbeitsumgebung von Pflegefachpersonen | (Noch) Keine Reflexion über die Auswirkungen des KI-Einsatzes auf die Arbeitsumgebung im KIP-Projekt stattfindend. | Erste Überlegungen dazu, wie die Reflexion der Auswirkungen des KI-Einsatzes auf die Arbeitsumgebung im Projekt erfolgen soll, werden angestellt, sind aber noch nicht konkret und werden auch noch nicht systematisch umgesetzt. | Die Auswirkungen des KI-Einsatzes auf die Arbeitsumgebung werden im KIP-Projekt teilweise, aber noch nicht systematisch reflektiert. | Eine umfassende und methodisch systematische Reflexion der Auswirkungen des KI-Einsatzes auf die Arbeitsumgebung erfolgt im KIP-Projekt. | Eine umfassende und methodisch systematische Reflexion der Auswirkungen des KI-Einsatzes auf die Arbeitsumgebung wird im KIP-Projekt mit allen Stakeholder:innen umgesetzt, die im Projektverlauf bei Bedarf angepasst wird. | Trifft nicht zu. | KI F&E<br>Pflege-wissenschaft<br>Praxispartner | 2 |
| **4.6** | Reflexion von Auswirkungen des KI-Einsatzes auf das Berufsbild von Pflegefachpersonen | Der grundsätzliche Einfluss des KI-Einsatzes auf das Berufsbild von Pflegefachpersonen ist bekannt, aber die genauen Auswirkungen werden im KIP-Projekt nicht reflektiert. | Erste Reflexionen der Auswirkungen auf das Berufsbild werden im KIP-Projekt angestellt. Sie sind jedoch unsystematisch und bleiben ohne Konsequenz für die Entwicklung, Implementierung oder Evaluation des KI-Systems. | Die Auswirkungen des KI-Einsatzes auf das Berufsbild werden im KIP-Projekt systematisiert, bleiben aber weiterhin ohne weitere Konsequenz für die Entwicklung, Implementierung oder Evaluation des KI-Systems. | Im KIP-Projekt ist eine umfassende Reflexion der Auswirkungen auf das Berufsbild etabliert und nimmt auch Einfluss auf Entscheidungen bei der Entwicklung, Implementierung oder Evaluation des KI-Systems. | Im KIP-Projekt erfolgt eine vollständige Reflexion der Auswirkungen auf das Berufsbild , die auch Teil der Ergebnisdarstellung des Projektes ist. | Trifft nicht zu. | KI F&E<br>Pflege-wissenschaft<br>Praxispartner | 2 |
| **4.7** | Strategien für die systematische Erfassung von erwünschten und unerwünschten Wirkungen des KI Einsatzes | (Noch) keine Überlegungen oder Strategien zur Erfassung von Wirkungen des KI-Einsatzes im KIP-Projekt vorhanden. | Erste Überlegungen und Diskussionen zur Erfassung von Wirkungen des KI-Einsatzes im KIP-Projekt vorhanden. | Erste Strategien zur Erfassung von Wirkungen des KI-Einsatzes werden im KIP-Projekt entwickelt, sie sind aber noch nicht umfassend integriert und werden auch noch nicht methodisch systematisch umgesetzt. | Strategien zur Erfassung von Wirkungen des KI-Einsatzes sind im KIP-Projekt umfassend integriert und werden methodisch systematisch umgesetzt. | Strategien zur Erfassung von Wirkungen des KI-Einsatzes sind im KIP-Projekt umfassend integriert und werden methodisch systematisch umgesetzt; zudem werden die Strategien im Projektverlauf bei Bedarf angepasst. | Trifft nicht zu. | KI F&E<br>Pflege-wissenschaft | 2 |

| Dimension 4<br>Soziale und ethische Voraussetzungen und Aspekte | | | | | | | | | |
|---|---|---|---|---|---|---|---|---|---|
| **Attribut-Nummer** | **Attribute** | **Stufe 1 (initial)** | **Stufe 2 (erkundend)** | **Stufe 3 (entschlossen)** | **Stufe 4 (gesteuert)** | **Stufe 5 (optimiert)** | **Trifft nicht zu** | **Bewertung durch** | **Quelle** |
| **4.8** | Reflexion der Repräsentativität der genutzten Daten und der daraus abgeleiteten Bewertung des KI-Systems sowie der Übertragbarkeit der Ergebnisse des KIP-Projektes | Im KIP-Projekt werden die Repräsentativität der Daten und die Übertragbarkeit der Ergebnisse (noch) nicht reflektiert. | Im KIP-Projekt gibt es erste Reflexionen der Repräsentativität der Daten und der Übertragbarkeit der Ergebnisse, diese werden aber noch nicht systematisch dokumentiert. | Die Repräsentativität der Daten und die Übertragbarkeit der Ergebnisse werden im KIP-Projekt teilweise reflektiert und dokumentiert, es gibt aber noch Lücken. | Im KIP-Projekt werden die Repräsentativität der Daten und die Übertragbarkeit der Ergebnisse umfassend reflektiert sowie regelmäßig überprüft und dokumentiert. | Im KIP-Projekt werden die Repräsentativität der Daten und die Übertragbarkeit der Ergebnisse umfassend reflektiert sowie regelmäßig überprüft und dokumentiert; zudem werden Reflexion und Dokumentation kontinuierlich angepasst und weiterentwickelt. | Trifft nicht zu. | KI F&E Pflege-wissenschaft | 2 |
| **4.9** | Strategien, die die Transparenz und Erklärbarkeit von durch KI-Systeme getroffene Entscheidungen und Handlungsempfehlungen steigern | Im KIP-Projekt sind (noch) keine Strategien zur Steigerung der Transparenz und Erklärbarkeit vorhanden oder entsprechene Strategien sind unbekannt. | Erste Strategien zur Steigerung der Transparenz und Erklärbarkeit werden entwickelt, sind aber noch nicht umfassend umgesetzt. | Strategien zur Steigerung der Transparenz und Erklärbarkeit sind teilweise implementiert, aber noch nicht optimiert. | Optimierte Strategien zur Steigerung der Transparenz und Erklärbarkeit sind etabliert und werden regelmäßig überprüft. | Optimierte Strategien zur Steigerung der Transparenz und Erklärbarkeit sind umfassend etabliert und werden regelmäßig überprüft sowie kontinuierlich angepasst und weiterentwickelt. | Trifft nicht zu. | KI F&E Pflege-wissenschaft | 2,5 |

ProKIP
Gefördert durch:
Bundesministerium für Forschung, Technologie und Raumfahrt

| Dimension 4<br>Soziale und ethische Voraussetzungen und Aspekte | | | | | | | | | |
|---|---|---|---|---|---|---|---|---|---|
| **Attribut-Nummer** | **Attribute** | **Stufe 1 (initial)** | **Stufe 2 (erkundend)** | **Stufe 3 (entschlossen)** | **Stufe 4 (gesteuert)** | **Stufe 5 (optimiert)** | **Trifft nicht zu** | **Bewertung durch** | **Quelle** |
| **4.10** | Verantwortlicher Umgang mit Daten: Individueller Consent, Datenspende, Research Exemption oder Data Trusteeship*<br>* Wenn Daten gesammelt oder verwendet werden – zum Beispiel Daten aus der Pflegedokumentation, App-Nutzungsdaten oder Bewegungsdaten – sollte das verantwortungsvoll geschehen. Es geht darum, die Privatsphäre von Individuen zu schützen und fair mit ihren Informationen umzugehen. Dafür gibt es verschiedene Konzepte. Individueller Consent: Personen entscheiden selbst, ob und welche ihrer Daten verwendet werden dürfen. Datenspende: Personen stellen ihre Daten freiwillig für die Forschung zur Verfügung. Research Exemption (Forschungs-Ausnahme): In bestimmten Fällen ist die Nutzung der Daten durch Forschende auch ohne direkte Zustimmung möglich, etwa, wenn das öffentliche Interesse besonders groß ist und der Datenschutz trotzdem gewahrt bleibt. Beispiel: Gesundheitsdaten aus einem Krankenhaus werden anonymisiert verwendet, um eine Pandemie zu erforschen. data Trusteeship (Datentreuhänderschaft): Ein neutraler Dritter (z. B. eine Stiftung oder Behörde) verwaltet die Daten und gibt sie nur weiter, wenn bestimmte Regeln erfüllt sind | Im KIP-Projekt wird der verantwortliche Umgang mit Daten nicht reflektiert. | Im KIP-Projekt gibt es erste Reflexionen des verantwortlichen Umgangs mit Daten. Sie sind aber nicht systematisch und werden nicht dokumentiert. | Der verantwortliche Umgang mit Daten wird größtenteils systematisch reflektiert, es gibt aber noch Lücken. | Es gibt eine umfassende systematische Reflexion des verantwortlichen Umgangs mit Daten, die regelmäßig dokumentiert wird. | Es findet eine umfassende und optimierte Reflexion sowie Dokumentation des verantwortlichen Umgangs mit Daten statt, die kontinuierlich angepasst und weiterentwickelt wird. | Trifft nicht zu. | KI F&E | 2 |

Gefördert durch:
Bundesministerium für Forschung, Technologie und Raumfahrt

| Dimension 4<br>Soziale und ethische Voraussetzungen und Aspekte | | | | | | | | | |
|---|---|---|---|---|---|---|---|---|---|
| **Attribut-Nummer** | **Attribute** | **Stufe 1 (initial)** | **Stufe 2 (erkundend)** | **Stufe 3 (entschlossen)** | **Stufe 4 (gesteuert)** | **Stufe 5 (optimiert)** | **Trifft nicht zu** | **Bewertung durch** | **Quelle** |
| **4.11** | Beachtung der Positionierung und Wertorientierung der Pflege | Im KIP-Projekt wird die Wertorientierung der Pflege nicht reflektiert. | Im KIP-Projekt gibt es erste Reflexionen der Wertorientierung der Pflege. Sie sind aber nicht systematisch und werden nicht dokumentiert. | Die Wertorientierung der Pflege werden größtenteils systematisch reflektiert, es gibt aber noch Lücken. | Es gibt eine umfassende systematische Reflexionen der Wertorientierung der Pflege, die regelmäßig dokumentiert wird. | Es findet eine umfassende und optimierte Reflexion sowie Dokumentation der Wertorientierung der Pflege statt, die kontinuierlich angepasst und weiterentwickelt wird. | Trifft nicht zu. | KI F&E<br>Pflege-wissenschaft<br>Praxispartner | PROKIP |

| Dimension 5<br>Voraussetzungen und Aspekte des Community Building | | | | | | | | | |
|---|---|---|---|---|---|---|---|---|---|
| **Attribut-Nummer** | **Attribute** | **Stufe 1 (initial)** | **Stufe 2 (erkundend)** | **Stufe 3 (entschlossen)** | **Stufe 4 (gesteuert)** | **Stufe 5 (optimiert)** | **Trifft nicht zu** | **Bewertung durch** | **Quelle** |
| **5.1** | Technologischer Wissenstransfer | Keine Beteiligung des KIP-Projekts an einem technologischen Wissenstransfer. Technologischer Wissenstransfer ist nicht vorgesehen oder möglich. Aktivitäten für einen technologischen Wissenstransfer sind unbekannt. | Erste Schritte und Sondierungen für einen technologischen Wissenstransfer werden unternommen, aber noch nicht systematisch umgesetzt. | Technologischer Wissenstransfer wird teilweise durchgeführt, aber es gibt noch Lücken in der Konsistenz (z.B. unregelmäßige Beteiligung, abhängig vom Engagement einzelner Mitarbeitenden im KIP-Projekt) und Breite (z.B. nur regional begrenzt erfolgend oder nur ausgewählte aber nicht alle Zielgruppen ansprechend). | Umfassender technologischer Wissenstransfer ist etabliert und erfolgt regelmäßig. Die Vorgehensweise für einen technologischen Wissenstransfer wird regelmäßig aktualisiert und überprüft. Mindestens nationale Reichweite des technologischen Wissenstransfers. | Vollständig integrierter und optimierter technologischer Wissenstransfer findet statt, der kontinuierlich erweitert und an neue Erkenntnisse des KIP-Projektes und des KI-Systems angepasst wird. Nationale und internationale Reichweite des technologischen Wissenstransfers. | Trifft nicht zu. | KI F&E Pflege-wissenschaft | 2 |
| **5.2** | Beteiligung an Onlineaustausch | Keine Beteiligung der Partner im KIP-Projekt an Onlineaustausch durch Partizipation in Q&A-Netzwerken vorhanden. Keine Beteiligung von Programmierer:innen in einem geteilten Repository. | Erste Schritte zur Einrichtung eines Onlineaustauschs in Q&A-Netzwerken oder zur Beteiligung an einem geteilten Repository werden unternommen, aber noch nicht umfassend umgesetzt. | Onlineaustausch und Beteiligung an einem geteilten Repository sind teilweise etabliert, aber es gibt noch Verbesserungspotenzial (etwa Austausch und Beteiligung von dem Engagement von einzelnen Mitarbeitenden im KIP-Projekt abhängig oder unsystematische Beteiligung), | Umfassender Onlineaustausch und regelmäßige Partizipation in Q&A-Netzwerken und an einem geteilten Repository sind etabliert und werden aktiv von allen geeigneten Mitarbeitenden im KIP-Projekt genutzt. | Vollständig integrierte und optimierte Beteiligung an Onlineaustausch und einem geteilten Repository, die kontinuierlich erweitert und an aktuelle Erkenntnisse des KIP-Projekts oder des KI-Systems angepasst wird. | Trifft nicht zu. | KI F&E Pflege-wissenschaft Praxispartner | 2 |
| **5.3** | Strategische Partnerschaften | Keine bestehenden strategischen Partnerschaften im KIP-Projekt und bei einzelnen Projektpartnern oder strategische Partnerschaften sind unbekannt. | Erste Prüfung strategischer Partnerschaften, aber noch nicht vollständig geschlossen oder formalisiert. | Strategische Partnerschaften sind teilweise etabliert, aber nicht alle Partner im KIP-Projekt verfügen über strategische Partnerschaften. Insbesondere die Praxispartner weisen keine strategischen Partnerschaften außerhalb des Projektverbundes auf. | Umfassende strategische Partnerschaften sind etabliert. Auch die Praxispartner weisen strategische Partnerschaften außerhalb des Projektverbundes auf. Bestehende strategische Partnerschaften werden regelmäßig überprüft und vor allem national ausgebaut. | Erprobte und bewährte startegische Partnerschaften sind für alle Partner im KIP-Projekt etabliert. Bestehende strategische Partnerschaften werden kontinuierlich und auch international ausgebaut. | Trifft nicht zu. | KI F&E Pflege-wissenschaft Praxispartner | 6 |

Gefördert durch:
Bundesministerium für Forschung, Technologie und Raumfahrt

## References

1. Pumplun, L., Fecho, M., Wahl, N., Peters, F. & Buxmann, P. Adoption of Machine Learning Systems for Medical Diagnostics in Clinics: Qualitative Interview Study. J Med Internet Res 23, e29301 (2021).
2. Wolf-Ostermann, K., et al. Konzept zur Einbettung von KI-Systemen in der Pflege: Sondierungsprojekt zu KI in der Pflege (SoKIP). (Universität Bremen, Bremen, 2021).
3. Alami, H., et al. Organizational readiness for artificial intelligence in health care: insights for decision-making and practice. J Health Organ Manag (2020).
4. Weinert, L., Müller, J., Svensson, L. & Heinze, O. Perspective of Information Technology Decision Makers on Factors Influencing Adoption and Implementation of Artificial Intelligence Technologies in 40 German Hospitals: Descriptive Analysis. JMIR Medical Informatics 10(2022).
5. Chang, A., Implementation of Artificial Intelligence in Medicine, in Intelligence-Based Medicine. Artificial Intelligence and Human Cognition in Clinical Medicine and Healthcare. 2020, Academic Press: London, San Diego, Cambridge, Oxford. p. 397-412.
6. Wiljer, D. & Hakim, Z. Developing an Artificial Intelligence–Enabled Health Care Practice: Rewiring Health Care Professions for Better Care. Journal of Medical Imaging and Radiation Sciences 50, S8-S14 (2019).
7. Abuzaid, M.M., Elshami, W., Tekin, H. & Issa, B. Assessment of the Willingness of Radiologists and Radiographers to Accept the Integration of Artificial Intelligence Into Radiology Practice. Acad Radiol 29, 87-94 (2022).
8. Andersson, J., et al. Artificial intelligence and the medical physics profession - A Swedish perspective. Phys Med 88, 218-225 (2021).

ProKIP

Gefördert durch:

Bundesministerium für Forschung, Technologie und Raumfahrt

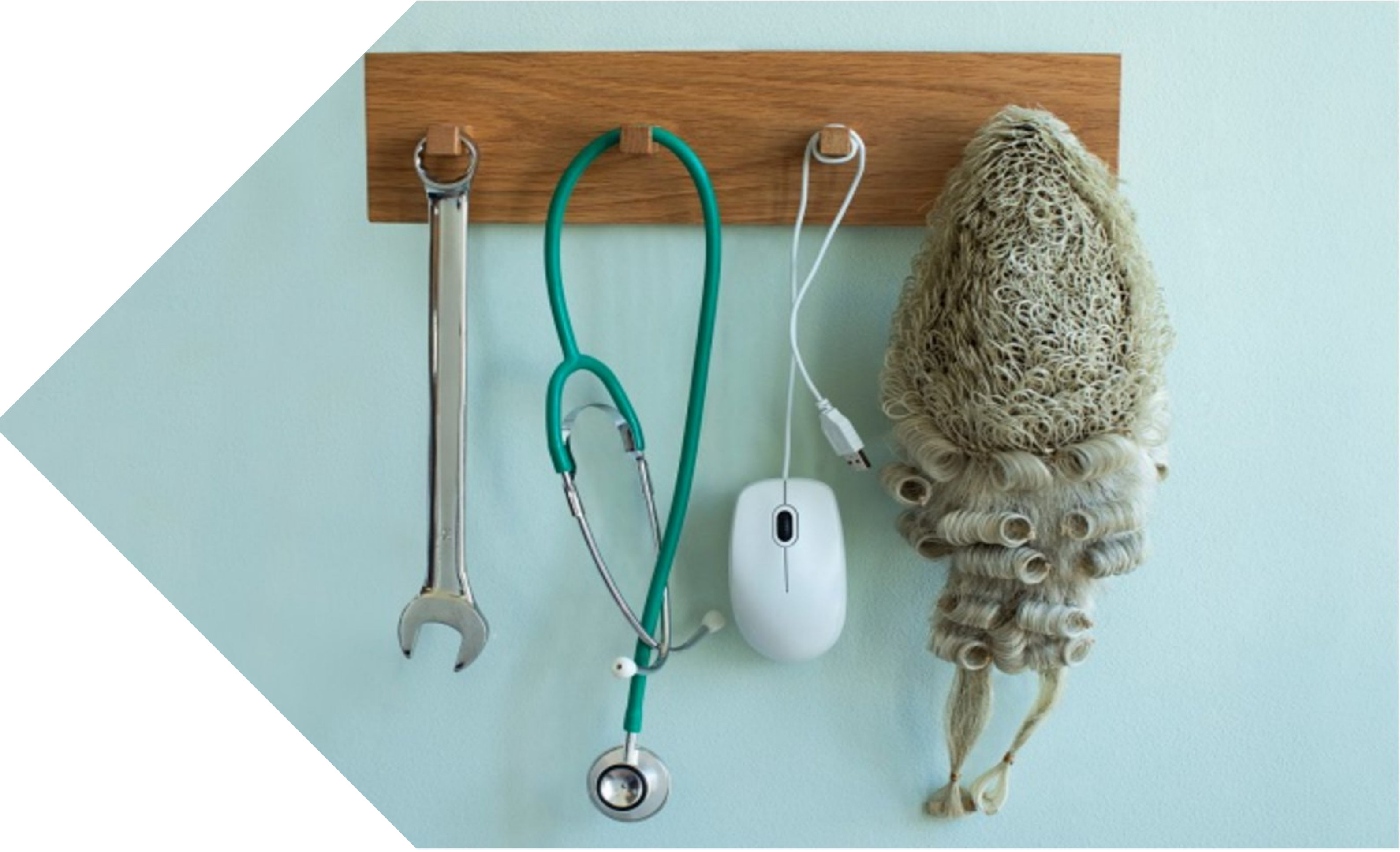

ProKIP – Prozessentwicklung und -begleitung zum KI-Einsatz in der Pflege – ist das wissenschaftliche Begleitprojekt in der vom Bundesministerium für Forschung, Technologie und Raumfahrt geförderten Bekanntmachung Repositorien und KI-Systeme im Pflegealltag nutzbar machen (Förderkennzeichen 16SV8835). Die Fördermittelgeber hatten keinen Einfluss auf die Studienplanung, Datenerhebung, -analyse und die Interpretation der Ergebnisse oder auf das Verfassen des Manuskripts.

**Autor*innen:**

Kathrin Seibert, Dominik Domhoff, Janissa Altona Sebastian Jäger, Felix Bießmann, Alessia Nowak, Rahel Gubser, Matthias Schulte-Althoff, Daniel Fürstenau, Jörg Pohle, Lea Bergmann, Kathi Beier, Dagmar Borchers, Karin Wolf-Ostermann

**Unter Mitarbeit von:** David Walter, Richard Dulzon

**Universität Bremen** - Fachbereich 11 Human- und Gesundheitswissenschaften - Institut für Public Health und Pflegeforschung - Grazer Str. 4, 28359 Bremen - https://www.uni-bremen.de/institut-fuer-public-health-und-pflegeforschung



Universität Bremen · vediso · BHT Berliner Hochschule für Technik · EINSTEIN CENTER Digital Future · Alexander von Humboldt Institut für Internet und Gesellschaft · CHARITÉ Universitätsmedizin Berlin
