# Supplementary material for: Readiness Assessment for AI in Nursing Care Projects: Multimethods Study
Source: JMIR Nurs. 2026 Jun 2;9:e84148. doi: 10.2196/84148 (PMC13229396; doi:10.2196/84148)
Supplement: Multimedia Appendix 3 [file nursing-v9-e84148-s003.pdf]

ProKIP

Gefördert durch:
Bundesministerium
für Forschung, Technologie
und Raumfahrt

AI Nursing Care Readiness Assessment (AINCRA)

User Manual

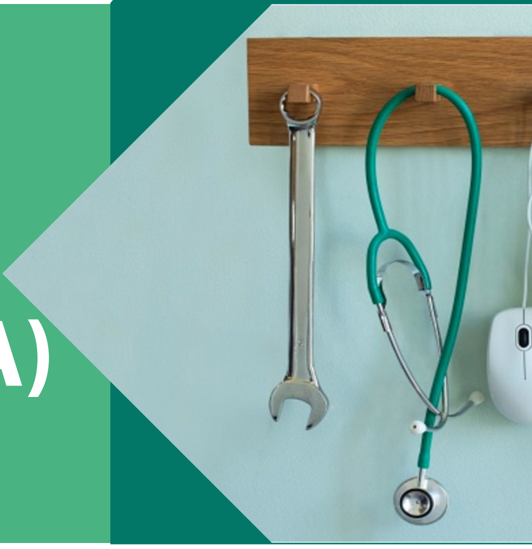

A result of the accompanying scientific research in the BMFTR funding program “Making Repositories and AI Systems Usable in Everyday Care.”

July 2025

Universität Bremen
vediso
BHT Berliner Hochschule für Technik
EINSTEIN CENTER Digital Future
Alexander von Humboldt Institut für Internet und Gesellschaft
CHARITÉ Universitätsmedizin Berlin

Gefördert durch:
Bundesministerium
für Forschung, Technologie
und Raumfahrt

Background of the AI-Nursing Care Readiness Assessment (AINCRA)

Artificial Intelligence (AI) is being used worldwide to support both direct physical nursing care and the organization of workflows and processes in the care sector. AI-based products are already available on the market – for example, voice-controlled nursing documentation, sensors for fall detection, software for intelligent shift and route planning, clinical decision support systems, or (virtual) activation and digital promotion of social participation (Seibert, Domhoff et al. 2021).

Research funding programs aim to evaluate the potential of AI for the nursing care sector. Research and development (R&D) projects focusing on AI in care (hereafter referred to as AI in care projects or AINC projects) are conducted to contribute to solving a major societal challenge: ensuring high-quality nursing care while an increasing imbalance between the care and support needs of rapidly aging populations and the availability of nursing and healthcare professionals makes it difficult to provide appropriate services.

AINC projects must take into account not only technical and regulatory requirements but also procedural, ethical, and social aspects of AI use. This applies across all project phases: planning, implementation, and evaluation. Project leads encounter diverse care settings with many possible AI use cases (e.g., AI for care in hospitals, nursing homes, outpatient care, or in education and training), each of which brings its own organizational logic and culture that influence the course of the project.

On behalf of the Federal Ministry for Research, Technology and Space (BMFTR), the University of Bremen, in cooperation with the Association for Digitization in Social Work (vediso), the Institute of Medical Informatics at Charité –University Medicine Berlin, the Berlin University of Applied Sciences and Technology, and the Alexander von Humboldt Institute for Internet and Society with the Einstein Center Digital Future, conducted the accompanying scientific research for the BMFTR funding program *Making Repositories and AI Systems Usable in Everyday Care*. As a result of this research, a maturity model was developed:
the **AI Nursing Care Readiness Assessment (AINCRA)**.

AINCRA is intended to support decision-makers in AINC projects, care institutions, and clinics in the planning, implementation, and evaluation of their projects by serving as a tool for reflection. With AINCRA, AINC project stakeholders can reflect on and proactively shape their approach to key requirements and aspects of these projects.

AINCRA covers topics such as the use of AI systems in nursing care, the representativeness of the data used, approaches to data sharing, the design of participatory processes, and the (care) ethical and professional practice implications of AI implementation.

Dieses Dokument enthält keine Überschriften. Wenden Sie ein Überschriftenformat auf den Text an, damit es im Inhaltsverzeichnis angezeigt wird.

Who is AINCRA intended for?

AINCRA is intended for:

- Project leads and responsible individuals in AI in care (AINC) projects
- Individuals in care facilities and clinics who wish to assess their organization's maturity level regarding the implementation of AINC projects

Project leads include academic project managers, staff members or project coordinators in care facilities and clinics, as well as individuals who, within the context of an AINC project, want or need to address specific areas (e.g., regulatory requirements) of AI readiness based on relevant skills (attributes).

What are AINC projects, AI systems, and clinical partners?

AI in Nursing Care Projects (AINC projects) are research and development (R&D) initiatives aimed at developing and studying an AI system (an AI-based nursing care technology) in collaboration with clinical partners from care facilities and clinics, or at implementing an existing AI system into everyday nursing practice. AINC projects may take the form of scientific research projects or be independently initiated and executed by care facilities and clinics, with or without involvement from a specific manufacturer or provider of AI systems.

AI systems are machine-based systems developed by humans, and designed to operate with varying levels of autonomy and to potentially adapt after deployment. To achieve explicit or implicit objectives, they infer – based on the input they receive – how to generate outputs such as predictions, content, recommendations, or decisions that can influence physical or virtual environments (European Commission 2025).

AI systems used in AINC projects may be based on machine learning (ML) methods, but they can also rely on expert systems or hybrid AI systems (which combine ML with expert systems).

Clinical partners in AINC projects include providers of outpatient, homecare, semi-residential, and residential long-term care, as well as hospitals.

Gefördert durch:
Bundesministerium für Forschung, Technologie und Raumfahrt

How was AINCRA developed?

AINCRA is the result of a multi-year development process. The following sources of knowledge contributed to its creation:

- International recommendations on the design and structure of maturity models (Akbarighatar et al., 2023; Becker et al., 2009; Lasrado et al., 2015),
- Published empirical research from global studies on challenges and enabling factors for AI in nursing care (N=292), as well as on AI readiness factors in healthcare and care organizations (N=7),
- Insights from workshops (N=21) and 14 interviews with German experts in nursing science, computer science, nursing education, nursing practice, and ethics – both with and without direct experience in AINC projects,
- Input from a dedicated workshop involving staff from current German AINC projects (N=13),
- Knowledge from think aloud interviews and group discussions with German experts from nursing science, computer science, nursing education, and care practice with AINC project experience (N=18),
- Experience of the study team gained from insights into AINC projects (N=8) funded under the BMFTR program *Making Repositories and AI Systems Usable in Everyday Care*.

Development of AINCRA followed a bottom-up approach (Lasrado et al., 2015). This approach begins by categorizing AI readiness factors into attributes and capabilities, then derives maturity levels and develops corresponding indicators. AINCRA was developed iteratively, with continuous integration of feedback from experts across disciplines – especially those with practical experience in planning, implementing, and evaluating AINC projects. AINCRA was developed within the European and German legal framework for AI in healthcare settings. AINCRA Users outside of Europe and Germany can adapt respective attributes to other regional or national requirements.

AINCRA was developed within the European and German legal framework for AI in healthcare settings. AINCRA Users outside of Europe and Germany can adapt respective attributes to other regional or national requirements.

Which dimensions and attributes are included in AINCRA?

AINCRA consists of five dimensions, which together encompass 69 subdimensions – these represent the attributes and capabilities required for AI nursing care readiness. Each dimension reflects a key area of action where AINC projects can influence project outcomes, and where clinical partners can assess their organization's maturity level in relation to the implementation of AINC projects.

At the end of AINCRA user manual, you'll find: a brief description of each of the five dimensions, an explanation of the maturity levels, and a detailed overview of all 69 subdimensions.

AINCRA uses a five-level maturity model, where each dimension and its subdimensions are assessed across five readiness levels: Level 1 represents the lowest degree of Ai nursing care readiness, level 5 represents the highest degree of readiness.

When, how, and by whom is AINCRA applied?

AINCRA can be applied on an ad hoc basis at any point in time – both during the planning phase and as part of the ongoing or final evaluation of AINC projects. A one-time assessment with AINCRA provides a snapshot of the current state of AI nursing care readiness at any given stage of the project. Repeated assessments allow for tracking the development of individual attributes over time.

AINCRA can be used either as a self-assessment or as an external evaluation tool. Ideally, the assessment is conducted by a inter-professional team. Depending on the domain and attribute, it is advisable that the assessment be carried out by individuals from the following fields:

- **AI Research and Development (AI R&D):** Those responsible for the technical functionality of the AI system.
- **Nursing Science:** Those involved in the nursing science oversight of the AINC project or contributing the nursing science perspective to the AI system development process.
- **Clinical Partners (including IT staff):** Those contributing the clinical application context of the AI system to the AINC project, i.e., people who are familiar with the staff, structures, and processes of the clinical partner, or who represent the clinical partner's perspective in the development process.

The recommended evaluators are indicated in AINCRA. AINCRA can be applied either for an entire AINC project or for a specific sub-area of a project (e.g., at the level of sub-dimensions).

How is AINCRA interpreted?

Due to the lack of an internationally established consensus on which prerequisites and aspects – with what weighting – most significantly influence the course and success of AINC projects, AINCRA deliberately avoids generating an overall score across individual or all dimensions. Instead, it encourages users to engage thoroughly with all specified dimensions and attributes.

The requirements and aspects included in AINCRA are based both on a comprehensive literature review and on insights from various workshops and interviews with experts. These factors have consistently been described as important for the success of AINC projects. In such projects – which typically involve inter-professional teams and require significant coordination and communication among participants – success is characterized by a smooth project trajectory leading to the piloting, testing, or implementation of an AI system in nursing practice, or to the achievement of the overarching project goals.
Therefore, the assessment result provides a snapshot of how well-positioned an AINC project is at the time of assessment in terms of addressing known prerequisites and factors that impact project progress and success.
During context analysis, project planning, and formative evaluation, project leads can use the assessment results to identify which prerequisites or aspects they wish – or need – to develop further, and which results they may choose to deprioritize. In summative evaluation, the results can offer insights into which factors may have had the greatest influence on the course and success of an AINC project.

In any project phase, the results of AINCRA can support the alignment, justification, and documentation of specific measures and strategies. In doing so, AINCRA promotes constructive and content-focused dialogue within AINC projects.

Overview: Five Dimensions of AINCRA

Dimension	Description
**Regulatory Requirements and Aspects**<br><br>*9 Attributes*	This dimension takes into account the necessary legal frameworks and data protection regulations that require an analysis of the data and an examination of data-sharing models in order to enable the use of AI systems in everyday nursing practice.
**Processual and Translational Requirements and Aspects**<br><br>*40 Attributes*	This dimension captures the human, material, temporal, and intangible resources available for AI in Nursing Care (AINC) projects in care facilities or hospitals, as well as their general and AI-specific level of digitalisation.<br><br>It also assesses the maturity of overarching strategies for AI, data governance, and IT governance, as well as plans for long-term external support and evaluation of AI implementation.<br><br>Engagement with the implementation of needs-based research and development of AI systems – focused on practical benefits and added value – as well as the existing AI-related knowledge and competencies within care facilities and clinics, is intended to support the seamless integration of AI systems into existing care processes.<br><br>This dimension also includes reflection on attitudes toward AI, acceptance of AI technologies, and trust in their use in daily nursing practice, in order to foster adoption and address concerns of those affected by the deployment of AI.
**Technical Requirements and Aspects**<br><br>*6 Attributes*	This dimension addresses the availability and functionality of the technical infrastructure, including aspects of data protection and data security, which are essential for reliable data use and analysis.<br><br>To ensure smooth, secure, and efficient data integration and exchange between different systems and stakeholders in everyday nursing practice, this dimension also considers the integration of AI systems into data infrastructures or data platforms, as well as the use of technical interoperability standards and nomenclatures.
**Social and Ethical Requirements and Aspects**<br><br>*11 Attributes*	This dimension encompasses ethical and methodological considerations related to voluntariness, privacy, fairness, and transparency, as well as the ethical and normative value orientations of the field and individual practice partners.<br><br>Engaging with the impacts of AI use at the micro, meso, and macro levels, along with a responsible approach to data handling, is intended to support the use of AI systems in everyday nursing practice.
**Community Building Requirements and Aspects**<br><br>*3 Attributes*	This dimension aims to promote a network that strengthens knowledge exchange and the collective advancement of AI systems in everyday nursing practice. Researchers, developers, and stakeholders from nursing practice and nursing management are involved in this network.

Overview: Levels of AINCRA

AINCRA enables an assessment of each dimension and its subdimensions using five maturity levels (Level 1 to Level 5). Level 1 represents the lowest, and Level 5 the highest degree of AI nursing care readiness.

The five levels, along with their corresponding indicators for assessment, are as follows:

**Level 1 (initial)**	**Level 2 (assessing)**	**Level 3 (determined)**	**Level 4 (managed)**	**Level 5 (optimised)**
The attribute assessed is …				
• unclear<br>• missing<br>• inadequate<br>• rejected<br>• ignored<br>• not/ never<br>○ known<br>○ consistent<br>○ coordinated<br>○ available<br>○ considered<br>○ taken into account<br>○ accepted<br>○ ready<br>○ recognised	• in planning<br>• under consideration<br>• sporadic<br>• outdated<br>• barely<br>○ available<br>○ present<br>• not/ never<br>○ complete<br>○ clarified<br>○ systematized<br>○ defined<br>○ documented<br>○ tested<br>○ evaluated<br>• hesitantly<br>○ accepted<br>○ ready<br>○ recognised	• regulated internally within the organization (internal standards)<br>• regulated internally within the project (internal standards)<br>• partially<br>○ systematised<br>○ available<br>○ present<br>• inconsistent<br>• not always<br>○ ensured<br>○ defined<br>○ coordinated<br>• known to some stakeholders<br>• irregular<br>• not yet<br>○ automatised<br>○ put into practice<br>○ started<br>○ documented<br>• partially<br>○ implemented<br>○ tested<br>○ evaluated<br>• increasingly<br>○ accepted<br>○ ready<br>○ recognised	• regulated across organizations (national standards)<br>• regulated across projects (national standards)<br>• continuously<br>○ available<br>○ accessible<br>○ ensured<br>○ systematised<br>○ communicated<br>• consistent<br>• involved<br>• integrated<br>• centralised<br>• regularly<br>○ updated<br>○ monitored<br>• known to all stakeholders<br>• largely<br>○ automatised<br>○ put into practice<br>○ implemented<br>○ documented<br>○ tested<br>○ evaluated<br>○ accepted<br>○ ready<br>○ recognised	• international standards<br>• available in real time<br>• optimised<br>• integrated<br>• efficient<br>• secure<br>• externally certified<br>• proven<br>• verifiable<br>• further developed<br>• continuously adapted<br>• complete/ fully<br>○ put into practice<br>○ implemented<br>○ documented<br>○ tested<br>○ evaluated<br>○ accepted<br>○ ready<br>○ recognised<br>• comprehensive

Depending on the AINC project, not all of the proposed attributes for assessment may apply. Therefore, it is always possible to select "Not applicable" as a rating.

Gefördert durch:
Bundesministerium für Forschung, Technologie und Raumfahrt

Overview: Subdimensions and Attributes of the AI Nursing Care Readiness Assessment (AINCRA)

Dimension	Attribute Number	Subdimension/ Attribute	(Joint) Assessment by (Rater Entity)	Sources for the Attribute
1 Regulatory Requirements and Aspects	1.1	**Analysis of the data set: Information content (despite anonymisation)**	AI R&D<br>Nursing Science<br>Clinical Partner	(Pumplun et al., 2021)
	1.2	**Analysis of the data set: Representativeness of training data**	AI R&D<br>Nursing Science	(Pumplun et al., 2021; Wolf-Ostermann et al., 2021)
	1.3	**Analysis of the data set: Quality**	AI R&D<br>Clinical Partner	(Pumplun et al., 2021; Wolf-Ostermann et al., 2021)
	1.4	**Analysis of the data set: Availability**	AI R&D<br>Clinical Partner	(Alami et al., 2020; Pumplun et al., 2021; Weinert et al., 2022; Wolf-Ostermann et al., 2021)
	1.5	**Analysis of the data set: Access**	AI R&D<br>Clinical Partner	(Alami et al., 2020; Chang, 2020; Wolf-Ostermann et al., 2021)
	1.6	**Data sharing models**	AI R&D	(Alami et al., 2020; Chang, 2020; Wolf-Ostermann et al., 2021)
	1.7	**EU Medical Device Regulation (EU MDR) (and/or, although phasing out, MPG)**<br>(Note: MPG = German Medical Devices Act)	AI R&D<br>Nursing Science<br>Clinical Partner	(Chang 2020; Wiljer & Hakim, 2019; Wolf-Ostermann et al., 2021)
	1.8	**EU GDPR and sector-specific laws**	AI R&D<br>Nursing Science<br>Clinical Partner	(Chang 2020; Wiljer & Hakim, 2019; Wolf-Ostermann et al., 2021)
	1.9	**EU AI Act**	AI R&D<br>Nursing Science<br>Clinical Partner	PROKIP

Dimension	Attribute Number	Subdimension/ Attribute	(Joint) Assessment by (Rater Entity)	Sources for the Attribute
2 Processual and Translational Requirements and Aspects	2.1	**Clinical Partner: Time Resources***<br>*Time resources may refer to leadership and nursing staff of the organisation, but also to other personnel (e.g., IT professionals or other occupational groups) who need to be planned accordingly.	Clinical Partner	(Weinert et al., 2022)
	2.2	**Clinical Partner: Personnel Resources*: Own staff position for implementing AI in the organisation**<br>*Personnel resources may refer to leadership and nursing staff of the organisation, but also to other personnel (e.g., IT professionals or other occupational groups) who need to be planned accordingly.	Clinical Partner	(Abuzaid et al., 2022)
	2.3	**Clinical Partner: Personnel Resources: Own staff position involved in AI research and development (R&D)**	Clinical Partner	(Abuzaid et al., 2022)
	2.4	**Clinical Partner: Personnel Resources: Available personnel resources for AINC projects and AI integration in the organisation**	Clinical Partner	(Weinert et al., 2022)
	2.5	**Clinical Partner: Personnel Resources: Available Data Scientists***<br>*Data Scientists use data analytics, machine learning, and statistics based on the clinical partner's data to improve nursing care, optimise operations, and support research. Clinical partners employing their own data scientists find it easier to utilise structured and unstructured data in the organisation for AINC projects.	Clinical Partner with IT staff AI R&D	(Chang, 2020)
	2.6	**Clinical Partner: Personnel Resources: Available Data Champions***<br>*Data Champions understand the types of data generated at the clinical partner, advocate for proper data handling, and mediate between nursing staff and IT. They ensure data is correct, complete, and up to date, identify and resolve data collection issues, enhance staff data literacy through training, and raise awareness about the importance of data in nursing care and research.	Clinical Partner with IT staff AI R&D	(Chang, 2020)
	2.7	**Clinical Partner: General Degree of Digitisation***<br>*Overall digitisation level of the clinical partner's organisation and its different departments and data types (e.g., HR data, patient/care data, administrative data, etc.)	Clinical Partner with IT staff AI R&D	(Chang, 2020; Pumplun et al., 2021; Weinert et al., 2022)
	2.8	**Clinical Partner: AI-specific Degree of Digitisation**	Clinical Partner with IT staff AI R&D	(Chang, 2020; Weinert et al., 2022)
	2.9	**Clinical Partner: Degree of Digitisation: Data Quality Standards**	Clinical Partner with IT staff AI R&D	(Pumplun et al., 2021)
	2.10	**Clinical Partner: Degree of Digitisation: Standard for Data Collection***<br>*Refers to a standard for collecting routine data generated in everyday care and organisational processes.	Clinical Partner with IT staff AI R&D	(Alami et al., 2020)

Dimension	Attribute Number	Subdimension/ Attribute	(Joint) Assessment by (Rater Entity)	Sources for the Attribute
2 Processual and Translational Requirements and Aspects	2.11	**Clinical Partner: Degree of Digitisation: Standard for Data Storage**	Clinical Partner with IT staff<br>AI R&D	(Alami et al., 2020)
	2.12	**Clinical Partner: Degree of Digitisation: Standard for Data Exchange**	Clinical Partner with IT staff<br>AI R&D	(Alami et al., 2020)
	2.13	**Clinical Partner: Degree of Digitisation – Standard for Ontological Representation***<br>*Ontological representation refers to the structured depiction of knowledge in a specific domain (e.g., medicine), to make data uniform, interpretable, and high-quality. A standard for ontological representation helps structure and standardise data semantically, making it understandable, comparable, and interoperable. A hospital, for example, may use different terms for the same illness across different documents: "heart attack," "myocardial infarction," "ICD-10: I21." An ontological representation would unify these terms under one standardised definition, allowing IT systems, healthcare professionals, and AI developers to clearly recognise they refer to the same condition.	Clinical Partner with IT staff<br>AI R&D	(Alami et al., 2020)
	2.14	**Clinical Partner: Willingness for Digital Transformation: Attitude and handling of AI in the organisation**	Clinical Partner with IT staff<br>AI R&D	(Alami et al., 2020; Chang, 2020)
	2.15	**Clinical Partner: Willingness for Digital Transformation: Support for AI from leadership and stakeholders***<br>*Stakeholders are all individuals or groups who influence the work climate in an organisation – such as managers, team leaders, employees, HR personnel, and sometimes external consultants – whose actions, decisions, and interactions shape the organisational culture, communication, trust, motivation, and overall work environment.	Clinical Partner	(Chang, 2020)
	2.16	**Clinical Partner: Willingness for Digital Transformation: Organisation's own AI strategy**	Clinical Partner	(Alami et al., 2020; Pumplun et al., 2021)
	2.17	**Clinical Partner: Willingness for Digital Transformation: Organisation's own data governance strategy***<br>*Data governance formalises decision rights, procedures, and controls to resolve conflicts of interest in data processing and sharing among involved actors. This requires maximizing the value of data and minimizing the risks depending on each stakeholder's perspective.	Clinical Partner	(Abuzaid et al., 2022; Alami et al., 2020; Chang, 2020; Pumplun et al., 2021; Weinert et al., 2022; Wiljer & Hakim, 2019)
	2.18	**Clinical Partner: Willingness for Digital Transformation: Organisation's own IT governance strategy***<br>*IT governance is the strategic framework ensuring that IT is used effectively, securely, and in compliance with regulations to support organisational goals.	Clinical Partner	(Alami et al., 2020)

Dimension	Attribute Number	Subdimension/ Attribute	(Joint) Assessment by (Rater Entity)	Sources for the Attribute
2 Processual and Translational Requirements and Aspects	2.19	**Clinical Partner: Technology Acceptance: Acceptance of AI by leadership, nursing staff, and other stakeholders***<br>*Stakeholders are all relevant groups other than patients or care recipients - this may include legal guardians, general practitioners, or consultative services depending on the AINC project. A stakeholder analysis should be part of every AINC project plan.	Clinical Partner	(Alami et al., 2020)
	2.20	**Clinical Partner: Technology Acceptance: Acceptance of AI by care recipients, patients, and their relatives**	Clinical Partner<br>Nursing Science	(Chang, 2020; Pumplun et al., 2021; Wiljer & Hakim, 2019)
	2.21	**Clinical Partner: Technology Acceptance: Acceptance of AI by staff representatives and their inclusion**	Clinical Partner<br>Nursing Science	(Pumplun et al., 2021; Wiljer & Hakim, 2019)
	2.22	**Clinical Partner: Expectations and concerns**	Clinical Partner<br>Nursing Science	PROKIP
	2.23	**Clinical Partner: Knowledge and Competencies: AI and digital skills education, training, and continuing education in the organisation**	Clinical Partner<br>Nursing Science<br>AI R&D	(Chang, 2020; Pumplun et al., 2021)
	2.24	**Clinical Partner: Knowledge and Competencies: Available AI knowledge among staff**	Clinical Partner	(Alami et al., 2020; Andersson et al., 2021)
	2.25	**Clinical Partner: Knowledge and Competencies: Real-time and/or predictive analytics**	Clinical Partner	(Andersson et al., 2021; Pumplun et al., 2021; Weinert et al., 2022; Wiljer & Hakim, 2019)
	2.26	**Clinical Partner: Knowledge and Competencies: Available methods for knowledge transfer within the organisation**	Clinical Partner	(Chang, 2020)
	2.27	**Clinical Partner: Intangible Assets: Availability and scope of intangible assets in the context of AI within the organisation***<br>*E.g., books, scholarly articles, awards, strategic partnerships, consultations, etc.) Intangible assets related to digitisation and digital competencies can also be valuable to clinical partners in AINC projects.	Clinical Partner	(Wiljer & Hakim, 2019)

Dimension	Attribute Number	Subdimension/ Attribute	(Joint) Assessment by (Rater Entity)	Sources for the Attribute
2 Processual and Translational Requirements and Aspects	2.28	**Clinical Partner: Financial Resources and Investments: Available financial resources for AINC projects and AI integration***<br>*These may also be part of general digitisation budgets or other budgets (e.g., innovation or inclusion budgets).	Clinical Partner	(Chang, 2020)
	2.29	**Financial Resources and Investments: Exploring alternative financing models for including clinical partners**	Clinical Partner	(Alami et al., 2020; Chang, 2020; Pumplun et al., 2021; Weinert et al., 2022)
	2.30	**Financial Resources and Investments: Management: sustainable alignment of resources and investments**	AI R&D<br>Nursing Science<br>Clinical Partner	(Wolf-Ostermann et al., 2021)
	2.31	**Research Objective: Needs or problems in nursing practice**	AI R&D<br>Nursing Science<br>Clinical Partner	(Alami et al., 2020)
	2.32	**Practical benefit and added value of the AI system**	AI R&D<br>Nursing Science<br>Clinical Partner	(Alami et al., 2020; Chang, 2020)
	2.33	**Focus on realistic, field-tested projects over "grand vision" projects**	Nursing Science<br>AI R&D Clinical Partner	(Alami et al., 2020; Chang, 2020)
	2.34	**Strategies for stakeholder participation and communication**	Nursing Science<br>AI R&D Clinical Partner	(Chang, 2020)
	2.35	**Strategies for trust-building, expectation alignment, and reducing concerns**	Nursing Science<br>AI R&D Clinical Partner	(Wiljer & Hakim, 2019)
	2.36	**Reflection on the importance of involving humans as mediators between AI systems and actions, and any resulting development implications**	Nursing Science<br>AI R&D Clinical Partner	(Chang, 2020)

Dimension	Attribute Number	Subdimension/ Attribute	(Joint) Assessment by (Rater Entity)	Sources for the Attribute
2 Processual and Translational Requirements and Aspects	2.37	**Strategies for long-term external support and evaluation of AI deployment**	AI R&D<br>Nursing Science<br>Clinical Partner	(Wolf-Ostermann et al., 2021)
	2.38	**Strategies for long-term external support: software/hardware: updates**	AI R&D	(Wolf-Ostermann et al., 2021)
	2.39	**Strategies for long-term external support: software/hardware: upgrades**	AI R&D	(Alami et al., 2020)
	2.40	**Strategies for long-term external support: software/hardware: maintenance**	AI R&D	(Alami et al., 2020)

Gefördert durch:
Bundesministerium für Forschung, Technologie und Raumfahrt

Dimension	Attribute Number	Subdimension/ Attribute	(Joint) Assessment by (Rater Entity)	Sources for the Attribute
3 Technical Requirements and Aspects	3.1	**Integration into Existing Data Infrastructures and Platforms**	AI R&D	(Wolf-Ostermann et al., 2021)
	3.2	**Use of Technical Interoperability Standards and Nomenclatures**	AI R&D<br>Nursing Science	(Alami et al., 2020; Chang, 2020; Wolf-Ostermann et al., 2021)
	3.3	**IT Security: Protection of Critical Infrastructure and Encryption Technologies (at the Clinical Partner)**	AI R&D<br>Clinical Partner	(Alami et al., 2020; Chang, 2020)
	3.4	**IT Security: Security Certifications**	AI R&D	(Chang, 2020; Wiljer & Hakim, 2019)
	3.5	**Clinical Partner: Digital Infrastructure: Technical Infrastructure**	AI R&D	(Alami et al., 2020; Chang, 2020; Wolf-Ostermann et al., 2021)
	3.6	**IT Infrastructure: AI Compute: Hardware**	AI R&D	PROKIP

Dimension	Attribute Number	Subdimension/ Attribute	(Joint) Assessment by (Rater Entity)	Sources for the Attribute
4 Social and Ethical Requirements and Aspects	4.1	**Ethics Vote**	AI R&D<br>Nursing Science	(Wolf-Ostermann et al., 2021)
	4.2	**Informed and Ongoing Consent (Including Alternatives)**	AI R&D<br>Nursing Science	(Wolf-Ostermann et al., 2021)
	4.3	**Centralised and Decentralised Methods for and Limitations of Ensuring Study Participant* Privacy**<br>*Study Participants are all individuals from whom data is collected in the AINC project to develop or evaluate an AI system. This also includes nurses and other health professionals, for example, those who participate in focus groups.	AI R&D<br>Nursing Science	(Wolf-Ostermann et al., 2021)
	4.4	**Engagement with Ethical-Normative Values of Nursing and Care, and Individual Clinical Partners**	AI R&D<br>Nursing Science<br>Clinical Partner	(Chang, 2020; Wiljer & Hakim, 2019; Wolf-Ostermann et al., 2021)
	4.5	**Reflection on Impact of the AI System on the Nursing Work Environment**	AI R&D<br>Nursing Science<br>Clinical Partner	(Wolf-Ostermann et al., 2021)
	4.6	**Reflection on the Impact of AI on the Nursing Profession**	AI R&D<br>Nursing Science<br>Clinical Partner	(Wolf-Ostermann et al., 2021)
	4.7	**Strategies for Systematic Assessment of Intended and Unintended Effects of AI**	AI R&D<br>Nursing Science	(Wolf-Ostermann et al., 2021)
	4.8	**Reflection on Data Representativeness and Transferability of Results of the AINC Project**	AI R&D<br>Nursing Science	(Wolf-Ostermann et al., 2021)
	4.9	**Strategies to Increase Transparency and Explainability of AI Decisions**	AI R&D<br>Nursing Science	(Chang, 2020; Wolf-Ostermann et al., 2021)

Dimension	Attribute Number	Subdimension/ Attribute	(Joint) Assessment by (Rater Entity)	Sources for the Attribute
4<br>Social and Ethical Requirements and Aspects	4.10	**Responsible Data Management: Individual Consent, Data Donation, Research Exemption or Data Trusteeship***<br>*When data are collected or used – for example, data from nursing documentation, app usage data, or movement data – this should be done responsibly. The aim is to protect individuals' privacy and handle their information fairly. There are various concepts for this:<br>Individual consent: People decide for themselves whether and which of their data may be used.<br>Data donation: People voluntarily provide their data for research purposes.<br>Research exemption: In certain cases, researchers may use data without direct consent, for example, when there is a particularly strong public interest and data protection is still ensured. Example: Health data from a hospital is anonymised and used to study a pandemic.<br>Data trusteeship: A neutral third party (e.g., a foundation or public authority) manages the data and only releases it when certain rules are met.	AI R&D	(Wolf-Ostermann et al., 2021)
	4.11	**Consideration of Values of Nursing and Positioning**	AI R&D<br>Nursing Science<br>Clinical Partner	PROKIP

Dimension	Attribute Number	Subdimension/ Attribute	(Joint) Assessment by (Rater Entity)	Sources for the Attribute
5 Community Building Requirements and Aspects	5.1	**Technological Knowledge Transfer**	AI R&D<br>Nursing Science	(Wolf-Ostermann et al., 2021)
	5.2	**Participation in Online Exchange**	AI R&D<br>Nursing Science<br>Clinical Partner	(Wolf-Ostermann et al., 2021)
	5.3	**Strategic Partnerships**	AI R&D<br>Nursing Science<br>Clinical Partner	(Wiljer & Hakim, 2019)

References

Abuzaid, M. M., Elshami, W., Tekin, H., & Issa, B. (2022). Assessment of the Willingness of Radiologists and Radiographers to Accept the Integration of Artificial Intelligence Into Radiology Practice. Acad Radiol, 29(1), 87-94. https://doi.org/doi:10.1016/j.acra.2020.09.014

Akbarighatar, P., Pappas, I., & Vassilakopoulou, P. (2023). A sociotechnical perspective for responsible AI maturity models: Findings from a mixed-method literature review. International Journal of Information Management Data Insights, 3(2). https://doi.org/10.1016/j.jjimei.2023.100193

Alami, H., Lehoux, P., Denis, J. L., Motulsky, A., Petitg, , C., Savoldelli, M., Rouquet, R., Gagnon, M. P., Roy, D., & Fortin, J. P. (2020). Organizational readiness for artificial intelligence in health care: insights for decision-making and practice. J Health Organ Manag. https://doi.org/doi:10.1108/jhom-03-2020-0074

Andersson, J., Nyholm, T., Ceberg, C., Almén, A., Bernhardt, P., Fransson, A., & Olsson, L. E. (2021). Artificial intelligence and the medical physics profession - A Swedish perspective. Phys Med, 88, 218-225. https://doi.org/doi:10.1016/j.ejmp.2021.07.009

Becker, J., Knackstedt, R., & Pöppelbuß, J. (2009). Developing Maturity Models for IT Management. Business & Information Systems Engineering, 1(3), 213-222. https://doi.org/10.1007/s12599-009-0044-5

Chang, A., Implementation of Artificial Intelligence in Medicine, in Intelligence-Based Medicine. Artificial Intelligence and Human Cognition in Clinical Medicine and Healthcare. 2020, Academic Press: London, San Diego, Cambridge, Oxford. p. 397-412.

Lasrado, L. A., Vatrapu, R., & Andersen, K. N. (2015). Maturity Models Development in IS Research: A Literature Review Selected Papers of the Informations Systems Research Seminar in Scandinavia, http://aisel.aisnet.org/iris2015/6

Pumplun, L., Fecho, M., Wahl, N., Peters, F., & Buxmann, P. (2021). Adoption of Machine Learning Systems for Medical Diagnostics in Clinics: Qualitative Interview Study. J Med Internet Res, 23(10), e29301. https://doi.org/doi:10.2196/29301

Seibert, K., D. Domhoff, D. Bruch, M. Schulte-Althoff, D. Furstenau, F. Biessmann and K. Wolf-Ostermann (2021). "Application Scenarios for Artificial Intelligence in Nursing Care: Rapid Review." J Med Internet Res 23(11): e26522.

Weinert, L., Müller, J., Svensson, L., & Heinze, O. (2022). Perspective of Information Technology Decision Makers on Factors Influencing Adoption and Implementation of Artificial Intelligence Technologies in 40 German Hospitals: Descriptive Analysis. JMIR Medical Informatics, 10(6). https://doi.org/doi:10.2196/34678

Wiljer, D., & Hakim, Z. (2019). Developing an Artificial Intelligence–Enabled Health Care Practice: Rewiring Health Care Professions for Better Care. Journal of Medical Imaging and Radiation Sciences, 50(4), S8-S14. https://doi.org/doi:10.1016/j.jmir.2019.09.010

Wolf-Ostermann, K., Fürstenau, D., Theune, S., Bergmann, L., Bießmann, F., Domhoff, D., Schulte-Althoff, M., & Seibert, K. (2021). Konzept zur Einbettung von KI-Systemen in der Pflege: Sondierungsprojekt zu KI in der Pflege (SoKIP).

Gefördert durch:
Bundesministerium für Forschung, Technologie und Raumfahrt

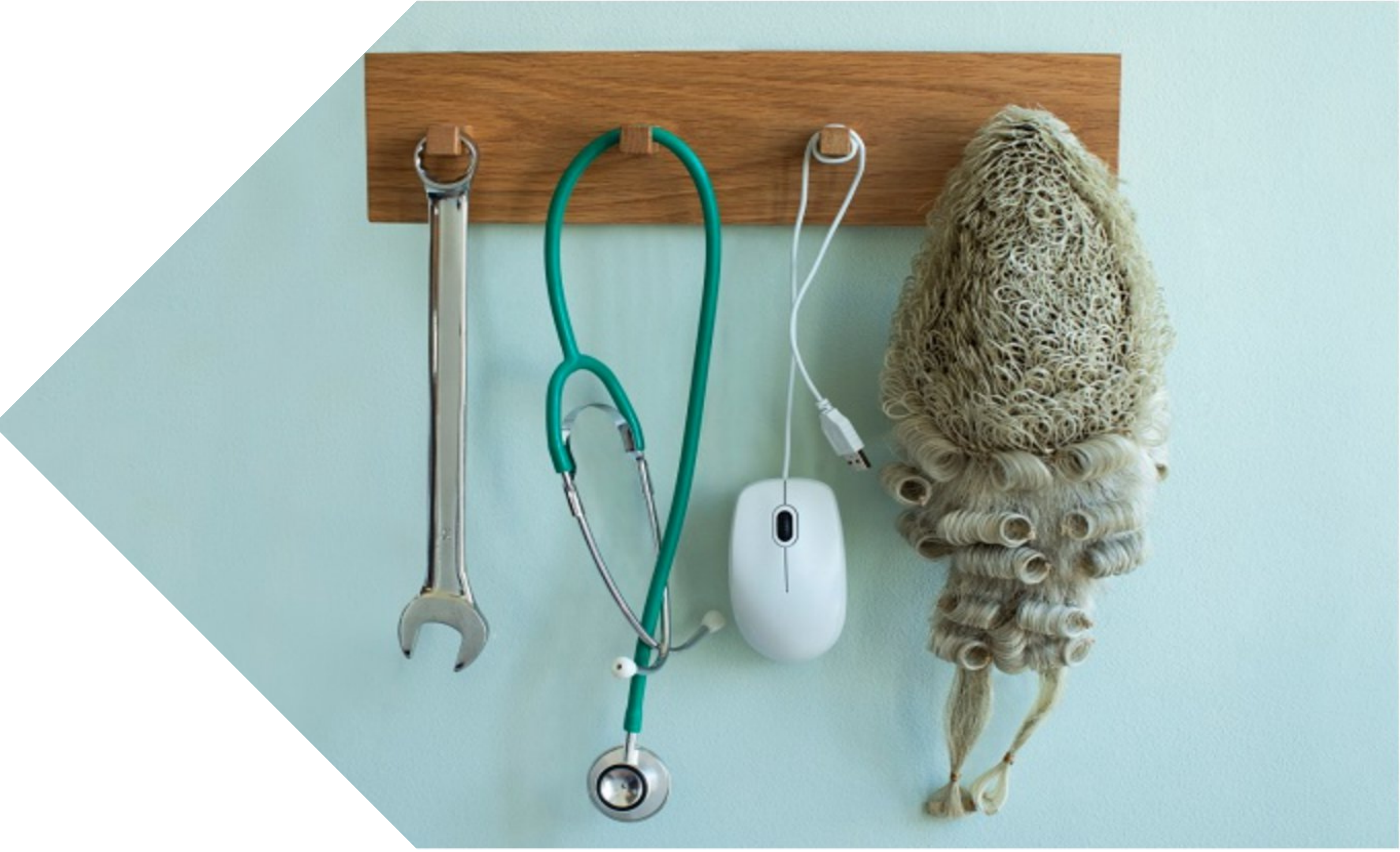

The scientific accompanying project „Process development and support for the use of AI in care“ within the funding initiative “Making Repositories and AI Systems Usable in Everyday Nursing Care” is funded by the Federal Ministry of Research, Technology and Space (Funding Code: 16SV8835). The funding body had no influence on the study design, data collection, analysis, interpretation, or the writing of the manuscript.

Authors:
Kathrin Seibert, Dominik Domhoff, Janissa Altona, Sebastian Jäger, Felix Bießmann, Alessia Nowak, Rahel Gubser, Matthias Schulte-Althoff, Daniel Fürstenau, Jörg Pohle, Lea Bergmann, Kathi Beier, Dagmar Borchers, Karin Wolf-Ostermann
With contributions from: David Walter, Richard Dulzon

University of Bremen – Faculty 11 Human and Health Sciences – Institute for Public Health and Nursing Research – Grazer Str. 4, 28359 Bremen – https://www.uni-bremen.de/en/institut-fuer-public-health-und-pflegeforschung

Gefördert durch:
Bundesministerium für Forschung, Technologie und Raumfahrt

ProKIP

KI-Pflege-Readiness-Assessment (KIP-RA)

User Manual

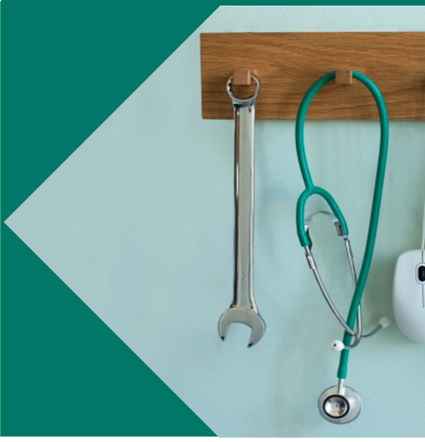

Ein Ergebnis der wissenschaftlichen Begleitforschung im BMBFTR-Förderprogramm „Repositorien und KI-Systeme im Pflegealltag nutzbar machen“

Juli 2025

Universität Bremen
vediso
BHT Berliner Hochschule für Technik
EINSTEIN CENTER Digital Future
Alexander von Humboldt Institut für Internet und Gesellschaft
CHARITÉ Universitätsmedizin Berlin

Gefördert durch:
Bundesministerium für Forschung, Technologie und Raumfahrt

Hintergrund des KI-Pflege-Readiness-Assessments (KIP-RA)

Künstliche Intelligenz (KI) kommt weltweit zur Unterstützung der körpernahen Versorgung und der Organisations- und Arbeitsprozesse in der Pflege zum Einsatz. KI-basierte Produkte sind bereits am Markt vertreten – etwa in Form einer sprachgesteuerten Pflegedokumentation, Sensorik zur Sturzerkennung, Software für intelligente Dienst- und Tourenplanung, klinische Entscheidungsunterstützungssysteme oder für eine (virtuelle) Aktivierung und digitale Förderung sozialer Teilhabe (Seibert, Domhoff et al. 2021).

Forschungsförderungsprogramme zielen darauf ab, das Potential von KI für die Pflege zu bewerten. Forschungs- und Entwicklungsprojekte zu KI in der Pflege (folgend KI-Pflege-Projekte oder KIP-Projekte) sollen Beiträge zur Lösung einer gesamtgesellschaftlichen Aufgabe liefern: Eine qualitativ hochwertige pflegerische Versorgung zu sichern, während ein zunehmendes Ungleichgewicht zwischen den Pflege- und Unterstützungsbedarfen schnell alternder Gesellschaften und dem verfügbaren Pflege- und Gesundheitspersonal die Erbringung bedarfsgerechter Pflege erschwert.

KIP-Projekte müssen neben technischen und regulatorischen Anforderungen auch prozessuale, ethische und soziale Aspekte des KI-Einsatzes berücksichtigen. Das gilt in allen Projektphasen: Bei der Planung, der Umsetzung und der Evaluation. Projektverantwortliche treffen auf unterschiedliche Pflegesettings mit vielen möglichen Anwendungsfällen für KI (wie KI für die Pflege im Krankenhaus, im Pflegeheim, in der ambulanten Pflege oder in der Aus-, Fort- und Weiterbildung), die mit jeweils ihrer ganz eigenen Organisationslogik und -kultur auf den Projektverlauf einwirken.

Im Auftrag des Bundesministeriums für Forschung, Technologie und Raumfahrt (BMFTR) führte die Universität Bremen in Kooperation mit dem Verband für Digitalisierung in der Sozialwirtschaft e.V. (vediso), dem Institut für Medizinische Informatik der Charité – Universitätsmedizin Berlin, der Berliner Hochschule für Technik und dem Alexander von Humboldt Institut für Internet und Gesellschaft mit Einstein Center Digital Future die wissenschaftliche Begleitforschung zum BMFTR-Förderprogramm *Repositorien und KI-Systeme im Pflegealltag nutzbar machen* durch. Als Ergebnis der Begleitforschung entstand ein Reifegradmodell, das KI-Pflege-Readiness-Assessment(KIP-RA).

Das KIP-RA soll Verantwortlichen aus KIP-Projekten, Pflegeeinrichtungen und Kliniken im Prozess der Planung, Umsetzung und Evaluation ihrer Projekte als Reflexionshilfe dienen. Mit dem KIP-RA können KIP-Projekte ihren Umgang mit wichtigen Voraussetzungen und Aspekten dieser Projekte reflektieren und erfolgversprechend gestalten.

Das KIP-RA umfasst unter anderem Themen wie das Nutzen von KI-Systemen in der Pflege, die Repräsentativität der verwendeten Daten, Ansätze zum Datenteilen, die Gestaltung von Partizipation sowie (pflege-)ethische und berufspraktische Implikationen des KI-Einsatzes.

An wen richtet sich das KIP-RA?

Das KIP-RA richtet sich an

- Verantwortliche in KIP- Projekten und
- Personen aus Pflegeeinrichtungen und Kliniken, die den Reifegrad ihres Unternehmens in Hinblick auf die Umsetzung von KIP-Projekten beurteilen wollen.

Verantwortliche in KIP-Projekten sind wissenschaftliche Projektleitungen, Mitarbeitende oder Projektbeauftragte in Pflegeeinrichtungen und Kliniken oder einzelne Personen, die sich in einem KIP-Projekt in einem Teilbereich (z.B. regulatorische Anforderungen) mit entsprechenden Fähigkeiten (Attributen) auseinandersetzen wollen oder müssen.

Was sind KI-Pflege-Projekte, KI-Systeme und Praxispartner?

KIP-Projekte sind Forschungs- und Entwicklungsprojekte, die darauf abzielen, ein KI-System (KI-basierte Pflegetechnologie) für und mit Praxispartnern aus Pflegeeinrichtungen und Kliniken zu entwickeln und zu erforschen oder ein bestehendes KI-System in der Pflegepraxis zu implementieren. KIP-Projekte können auch wissenschaftliche Projekte sein sowie von Pflegeeinrichtungen und Kliniken mit und ohne einen bestimmten Hersteller oder Anbieter von KI-Systemen selbst initiierte und umgesetzte Projekte.

KI-Systeme sind vom Menschen entwickelte Systeme, die dafür konzipiert sind, mit unterschiedlichen Autonomiegraden zu arbeiten und nach dem Einsatz Anpassungsfähigkeit zeigen können. KI-Systeme ziehen – zur Erreichung expliziter oder impliziter Ziele – aus den empfangenen Eingaben Schlüsse darüber, wie Ausgaben wie beispielsweise Vorhersagen, Inhalte, Empfehlungen oder Entscheidungen generiert werden sollen, die physische oder virtuelle Umgebungen beeinflussen können (European Commission 2025).

KI-Systeme in KIP-Projekten können auf Verfahren im Bereich maschinelles Lernen (ML) beruhen. Sie können aber auch auf einem Expertensystem oder einem hybriden KI-System (kombiniert ML mit einem Expertensystem) basieren.

Praxispartner in KIP-Projekten sind Einrichtungen der ambulanten, teilstationären und stationären Langzeitpflege und Krankenhäuser.

Wie wurde das KIP-RA entwickelt?

Das KIP-RA ist das Ergebnis eines mehrjährigen Entwicklungsprozesses. Diese Wissensquellen gingen in die Entwicklung des KIP-RA ein:

- internationale Empfehlungen für die Gestaltung und die Struktur von Reifegradmodellen (Akbarighatar et al., 2023; Becker et al., 2009; Lasrado et al., 2015),
- veröffentlichtes empirisches Wissen aus der Welt aus Studien zu Herausforderungen und fördernden Faktoren für KI in der Pflege (N=292) und zu KI-Bereitschaftsfaktoren (KI-Readiness-Faktoren) von Einrichtungen des Gesundheitswesens und der Pflege (N=7),
- Wissen aus Workshops (N=21) und Interviews (N=14) mit deutschen Expert:innen aus Pflegewissenschaft, Informatik, Pflegebildung, Pflegepraxis und Ethik mit und ohne Erfahrungen in der Durchführung von KIP-Projekten,
- Wissen aus einem Workshop mit Mitarbeitenden in deutschen KIP-Projekten (N=13),
- Wissen aus Think Aloud Interviews und Gruppendiskussionen mit deutschen Expert:innen aus Pflegewissenschaft, Informatik, Pflegebildung und Pflegepraxis mit Erfahrungen in der Durchführung von KIP-Projekten (N=18),
- Wissen des Studienteams aus Einblicken in die KIP-Projekte (N=8) des BMFTR-Förderprogramms *Repositorien und KI-Systeme im Pflegealltag nutzbar machen*.

Die Entwicklung des KIP-RA folgt einem sogenannten Bottom-Up-Ansatz (Lasrado et al., 2015). Dieser Ansatz kategorisiert zunächst KI-Readiness-Faktoren in Attribute und Fähigkeiten, leitet dann Reifegradstufen ab und entwickelt für diese Indikatoren. Das KIP-RA wurde iterativ entwickelt und berücksichtigt die Rückmeldungen von Expertinnen und Experten verschiedener Disziplinen mit Erfahrung in der Planung, Umsetzung und Evaluation von KIP-Projekten im gesamten Entwicklungsprozess.

Gefördert durch:
Bundesministerium für Forschung, Technologie und Raumfahrt

Welche Dimensionen und Attribute umfasst das KIP-RA?

Das KIP-RA umfasst fünf Dimensionen, die 69 Subdimensionen (Attribute für KI-Pflege-Readiness) enthalten. Die fünf Dimensionen stellen die verschiedenen Handlungsfelder dar, in denen KIP-Projekte Einfluss auf das Projektergebnis nehmen und sich die Praxispartner mit dem Reifengrad ihres Unternehmens für die Umsetzung von KIP-Projekten auseinandersetzen können.

Die Kurzüberblicke am Ende dieser Informationen zum KIP-RA zeigen Kurzbeschreibungen der fünf Dimensionen; eine Erläuterung der Reifegradstufen sowie eine dezidierte Übersicht aller 69 Subdimensionen. Das komplexe KIP-RA sieht die Bewertung jeder Dimension mit ihren Subdimensionen über fünf Reifegradstufen (Stufe 1 bis Stufe 5) vor. Dabei stellt Stufe 1 das geringste und Stufe 5 das höchste Ausmaß an KI-Pflege-Readiness dar.

Wann, wie und durch wen wird das KIP-RA angewendet?

Das KIP-RA ist zu einem beliebigen Zeitpunkt anlassbezogen sowohl in der Planungsphase als auch bei der begleitenden und abschließenden Evaluation von KIP-Projekten einsetzbar. Eine einmalige Erhebung mit dem KIP-RA liefert einen Ist-Zustand der KI-Pflege-Readiness zu einem beliebigen Zeitpunkt im Projektverlauf. Eine wiederholte Erhebung ermöglicht es, die Entwicklung einzelner Attribute im Zeitverlauf abzubilden.

Das KIP-RA kann als Selbst- oder als Fremdbewertungsinstrument genutzt werden. Die Bewertung mit dem KIP-RA findet idealerweise im interprofessionellen Team statt. Je nach Dimension und Attribut ist es ratsam, die Bewertung durch Personen aus diesen Fachbereichen vorzunehmen:

- **KI Forschung und Entwicklung (KI F&E):** Diejenigen, die für die technische Funktionalität des KI-Systems zuständig sind.
- **Pflegewissenschaft:** Diejenigen, die für die pflegewissenschaftliche Begleitung des KIP-Projektes zuständig sind oder im Entwicklungsprozess des KI-Systems die pflegewissenschaftliche Perspektive einbringen.
- **Praxispartner (mit IT-Fachpersonal):** Diejenigen, die das praktisch-klinische Einsatzfeld des KI-Systems in das KIP-Projekt einbringen, Personal, Strukturen und Prozesse des Praxispartners gut kennen oder im Entwicklungsprozess des KI-Systems die Perspektive des Praxispartners einbringen.

Die empfohlenen Bewertenden sind im KIP-RA ausgewiesen. Das KIP-RA kann entweder für ein gesamtes KIP-Projekt oder für einem Teilbereich eines KIP-Projekts (etwa auf Ebene der Subdimensionen) angewendet werden.

Gefördert durch:
Bundesministerium für Forschung, Technologie und Raumfahrt

Wie wird das KIP-RA interpretiert?

In Ermangelung eines international etablierten Konsenses darüber, welche Voraussetzungen und Aspekte mit welcher Gewichtung den Verlauf und Erfolg von KIP-Projekten beeinflussen, generiert das KIP-RA bewusst keinen Gesamtscore über einzelne oder alle Dimensionen. Stattdessen regt das KIP-RA dazu an, sich mit allen genannten Dimensionen und Attributen auseinanderzusetzen. Denn due im KIP-RA enthaltenen Voraussetzungen und Aspekte wurden sowohl in der gesichteten Literatur als auch in den verschiedenen Workshops und Interviews mit Expert:innen als wichtig für den Erfolg von KIP-Projekten beschrieben. Erfolg zeichnet sich in den meist interprofessionell besetzten KIP-Projekten, die einen hohen Abstimmungs- und Kommunikationsaufwand zwischen den einzelnen Projektbeteiligten erfordern, durch einen möglichst reibungslos voranschreitenden Projektverlauf mit Pilotierung, Testung oder Implementierung eines KI-Systems in der Pflegepraxis oder bis zum anvisierten übergeordneten Projektziel aus. Das Ergebnis der Bewertung mit dem KIP-RA liefert daher immer einen Hinweis darauf, wie ein KIP-Projekt zum Erhebungszeitpunkt bei der Bearbeitung von Voraussetzungen und Aspekten aufgestellt ist, von denen bekannt ist, dass sie Einfluss auf den Verlauf und den Erfolg von KIP-Projekten nehmen.

In der Kontextanalyse für die Planung eines KIP-Projektes, der Planungsphase und der formativen Evaluation können Projektverantwortliche das Bewertungsergebnis dazu nutzen, sich darüber zu verständigen, welche Voraussetzungen oder Aspekte sie mit welchen Maßnahmen künftig weiter vorantreiben möchten oder sogar vorantreiben müssen und welche Ergebnisse sie gegebenenfalls auch nicht weiter berücksichtigen wollen.

In der summativen Evaluation kann das Bewertungsergebnis Hinweise darauf liefern, welche Faktoren gegebenenfalls besonders Einfluss auf den Verlauf und den Erfolg eines KIP-Projektes genommen haben.

In jeder Projektphase kann das Bewertungsergebnis dabei helfen, konkrete Maßnahmen und Strategien abzustimmen, zu begründen und zu dokumentieren. Auf diese Weise trägt das KIP-RA dazu bei, den sachlichen Austausch im KIP-Projekt zu fördern.

Gefördert durch:
Bundesministerium für Forschung, Technologie und Raumfahrt

Kurzüberblick: Fünf Dimensionen des KIP-RA

Dimension	Beschreibung
**Regulatorische Voraussetzungen und Aspekte**<br>*9 Attribute*	Diese Dimension berücksichtigt die erforderlichen rechtlichen Rahmenbedingungen und Datenschutzregelungen, die eine Analyse des Datenbestandes und eine Auseinandersetzung mit Modellen des Datenteilens voraussetzen, um den Einsatz von KI-Systemen im Pflegealltag zu ermöglichen.
**Prozessuale und translationale Voraussetzungen und Aspekte**<br>*40 Attribute*	Diese Dimension erfasst die personellen, materiellen, zeitlichen und immateriellen Ressourcen für KIP-Projekte in Pflegeeinrichtungen oder Kliniken sowie deren generellen und KI-spezifischen Digitalisierungsgrad.<br>Auch der Reifegrad von übergeordneten Strategien für KI, Data-Governance und IT-Governance sowie für eine langfristige externe Begleitung und Evaluation des KI-Einsatzes wird in dieser Dimension ermittelt.<br>Die Auseinandersetzung mit der Umsetzung einer bedarfsorientierten Forschung und Entwicklung von KI-Systemen mit einem praktischen Nutzen und Mehrwert und mit vorhandenem KI-bezogenen Wissen und dazugehörigen Kompetenzen in Pflegeeinrichtungen und Kliniken sollen dazu beitragen, KI-Systeme nahtlos in bestehende Pflegeprozesse zu integrieren.<br>Diese Dimension sieht auch die Auseinandersetzung mit der Haltung zu KI und der Akzeptanz von und dem Vertrauen in KI-Technologien im Pflegealltag vor, um die Nutzung von KI-Systemen zu unterstützen und gegebenenfalls auf Bedenken der vom KI-Einsatz Betroffenen eingehen zu können.
**Technische Voraussetzungen und Aspekte**<br>*6 Attribute*	Diese Dimension befasst sich mit der Verfügbarkeit und Funktionalität der technischen Infrastruktur auch in Hinblick auf Datenschutz und Datensicherheit, die für eine verlässliche Datennutzung und Analyse erforderlich sind.<br>Um eine reibungslose, sichere und effiziente Datenintegration sowie einen Datenaustausch zwischen verschiedenen Systemen und Akteur:innen im Pflegealltag zu ermöglichen, erfasst diese Dimension auch die Angliederung der KI-Systeme an Dateninfrastrukturen oder Datenplattformen und die Nutzung technischer Interoperabilitätsstandards und Nomenklaturen.
**Soziale und ethische Voraussetzungen und Aspekte**<br>*11 Attribute*	Diese Dimension umfasst ethische und methodische Überlegungen zu Freiwilligkeit, Privatheit, Fairness und Transparenz sowie zu ethisch-normativen Wertorientierungen des Feldes und einzelner Praxispartner.<br>Eine Auseinandersetzung mit den Auswirkungen des KI-Einsatzes auf Mikro-, Makro- und Meso-Ebene sowie ein verantwortlicher Umgang mit Daten sollen die Nutzung von KI-Systemen im Pflegealltag unterstützen.
**Voraussetzungen und Aspekte des Community Building**<br>*3 Attribute*	Diese Dimension zielt auf die Förderung eines Netzwerks ab, das den Wissensaustausch und die kollektive Weiterentwicklung von KI-Systemen im Pflegealltag stärkt. Forschende, Entwickler:innen und Stakeholder:innen aus Pflegepraxis und Pflegemanagement sind an diesem Netzwerk beteiligt.

Gefördert durch:
Bundesministerium für Forschung, Technologie und Raumfahrt

Kurzüberblick: Stufen des des KIP-RA

Das KIP-RA sieht die Bewertung jeder Dimension mit ihren Subdimensionen über fünf Reifegradstufen (Stufe 1 bis Stufe 5) vor. Stufe 1 stellt das geringste und Stufe 5 das höchste Ausmaß an KI-Pflege-Readiness dar.

Die fünf Stufen samt zugehöriger Indikatoren für die Einordnung in eine Stufe sind:

Stufe 1 (initial)	Stufe 2 (erkundend)	Stufe 3 (entschlossen)	Stufe 4 (gesteuert)	Stufe 5 (optimiert)
Das zu bewertende Attribut ist …				
• unklar<br>• fehlend<br>• mangelhaft<br>• abgelehnt<br>• ignoriert<br>• nicht/ nie<br>o bekannt<br>o einheitlich<br>o abgestimmt<br>o verfügbar<br>o überlegt<br>o berücksichtigt<br>o akzeptiert<br>o bereit<br>o anerkannt	• in Planung<br>• in Überlegung<br>• sporadisch<br>• veraltet<br>• kaum<br>• verfügbar<br>• vorhanden<br>• nicht/ nie<br>o vollständig<br>o geklärt<br>o systematisiert<br>o festgelegt<br>o dokumentiert<br>o getestet<br>o evaluiert<br>o zögerlich<br>o akzeptiert<br>o bereit<br>o anerkannt	• betriebsintern geregelt (interne Standards)<br>• projektintern geregelt (interne Standards)<br>• teilweise<br>o systematisiert<br>o verfügbar<br>o vorhanden<br>o inkonsistent<br>• nicht immer<br>o gewährleistet<br>o festgelegt<br>o abgestimmt<br>• einigen Beteiligten bekannt<br>• unregelmäßig<br>• noch nicht<br>o automatisiert<br>o umgesetzt<br>o begonnen<br>o dokumentiert<br>• teilweise<br>o implementiert<br>o getestet<br>o evaluiert<br>• zunehmend<br>o akzeptiert<br>o bereit<br>o anerkannt	• betriebsübergreifend geregelt (nationale Standards)<br>• projektübergreifend geregelt (nationale Standards)<br>• durchgehend<br>o verfügbar<br>o zugänglich<br>o gewährleistet<br>o systematisiert<br>o kommuniziert<br>• konsistent<br>• eingebunden<br>• integriert<br>• zentralisiert<br>• regelmäßig<br>o aktualisiert<br>o überwacht<br>• allen Beteiligten bekannt<br>• weitgehend<br>o automatisiert<br>o umgesetzt<br>o implementiert<br>o dokumentiert<br>o getestet<br>o evaluiert<br>o akzeptiert<br>o bereit<br>o anerkannt	• Internationale Standards<br>• in Echtzeit verfügbar<br>• optimiert<br>• integriert<br>• effizient<br>• sicher<br>• extern zertifiziert<br>• nachgewiesen<br>• nachweisbar<br>• weiterentwickelt<br>• kontinuierlich angepasst<br>• vollständig<br>o umgesetzt<br>o implementiert<br>o dokumentiert<br>o getestet<br>o evaluiert<br>o akzeptiert<br>o bereit<br>• umfassend

Je nach KIP-Projekt treffen möglicherweise nicht alle für die Bewertung vorgeschlagenen Attribute zu. Eine Bewertung mit „Trifft nicht zu" ist daher immer möglich.

Kurzüberblick: Subdimensionen und Attribute des KI-Pflege-Readiness-Assessments (KIP-RA)

Dimension	Attribut-Nummer	Subdimension/ Attribut	(Gemeinsame) Bewertung durch	Quellen zum Attribut
1 Regulatorische Voraussetzungen und Aspekte	1.1	**Analyse des Datenbestandes: Informationsgehalt (trotz Anonymisierung)**	KI F&E<br>Pflegewissenschaft<br>Praxispartner	(Pumplun et al., 2021)
	1.2	**Analyse des Datenbestandes: Repräsentativität von Trainingsdaten**	KI F&E<br>Pflegewissenschaft	(Pumplun et al., 2021; Wolf-Ostermann et al., 2021)
	1.3	**Analyse des Datenbestandes: Qualität**	KI F&E<br>Praxispartner	(Pumplun et al., 2021; Wolf-Ostermann et al., 2021)
	1.4	**Analyse des Datenbestandes: Verfügbarkeit**	KI F&E<br>Praxispartner	(Alami et al., 2020; Pumplun et al., 2021; Weinert et al., 2022; Wolf-Ostermann et al., 2021)
	1.5	**Analyse des Datenbestandes: Zugang**	KI F&E<br>Praxispartner	(Alami et al., 2020; Chang, 2020; Wolf-Ostermann et al., 2021)
	1.6	**Modelle des Datenteilens**	KI F&E	(Alami et al., 2020; Chang, 2020; Wolf-Ostermann et al., 2021)
	1.7	**EU-Medizinprodukte-Verordnung EU-MPV (und/oder, wenn auch auslaufend, MPG)**	KI F&E<br>Pflegewissenschaft<br>Praxispartner	(Chang, 2020; Wiljer & Hakim, 2019; Wolf-Ostermann et al., 2021)
	1.8	**EU-DSGVO und Spezialgesetze**	KI F&E<br>Pflegewissenschaft<br>Praxispartner	(Chang, 2020; Wiljer & Hakim, 2019; Wolf-Ostermann et al., 2021)
	1.9	**EU-KI-Verordnung**	KI F&E<br>Pflegewissenschaft<br>Praxispartner	PROKIP

Dimension	Attribut-Nummer	Subdimension/ Attribut	(Gemeinsame) Bewertung durch	Quellen zum Attribut
2 Prozessuale und translationale Voraussetzungen und Aspekte	2.1	**Praxispartner: Zeitliche Ressourcen*** *Zeitliche Ressourcen können sich auf Führungskräfte und Pflegefachpersonen der Organisation beziehen, aber auch auf weiteres Personal (z.B. IT-Fachpersonal der Organisation, andere Berufsgruppen), das entsprechend einzuplanen ist.	Praxispartner	(Weinert et al., 2022)
	2.2	**Praxispartner: Personelle Ressourcen*: Eigene Personalstelle für die Implementierung von KI in der Organisation** *Personelle Ressourcen können sich auf Führungskräfte und Pflegefachpersonen der Organisation beziehen, aber auch auf weiteres Personal (z.B. IT-Fachpersonal der Organisation, andere Berufsgruppen), das entsprechend einzuplanen ist.	Praxispartner	(Abuzaid et al., 2022)
	2.3	**Praxispartner: Personelle Ressourcen: Eigene Personalstelle, die sich an Forschung und Entwicklung (F&E) von KI beteiligt**	Praxispartner	(Abuzaid et al., 2022)
	2.4	**Praxispartner: Personelle Ressourcen: Verfügbare personelle Ressourcen für KIP-Projekte und KI-Integration in der Organisation**	Praxispartner	(Weinert et al., 2022)
	2.5	**Praxispartner: Personelle Ressourcen: Verfügbare Data Scientists*** *Data Scientists nutzen Datenanalyse, maschinelles Lernen und Statistik auf der Datengrundlage des Praxispartners, um die pflegerische Versorgung zu verbessern, betriebliche Abläufe zu optimieren und Forschung zu unterstützen. Praxispartnern, die eigene Data Scientists beschäftigen, fällt es leichter, in der Organisation vorhandene unstrukturierte und strukturierte Daten für KIP-Projekte nutzbar zu machen.	Praxispartner mit IT-Fachpersonal KI F&E	(Chang, 2020)
	2.6	**Praxispartner: Personelle Ressourcen: Verfügbare Data Champions*** *Data Champions kennen die beim Praxispartner anfallenden Arten von Daten, setzen sich besonders für den Umgang mit Daten ein und vermitteln zwischen dem Pflegefachpersonal und der IT-Abteilung. Sie stellen sicher, dass verschiedene Arten von Daten korrekt, vollständig und aktuell sind. Sie identifizieren und beheben Probleme in der Datenerfassung. Sie fördern die Datenkompetenz der Mitarbeitenden durch Schulungen und sensibilisieren in der Organisation für die Bedeutung von Daten in der Versorgung und Forschung.	Praxispartner mit IT-Fachpersonal KI F&E	(Chang, 2020)
	2.7	**Praxispartner: Digitalisierungsgrad generell*** *Genereller Digitalisierungsgrad in dem Unternehmen des Praxispartners und in den unterschiedlichen Organisationsbereichen und Datengrundlagen (z.B. Personaldaten, Pflege-/Patient:innendaten, Verwaltungsdaten etc.)	Praxispartner mit IT-Fachpersonal KI F&E	(Chang, 2020; Pumplun et al., 2021; Weinert et al., 2022)
	2.8	**Praxispartner: Digitalisierungsgrad KI-spezifisch**	Praxispartner mit IT-Fachpersonal KI F&E	(Chang, 2020; Weinert et al., 2022)
	2.9	**Praxispartner: Digitalisierungsgrad: Qualitätsstandards für Daten**	Praxispartner mit IT-Fachpersonal KI F&E	(Pumplun et al., 2021)

Dimension	Attribut-Nummer	Subdimension/ Attribut	(Gemeinsame) Bewertung durch	Quellen zum Attribut
2 Prozessuale und translationale Voraussetzungen und Aspekte	2.10	**Praxispartner: Digitalisierungsgrad: Standard Datensammlung***<br>*Gemeint ist ein Standard für die Sammlung von Daten, die beim Praxispartner als Routinedaten im alltäglichen Pflege- und Organisationsprozess anfallen.	Praxispartner mit IT-Fachpersonal<br>KI F&E	(Alami et al., 2020)
	2.11	**Praxispartner: Digitalisierungsgrad: Standard Datenspeicherung**	Praxispartner mit IT-Fachpersonal<br>KI F&E	(Alami et al., 2020)
	2.12	**Praxispartner: Digitalisierungsgrad: Standard Datenaustausch**	Praxispartner mit IT-Fachpersonal<br>KI F&E	(Alami et al., 2020)
	2.13	**Praxispartner: Digitalisierungsgrad: Standard ontologische Repräsentation***<br>*Ontologische Repräsentation meint die strukturierte Darstellung von Wissen in einem bestimmten Fachgebiet (z. B. Medizin), um Daten einheitlich, interpretierbar und qualitativ hochwertig zu machen. Ein Standard für eine ontologische Repräsentation hilft dabei, Daten semantisch zu strukturieren und zu standardisieren, sodass sie verständlich, vergleichbar und interoperabel werden. Ein Krankenhaus kann z.B. verschiedene Begriffe für dieselbe Erkrankung an verschiedenen Dokumentationsorten verwenden: "Herzinfarkt", "Myokardinfarkt", "ICD-10: I21". Eine ontologische Repräsentation würde diese Begriffe einer einheitlichen, standardisierten Definition zuordnen. Dadurch könnten IT-Systeme, Gesundheitsfachpersonal oder auch KI-Entwickler:innen eindeutig erkennen, dass sie sich auf dieselbe Erkrankung beziehen.	Praxispartner mit IT-Fachpersonal<br>KI F&E	(Alami et al., 2020)
	2.14	**Praxispartner: Bereitschaft zur digitalen Transformation:<br>Haltung und Umgang mit KI in der Organisation**	Praxispartner mit IT-Fachpersonal<br>KI F&E	(Alami et al., 2020; Chang, 2020)
	2.15	**Praxispartner: Bereitschaft zur digitalen Transformation:<br>Unterstützung von KI durch Führungskräfte und Stakeholder:innen***<br>*Stakeholder:innen sind alle Einzelpersonen oder Gruppen, die das Arbeitsklima in einer Organisation beeinflussen – wie Führungskräfte, Teamleitungen, Mitarbeitende, Personalverantwortliche und mitunter auch externe Berater:innen – deren Handlungen, Entscheidungen und Interaktionen die Organisationskultur, Kommunikation, das Vertrauen, die Motivation und das gesamte Arbeitsumfeld prägen.	Praxispartner	(Chang, 2020)
	2.16	**Praxispartner: Bereitschaft zur digitalen Transformation:<br>Eigene KI-Strategie der Organisation**	Praxispartner	(Alami et al., 2020; Pumplun et al., 2021)

Dimension	Attribut-Nummer	Subdimension/ Attribut	(Gemeinsame) Bewertung durch	Quellen zum Attribut
2 Prozessuale und translationale Voraussetzungen und Aspekte	2.17	**Praxispartner: Bereitschaft zur digitalen Transformation: Eigene Data-Governance-Strategie der Organisation***<br>*Data Governance formalisiert Entscheidungsrechte, Verfahren und Kontrollen, um die bei der Verarbeitung sowie gemeinsamen Nutzung von Daten zwischen den beteiligten Akteuren auftretenden Interessenskonflikte so gut es geht aufzulösen. Dafür ist es erforderlich, sowohl den Wert der Daten möglichst optimal zu schöpfen als auch die mit ihrer Verarbeitung verbundenen Risiken je nach Perspektive der beteiligten Akteure zu kontrollieren.	Praxispartner	(Abuzaid et al., 2022; Alami et al., 2020; Chang, 2020; Pumplun et al., 2021; Weinert et al., 2022; Wiljer & Hakim, 2019)
	2.18	**Praxispartner: Bereitschaft zur digitalen Transformation: eigene IT-Governance-Strategie der Organisation***<br>*IT Governance ist der strategische Rahmen, der sicherstellt, dass die IT eines Unternehmens oder einer Organisation effektiv, sicher und regelkonform betrieben wird. Sie sorgt dafür, dass IT-Ressourcen optimal eingesetzt werden, um die Unternehmensziele zu unterstützen.	Praxispartner	(Alami et al., 2020)
	2.19	**Praxispartner: Technikakzeptanz: Akzeptanz von KI durch Führungskräfte, Pflegefachpersonen und andere Stakeholder:innen***<br>*Stakeholder:innen sind alle weiteren relevanten Personengruppen, die nicht die Pflegeempfänger:innen oder Patient:innen sind. Je nach KIP-Projekt können das beispielsweise gesetzliche Betreuer:innen, Hausärzt:innen oder Konsildienste sein. Eine Analyse der relevanten Stakeholder:innen sollte Teil jeder Planung eines KIP-Projektes sein.	Praxispartner	(Alami et al., 2020)
	2.20	**Praxispartner: Technikakzeptanz: Akzeptanz von KI durch Pflegeempfänger:innen, Patient:innen und ihre An- und Zugehörigen**	Praxispartner<br>Pflegewissenschaft	(Chang, 2020; Pumplun et al., 2021; Wiljer & Hakim, 2019)
	2.21	**Praxispartner: Technikakzeptanz: Akzeptanz von KI durch Personalvertretungen und deren Einbezug**	Praxispartner<br>Pflegewissenschaft	(Pumplun et al., 2021; Wiljer & Hakim, 2019)
	2.22	**Praxispartner: Erwartungen und Vorbehalte**	Praxispartner<br>Pflegewissenschaft	PROKIP
	2.23	**Praxispartner: Wissen und Kompetenzen: Aus-, Fort- und Weiterbildung zu KI und Digitaler Kompetenz in der Organisation**	Praxispartner<br>Pflegewissenschaft<br>KI F&E	(Chang, 2020; Pumplun et al., 2021)
	2.24	**Praxispartner: Wissen und Kompetenzen: Verfügbares KI-Wissen des Personals**	Praxispartner	(Alami et al., 2020; Andersson et al., 2021)
	2.25	**Praxispartner: Wissen und Kompetenzen: Echtzeit- und/oder prädiktive Analysen**	Praxispartner	(Andersson et al., 2021; Pumplun et al., 2021; Weinert et al., 2022; Wiljer & Hakim, 2019)

Dimension	Attribut-Nummer	Subdimension/ Attribut	(Gemeinsame) Bewertung durch	Quellen zum Attribut
2 Prozessuale und translationale Voraussetzungen und Aspekte	2.26	**Praxispartner: Wissen und Kompetenzen:**<br>**Verfügbare Verfahren für den Wissenstransfer in der Organisation**	Praxispartner	(Chang, 2020)
	2.27	**Praxispartner: Immaterielle Güter:**<br>**Verfügbarkeit und Ausmaß immaterieller Güter im Kontext KI in der Organisation (Bücher, Fachbeiträge, Preise, strategische Partnerschaften, Konsultationen …) ***<br>*Auch immaterielle Güter in Bezug auf Digitalisierung und zu digitalen Kompetenzen können für Praxispartner in KIP-Projekten wertvoll sein.	Praxispartner	(Wiljer & Hakim, 2019)
	2.28	**Praxispartner: Finanzielle Ressourcen und Investitionen:**<br>**Verfügbare finanzielle Ressourcen für KIP-Projekte und KI-Integration***<br>*Finanzielle Ressourcen für KIP-Projekte können auch in allgemeinen Digitalisierungsbudgets oder anderen Budgets (Innovationsbudget, Teilhabebudget) enthalten sein.	Praxispartner	(Chang, 2020)
	2.29	**Finanzielle Ressourcen und Investitionen:**<br>**Prüfung alternativer Finanzierungsformate für die Einbindung von Praxispartnern**	Praxispartner	(Alami et al., 2020; Chang, 2020; Pumplun et al., 2021; Weinert et al., 2022)
	2.30	**Finanzielle Ressourcen und Investitionen:**<br>**Management: Nachhaltige Passung von Ressourcen und Investitionen**	KI F&E<br>Pflegewissenschaft<br>Praxispartner	(Wolf-Ostermann et al., 2021)
	2.31	**Forschungsziel: Bedarfe oder Probleme der Pflegepraxis**	KI F&E<br>Pflegewissenschaft<br>Praxispartner	(Alami et al., 2020)
	2.32	**Praktischer Nutzen und Mehrwert des KI-Systems**	KI F&E<br>Pflegewissenschaft<br>Praxispartner	(Alami et al., 2020; Chang, 2020)
	2.33	**Umsetzung realistischer, Feld-erprobter Projekte vor "Grand Vision" Projekten**	Pflegewissenschaft<br>KI F&E<br>Praxispartner	(Alami et al., 2020; Chang, 2020)
	2.34	**Strategien für die Partizipation und Information von Stakeholder:innen**	Pflegewissenschaft<br>KI F&E<br>Praxispartner	(Chang, 2020)

Dimension	Attribut-Nummer	Subdimension/ Attribut	(Gemeinsame) Bewertung durch	Quellen zum Attribut
2 Prozessuale und translationale Voraussetzungen und Aspekte	2.35	**Strategien zur Vertrauensbildung, Abstimmung von Erwartungen und zum Abbau von Vorbehalten**	Pflegewissenschaft<br>KI F&E<br>Praxispartner	(Wiljer & Hakim, 2019)
	2.36	**Reflexion der Bedeutung der Einbindung von Menschen als vermittelnde Instanz zwischen KI-System und Handlung und gegebenenfalls daraus resultierender Entwicklungskonsequenzen**	Pflegewissenschaft<br>KI F&E<br>Praxispartner	(Chang, 2020)
	2.37	**Strategien für eine langfristige externe Begleitung und Evaluation des KI-Einsatzes**	KI F&E<br>Pflegewissenschaft<br>Praxispartner	(Wolf-Ostermann et al., 2021)
	2.38	**Strategien für eine langfristige externe Begleitung: Software/ Hardware: Updates**	KI F&E	(Wolf-Ostermann et al., 2021)
	2.39	**Strategien für eine langfristige externe Begleitung: Software/ Hardware: Upgrades**	KI F&E	(Alami et al., 2020)
	2.40	**Strategien für eine langfristige externe Begleitung: Software/ Hardware: Maintenance**	KI F&E	(Alami et al., 2020)

Gefördert durch:
Bundesministerium für Forschung, Technologie und Raumfahrt

Dimension	Attribut-Nummer	Subdimension/ Attribut	(Gemeinsame) Bewertung durch	Quellen zum Attribut
3 Technische Voraussetzungen und Aspekte	3.1	**Angliederung an existierende Dateninfrastrukturen und -plattformen**	KI F&E	(Wolf-Ostermann et al., 2021)
	3.2	**Nutzung technischer Interoperabilitätsstandards und Nomenklaturen**	KI F&E<br>Pflegewissenschaft	(Alami et al., 2020; Chang, 2020; Wolf-Ostermann et al., 2021)
	3.3	**IT-Sicherheit: Absicherung kritischer Infrastruktur und Verschlüsselungstechniken: Verfügbare Cyber Security [beim Praxispartner]**	KI F&E<br>Praxispartner	(Alami et al., 2020; Chang, 2020)
	3.4	**IT-Sicherheit: Sicherheitszertifizierungen**	KI F&E	(Chang, 2020; Wiljer & Hakim, 2019)
	3.5	**Praxispartner: Digitale Infrastruktur: Technische Infrastruktur**	KI F&E	(Alami et al., 2020; Chang, 2020; Wolf-Ostermann et al., 2021)
	3.6	**IT-Infrastruktur: KI Compute: Hardware**	KI F&E	PROKIP

Dimension	Attribut-Nummer	Subdimension/ Attribut	(Gemeinsame) Bewertung durch	Quellen zum Attribut
**4 Soziale und ethische Voraussetzungen und Aspekte**	**4.1**	**Ethikvotum**	KI F&E<br>Pflegewissenschaft	(Wolf-Ostermann et al., 2021)
	**4.2**	**Informierte Einwilligung (Informed Consent) und Andauernde Einwilligung (Ongoing Consent): Möglichkeiten und Grenzen eines Informed oder Ongoing Consent und alternative Lösungen**	KI F&E<br>Pflegewissenschaft	(Wolf-Ostermann et al., 2021)
	**4.3**	**Möglichkeiten und Grenzen von zentralen und dezentralen Methoden zur Sicherstellung der Privatsphäre von Studienteilnehmenden***<br>*Studienteilnehmende sind alle Personen, von denen im KIP-Projekt Daten zur Entwicklung oder Evaluation des KI-Systems erhoben werden. Auch Pflegefachpersonen, die z.B. an Fokusgruppen teilnehmen, sind Studienteilnehmende.	KI F&E<br>Pflegewissenschaft	(Wolf-Ostermann et al., 2021)
	**4.4**	**Auseinandersetzung mit ethisch-normativen Wertorientierungen des Feldes und einzelner Praxispartner**	KI F&E<br>Pflegewissenschaft<br>Praxispartner	(Chang, 2020; Wiljer & Hakim, 2019; Wolf-Ostermann et al., 2021)
	**4.5**	**Reflextion von Auswirkungen des KI-Einsatzes auf die direkte Arbeitsumgebung von Pflegefachpersonen**	KI F&E<br>Pflegewissenschaft<br>Praxispartner	(Wolf-Ostermann et al., 2021)
	**4.6**	**Reflextion von Auswirkungen des KI-Einsatzes auf das Berufsbild von Pflegefachpersonen**	KI F&E<br>Pflegewissenschaft<br>Praxispartner	(Wolf-Ostermann et al., 2021)
	**4.7**	**Strategien für die systematische Erfassung von erwünschten und unerwünschten Wirkungen des KI-Einsatzes**	KI F&E<br>Pflegewissenschaft	(Wolf-Ostermann et al., 2021)
	**4.8**	**Reflextion der Repräsentativität der genutzten Daten und der daraus abgeleiteten Bewertung des KI-Systems sowie der Übertragbarkeit der Ergebnisse des Forschungsprojektes**	KI F&E<br>Pflegewissenschaft	(Wolf-Ostermann et al., 2021)
	**4.9**	**Strategien, die die Transparenz und Erklärbarkeit von durch KI-Systeme getroffene Entscheidungen und Handlungsempfehlungen steigern**	KI F&E<br>Pflegewissenschaft	(Chang, 2020; Wolf-Ostermann et al., 2021)

Dimension	Attribut-Nummer	Subdimension/ Attribut	(Gemeinsame) Bewertung durch	Quellen zum Attribut
4 Soziale und ethische Voraussetzungen und Aspekte	4.10	**Verantwortlicher Umgang mit Daten: Individueller Consent, Datenspende, Research Exemption oder Data Trusteeship*** * Wenn Daten gesammelt oder verwendet werden – zum Beispiel Daten aus der Pflegedokumentation, App-Nutzungsdaten oder Bewegungsdaten – sollte das verantwortungsvoll geschehen. Es geht darum, die Privatsphäre von Individuen zu schützen und fair mit ihren Informationen umzugehen. Dafür gibt es verschiedene Konzepte. Individueller Consent: Personen entscheiden selbst, ob und welche ihrer Daten verwendet werden dürfen. Datenspende: Personen stellen ihre Daten freiwillig für die Forschung zur Verfügung. Research Exemption (Forschungs-Ausnahme): In bestimmten Fällen ist die Nutzung der Daten durch Forschende auch ohne direkte Zustimmung möglich, etwa, wenn das öffentliche Interesse besonders groß ist und der Datenschutz trotzdem gewahrt bleibt. Beispiel: Gesundheitsdaten aus einem Krankenhaus werden anonymisiert verwendet, um eine Pandemie zu erforschen. Data Trusteeship (Datentreuhänderschaft): Ein neutraler Dritter (z. B. eine Stiftung oder Behörde) verwaltet die Daten und gibt sie nur weiter, wenn bestimmte Regeln erfüllt sind.	KI F&E	(Wolf-Ostermann et al., 2021)
	4.11	**Beachtung der Positionierung und Wertorientierung der Pflege**	KI F&E<br>Pflegewissenschaft<br>Praxispartner	PROKIP

Dimension	Attribut-Nummer	Subdimension/ Attribut	(Gemeinsame) Bewertung durch	Quellen zum Attribut
5 Voraussetzungen und Aspekte des Community Building	5.1	**Technologischer Wissenstransfer**	KI F&E<br>Pflegewissenschaft	(Wolf-Ostermann et al., 2021)
	5.2	**Beteiligung an Onlineaustausch**	KI F&E<br>Pflegewissenschaft<br>Praxispartner	(Wolf-Ostermann et al., 2021)
	5.3	**Strategische Partnerschaften**	KI F&E<br>Pflegewissenschaft<br>Praxispartner	(Wiljer & Hakim, 2019)

Literatur

Abuzaid, M. M., Elshami, W., Tekin, H., & Issa, B. (2022). Assessment of the Willingness of Radiologists and Radiographers to Accept the Integration of Artificial Intelligence Into Radiology Practice. Acad Radiol, 29(1), 87-94. https://doi.org/doi:10.1016/j.acra.2020.09.014

Akbarighatar, P., Pappas, I., & Vassilakopoulou, P. (2023). A sociotechnical perspective for responsible AI maturity models: Findings from a mixed-method literature review. International Journal of Information Management Data Insights, 3(2). https://doi.org/10.1016/j.jjimei.2023.100193

Alami, H., Lehoux, P., Denis, J. L., Motulsky, A., Petitg, , C., Savoldelli, M., Rouquet, R., Gagnon, M. P., Roy, D., & Fortin, J. P. (2020). Organizational readiness for artificial intelligence in health care: insights for decision-making and practice. J Health Organ Manag. https://doi.org/doi:10.1108/jhom-03-2020-0074

Andersson, J., Nyholm, T., Ceberg, C., Almén, A., Bernhardt, P., Fransson, A., & Olsson, L. E. (2021). Artificial intelligence and the medical physics profession - A Swedish perspective. Phys Med, 88, 218-225. https://doi.org/doi:10.1016/j.ejmp.2021.07.009

Becker, J., Knackstedt, R., & Pöppelbuß, J. (2009). Developing Maturity Models for IT Management. Business & Information Systems Engineering, 1(3), 213-222. https://doi.org/10.1007/s12599-009-0044-5

Chang, A., Implementation of Artificial Intelligence in Medicine, in Intelligence-Based Medicine. Artificial Intelligence and Human Cognition in Clinical Medicine and Healthcare. 2020, Academic Press: London, San Diego, Cambridge, Oxford. p. 397-412.

European Commission (2025). ANNEX to the Communication to the Commission Approval of the content of the draft Communication from the Commission - Commission Guidelines on the definition of an artificial intelligence system established by Regulation (EU) 2024/1689 (AI Act). E. Commission.

Lasrado, L. A., Vatrapu, R., & Andersen, K. N. (2015). Maturity Models Development in IS Research: A Literature Review Selected Papers of the Informations Systems Research Seminar in Scandinavia, http://aisel.aisnet.org/iris2015/6

Pumplun, L., Fecho, M., Wahl, N., Peters, F., & Buxmann, P. (2021). Adoption of Machine Learning Systems for Medical Diagnostics in Clinics: Qualitative Interview Study. J Med Internet Res, 23(10), e29301. https://doi.org/doi:10.2196/29301

Seibert, K., D. Domhoff, D. Bruch, M. Schulte-Althoff, D. Furstenau, F. Biessmann and K. Wolf-Ostermann (2021). "Application Scenarios for Artificial Intelligence in Nursing Care: Rapid Review." J Med Internet Res 23(11): e26522.

Weinert, L., Müller, J., Svensson, L., & Heinze, O. (2022). Perspective of Information Technology Decision Makers on Factors Influencing Adoption and Implementation of Artificial Intelligence Technologies in 40 German Hospitals: Descriptive Analysis. JMIR Medical Informatics, 10(6). https://doi.org/doi:10.2196/34678

Wiljer, D., & Hakim, Z. (2019). Developing an Artificial Intelligence–Enabled Health Care Practice: Rewiring Health Care Professions for Better Care. Journal of Medical Imaging and Radiation Sciences, 50(4), S8-S14. https://doi.org/doi:10.1016/j.jmir.2019.09.010

Wolf-Ostermann, K., Fürstenau, D., Theune, S., Bergmann, L., Bießmann, F., Domhoff, D., Schulte-Althoff, M., & Seibert, K. (2021). Konzept zur Einbettung von KI-Systemen in der Pflege: Sondierungsprojekt zu KI in der Pflege (SoKIP).

Gefördert durch:
Bundesministerium für Forschung, Technologie und Raumfahrt

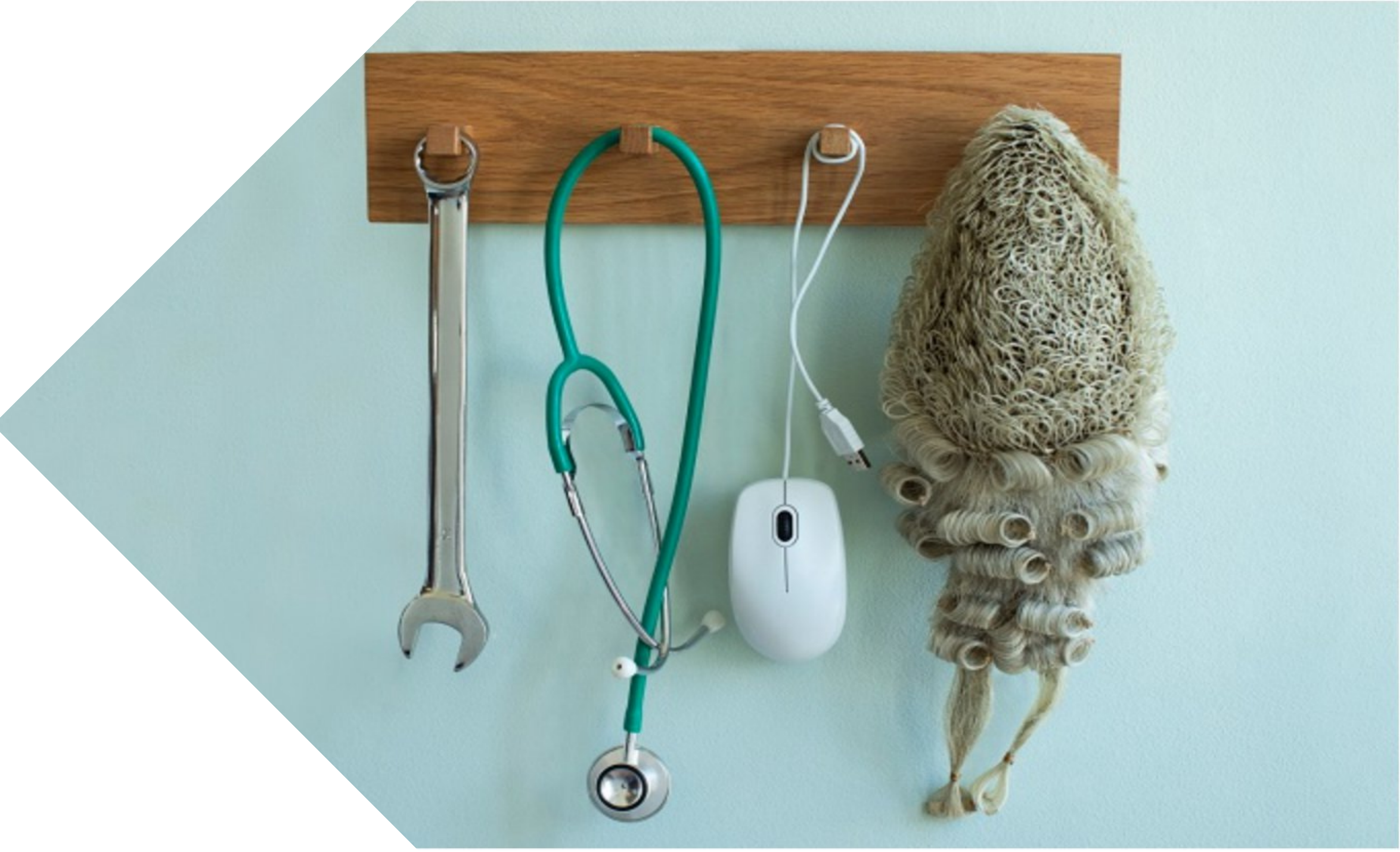

ProKIP – Prozessentwicklung und -begleitung zum KI-Einsatz in der Pflege – ist das wissenschaftliche Begleitprojekt in der vom Bundesministerium für Forschung, Technologie und Raumfahrt geförderten Bekanntmachung Repositorien und KI-Systeme im Pflegealltag nutzbar machen (Förderkennzeichen 16SV8835). Die Fördermittelgeber hatten keinen Einfluss auf die Studienplanung, Datenerhebung, -analyse und die Interpretation der Ergebnisse oder auf das Verfassen des Manuskripts.

Autor*innen:

Kathrin Seibert, Dominik Domhoff, Janissa Altona Sebastian Jäger, Felix Bießmann, Alessia Nowak, Rahel Gubser, Matthias Schulte-Althoff, Daniel Fürstenau, Jörg Pohle, Lea Bergmann, Kathi Beier, Dagmar Borchers, Karin Wolf-Ostermann

Unter Mitarbeit von: David Walter, Richard Dulzon

Universität Bremen - Fachbereich 11 Human- und Gesundheitswissenschaften - Institut für Public Health und Pflegeforschung - Grazer Str. 4, 28359 Bremen - https://www.uni-bremen.de/institut-fuer-public-health-und-pflegeforschung

Universität Bremen · vediso · BHT Berliner Hochschule für Technik · EINSTEIN CENTER Digital Future · Alexander von Humboldt Institut für Internet und Gesellschaft · CHARITÉ Universitätsmedizin Berlin
